# Human iPSC-Derived Hippocampal Spheroids: An Innovative Tool for Stratifying Alzheimer Disease Patient-Specific Cellular Phenotypes and Developing Therapies

**DOI:** 10.1016/j.stemcr.2020.06.001

**Published:** 2020-06-25

**Authors:** Yuriy Pomeshchik, Oxana Klementieva, Jeovanis Gil, Isak Martinsson, Marita Grønning Hansen, Tessa de Vries, Anna Sancho-Balsells, Kaspar Russ, Ekaterina Savchenko, Anna Collin, Ana Rita Vaz, Silvia Bagnoli, Benedetta Nacmias, Claire Rampon, Sandro Sorbi, Dora Brites, György Marko-Varga, Zaal Kokaia, Melinda Rezeli, Gunnar K. Gouras, Laurent Roybon

**Affiliations:** 1 iPSC Laboratory for CNS Disease Modeling, Department of Experimental Medical Science, BMC D10, Lund University, Lund SE-221 84, Sweden; 2 Strategic Research Area MultiPark, Lund University, Lund SE-221 84, Sweden; 3 Lund Stem Cell Center, Lund University, Lund SE-221 84, Sweden; 4 Medical Microspectroscopy, Department of Experimental Medical Science, BMC B11, Lund University, Lund SE-221 84, Sweden; 5 Experimental Dementia Research Unit, Department of Experimental Medical Science, BMC B11, Lund University, Lund SE-221 84, Sweden; 6 Clinical Protein Science and Imaging, Department of Biomedical Engineering, BMC D13, Lund University, Lund SE-221 84, Sweden; 7 Laboratory of Stem Cells and Restorative Neurology, Department of Clinical Sciences, BMC B10, Lund University, Lund SE-221 84, Sweden; 8 Department of Clinical Genetics and Pathology, Office for Medical Services, Lund SE-221 85, Sweden; 9 Research Institute for Medicines (iMed.ULisboa), Faculty of Pharmacy, Universidade de Lisboa, Lisbon, Portugal; 10 Department of Biochemistry and Human Biology, Faculty of Pharmacy, Universidade de Lisboa, Lisbon, Portugal; 11 Laboratorio di Neurogenetica, Dipartimento di Neuroscienze, Psicologia, Area del Farmaco e Salute del Bambino- NEUROFARBA, Università degli Studi di Firenze, Florence 50134, Italy; 12 IRCCS Fondazione Don Carlo Gnocchi, Florence, Italy; 13 Centre de Recherches sur la Cognition Animale (CRCA), Centre de Biologie Intégrative (CBI), Université de Toulouse; CNRS, UPS, Toulouse Cedex 9, France

**Keywords:** Alzheimer disease, iPSC, hippocampus, spheroids, proteomics, transcriptomics, viral-mediated gene therapy, protein aggregation, NeuroD1

## Abstract

The hippocampus is important for memory formation and is severely affected in the brain with Alzheimer disease (AD). Our understanding of early pathogenic processes occurring in hippocampi in AD is limited due to tissue unavailability. Here, we report a chemical approach to rapidly generate free-floating hippocampal spheroids (HSs), from human induced pluripotent stem cells. When used to model AD, both APP and atypical PS1 variant HSs displayed increased Aβ42/Aβ40 peptide ratios and decreased synaptic protein levels, which are common features of AD. However, the two variants differed in tau hyperphosphorylation, protein aggregation, and protein network alterations. NeuroD1-mediated gene therapy in HSs-derived progenitors resulted in modulation of expression of numerous genes, including those involved in synaptic transmission. Thus, HSs can be harnessed to unravel the mechanisms underlying early pathogenic changes in the hippocampi of AD patients, and provide a robust platform for the development of therapeutic strategies targeting early stage AD.

## Highlights

• Rapid generation of hippocampal spheroids (HSs) from hiPSCs using defined chemical agents
• hiPSC-derived HSs can be utilized as a source of hippocampal neurons
• hiPSC-derived HSs can be used to model Alzheimer disease
• hiPSC-derived HSs can be used to develop innovative therapeutic solutions

## Graphical Abstract

**Figure:**
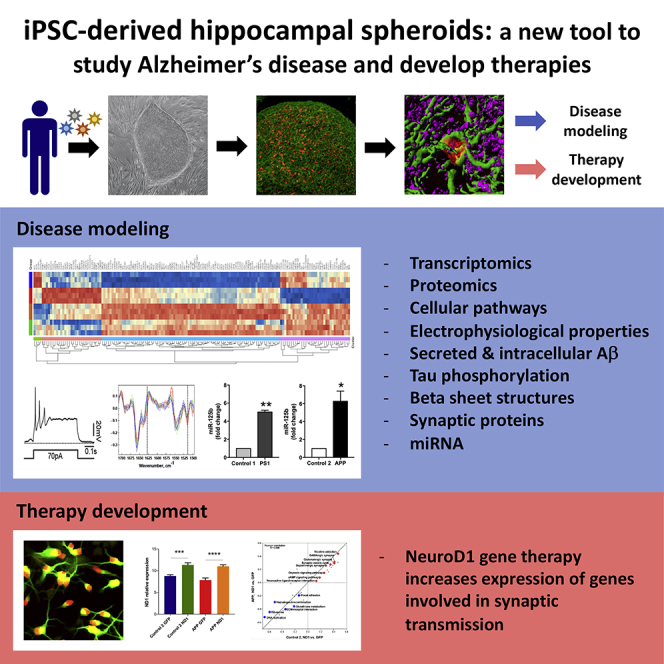

## Teaser

In this article, Roybon and colleagues developed a protocol to efficiently and rapidly generate hippocampus neurons from human induced pluripotent stem cells, which they used to model Alzheimer disease and develop a gene therapy approach to modulate the expression of genes involved in synaptic transmission.

## Introduction

Alzheimer disease (AD) is the most common cause of dementia in the elderly, resulting in memory impairments and cognitive decline, and eventually leading to significant disability. Despite decades of intensive research, AD still remains incurable and represents a major clinical, social and economic problem (https://www.alzheimers.net/resources/alzheimers-statistics/).

The hippocampus is involved in the formation of new memories, learning, and emotions, and is one of the first regions of the brain that atrophies in AD (Mueller et al., 2010). However, since human brain tissue is generally available only postmortem, our understanding of early pathogenic events occurring in the hippocampus, specifically at the cellular level, is limited to brain imaging techniques (Boutet et al., 2014, Schuff et al., 2009). Hence, it is difficult to identify the mechanisms underlying cognitive impairment in AD. Transgenic rodents are commonly used as an experimental model of AD for preclinical investigation (Richetin et al., 2015, Tampellini et al., 2010). However, all therapies that were successful in rodent models have failed in human clinical trials, questioning whether rodent AD models are appropriate for modeling human AD (Morris et al., 2014).

Therefore, to develop efficient therapies for AD, we need to develop advanced models for preclinical investigations. These models should closely recapitulate human AD pathology, and allow investigation of early cellular changes, to identify druggable targets that could delay disease onset, and eventually overcome memory impairment. Even if minimalist, the models should mimic the human brain parenchyma and allow 3D interaction between mature cell types (Clevers, 2016, Sloan et al., 2017). Such models should be inexhaustible and scalable to provide enough material for different assays, including omics analyses. They should also be useful to develop therapeutic strategies. Induced pluripotent stem cells (iPSCs) harboring the genetic background of the patient they are derived from, are an attractive source of human material that can be used to generate physiologically relevant models for studying neurological diseases, including AD (Arber et al., 2017). Given the crucial role played by the hippocampus in AD, the development of human iPSC-based hippocampal cellular models would be an important step forward for AD research.

Here, we describe an innovative strategy allowing to differentiate human iPSCs into hippocampal spheroids (HSs), enriched in hippocampal neurons expressing the zinc finger and BTB domain-containing protein 20 (ZBTB20) and the prospero homeobox protein 1 (PROX1) (Lavado and Oliver, 2007, Nielsen et al., 2014). The HSs generated from two AD patients carrying variations in amyloid precursor protein (APP) or presenilin 1 (PS1) genes exhibited cardinal cellular pathological features of AD, including loss of synaptic proteins and increased ratio of intracellular and extracellular Aβ42/Aβ40 peptides. However, they also exhibited differences in protein aggregation measured by the non-destructive label-free Fourier transform infrared (FTIR) microspectroscopy (Baker et al., 2014), tau phosphorylation, miRNA pattern, and protein network alterations. Hippocampal neurons derived from APP variant HSs demonstrated profound transcriptomic alterations, which could be modulated by overexpression of NeuroD1 (ND1), resulting in the upregulation of genes, gene products of which are associated with synaptic transmission and are altered in AD (de Wilde et al., 2016).

## Results

### Generation and Characterization of HSs from iPSCs of AD Patients and Healthy Individuals

To investigate the effect of PS1 and APP variation in human hippocampal cells, we generated iPSC lines from the skin fibroblasts of two patients, one female and one male, diagnosed with genetic AD (Figure S1), using well-established methodology (Djelloul et al., 2015, Holmqvist et al., 2016). The female patient carried a homozygous variation in the APP gene (APP p.V717I) (Sorbi et al., 1993). This variation, known as the AD London mutation, is the most common missense variation of the APP gene. The male patient with atypical AD carried a rare variation in the PS1 gene (PS1 p.R278K) (Assini et al., 2003). In addition, we generated multiple iPSC lines from age- and gender-matched non-demented healthy individuals.

**Figure 1:**
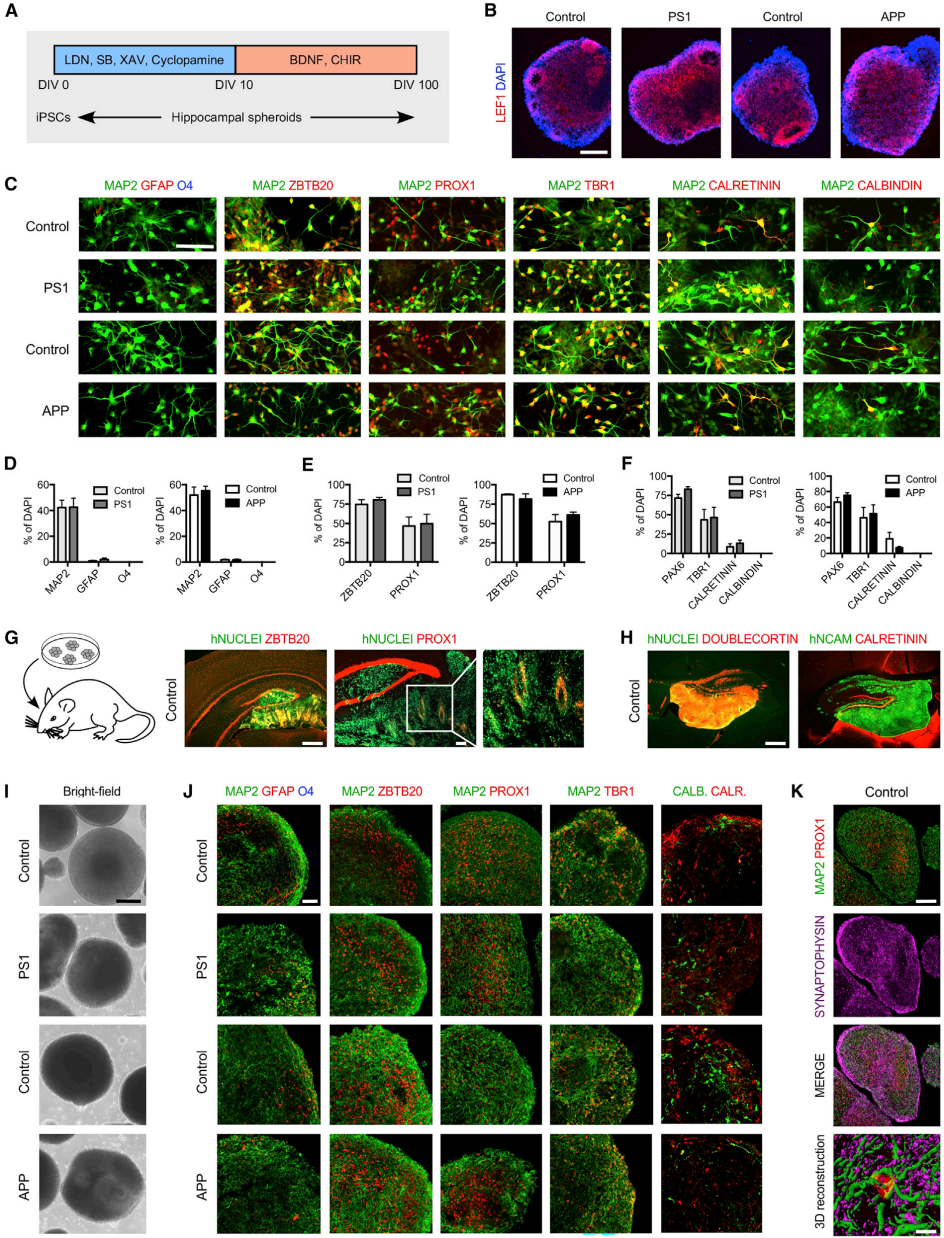
Generation and Characterization of HSs from AD Patient and Control Human iPSCs (A) Schematic model of the differentiation paradigm to generate HSs from iPSCs. LDN, LDN-193189, SB, SB-431542, XAV, XAV-939, CHIR, and CHIR-99021. (B) Immunostaining for medial pallium marker LEF1 in APP variant, PS1 variant, and gender-matched control EBs aged 30 DIV. Scale bar, 150 μm. (C) Immunostaining for brain cell subtype markers GFAP, O4 and MAP2, hippocampal markers ZBTB20 and PROX1, and neuronal markers TBR1, calretinin, and calbindin in APP variant, PS1 variant, and gender-matched control cultures at DIV 56. Scale bar, 200 μm. (D–F) Quantification of GFAP-positive, O4-positive, and MAP2-positive cells (D), ZBTB20-positive and PROX1-positive cells (E), and PAX6-positive, TBR1-positive, calretinin-positive, and calbindin-positive cells (F) expressed relative to the total number of DAPI-labeled cells in APP variant, PS1 variant, and gender-matched control cultures at DIV 56. Results are presented as mean ± SEM. n = 1–4 independent differentiations per clone for N = 3 iPSC clones per genotype. ∗p < 0.05; N/D means not detected. Statistical analysis by two-tailed t test. (G) Immunostaining for human nuclei marker and hippocampal markers ZBTB20 and PROX1 in control cells 5 weeks after transplantation into mouse hippocampus. Host mouse ZBTB20 and PROX1-positive cells are not colocalized with human nuclei marker. Scale bars, 100 μm. (H) Immunostaining for human nuclei marker, human cell surface marker NCAM, and neuronal markers doublecortin and calretinin in control cells 5 weeks after transplantation into mouse hippocampus. Host mouse doublecortin and calretinin-positive cells are not colocalized with human markers. Scale bar, 100 μm. (I) Bright-field images showing representative examples of APP variant, PS1 variant, and gender-matched control HSs at DIV 100. Scale bar, 200 μm. (J) Confocal images showing representative examples of immunostaining for brain cell subtype markers GFAP, O4 and MAP2, hippocampal markers ZBTB20, PROX1, and neuronal markers TBR1, calretinin, and calbindin in APP variant, PS1 variant, and gender-matched control HSs at DIV 100. Scale bar, 50 μm. (K) Immunostaining for neuronal marker MAP2, hippocampal granule neuron marker PROX1 and pre-synaptic marker synaptophysin in control HSs at DIV 100 and 3D surface reconstruction of confocal z stacks showing PROX1^+^/synaptophysin^+^/MAP2^+^ granule neuron in control HSs. Scale bars, 100 μm (HS confocal images) and 10 μm (3D reconstruction).

Next, we developed an original protocol (Figure 1A) based on the embryoid (EB) culture methodology described by Pasca et al. (2015). To generate dorsomedial telencephalic neural precursors, the EBs were simultaneously treated with dual smad signaling chemical inhibitors LDN-193189 and SB-431542 (Chambers et al., 2009, Roybon et al., 2013). Dickkopf-specific chemical agonist XAV-939 (Nicoleau et al., 2013) and smoothen-binding chemical inhibitor of hedgehog signaling cyclopamine (Chen et al., 2002) were used to antagonize the formation of caudal and ventral tissue, specifying the cells toward a dorsal telencephalic fate (Watanabe et al., 2005). To specify the hippocampal identity, EBs were exposed to CHIR-99021, a chemical activator of WNT signaling (Lee et al., 2000), and brain-derived neurotrophic factor to allow expansion of hippocampal neural progenitors (Bull and Bartlett, 2005).

EBs aged 30 days *in vitro* (DIV) were almost exclusively composed of LEF1-positive cells (Figure 1B), suggesting that our protocol led to the formation of neural progenitors regionalized toward medial pallium tissue (Abellan et al., 2014). Quantitative analysis reveals that the EBs contained mainly neurons (Figure S2A) that were positive for microtubule-associated protein 2 (MAP2). Glial fibrillary acidic protein (GFAP)-positive astrocytes represented less than 2% of the population, and O4-positive oligodendrocytes were absent (Figures 1C and 1D). Importantly, 90% of the cells were positive for ZBTB20 and 45%–60% were positive for PROX1 (Figures 1C and 1E). The cultures also contained PAX6-, TBR1-, calretinin-, and calbindin-positive cells (Figures 1C and 1F), indicating that the hippocampal cells were at different stages of maturation (Roybon et al., 2009b). Few GABA-positive cells were identified (Figure S2B).

To validate our finding, we transplanted single-cell suspension from 50-day-old EBs of one of the iPSC control lines (CSC-37N) into the hippocampi of adult RAG-1-deficient mice and examined the graft composition 5 weeks later. The grafted cells (human nuclei-positive) co-expressed ZBTB20 and PROX1 (Figure 1G), as well as doublecortin and calretinin (Figure 1H).

When aged 100 DIV, the EBs were large in size, with no obvious alterations (Figure 1I). Although they contained some GFAP-positive astrocytes, they were primarily composed by MAP2-positive neurons (Figure 1J) co-expressing ZBTB20 and PROX1. They also contained TBR1 and an almost equal ratio of calretinin/calbindin-positive cells (Figure 1J). Detailed analysis of EBs revealed the presence of pre-synaptic synaptophysin-positive puncta at the surface of MAP2/PROX1-positive neurons (Figure 1K). We named these HSs, and further examined their relevance for modeling AD.

### APP and PS1 Variant HSs Exhibit AD-Related Pathology

**Figure 2:**
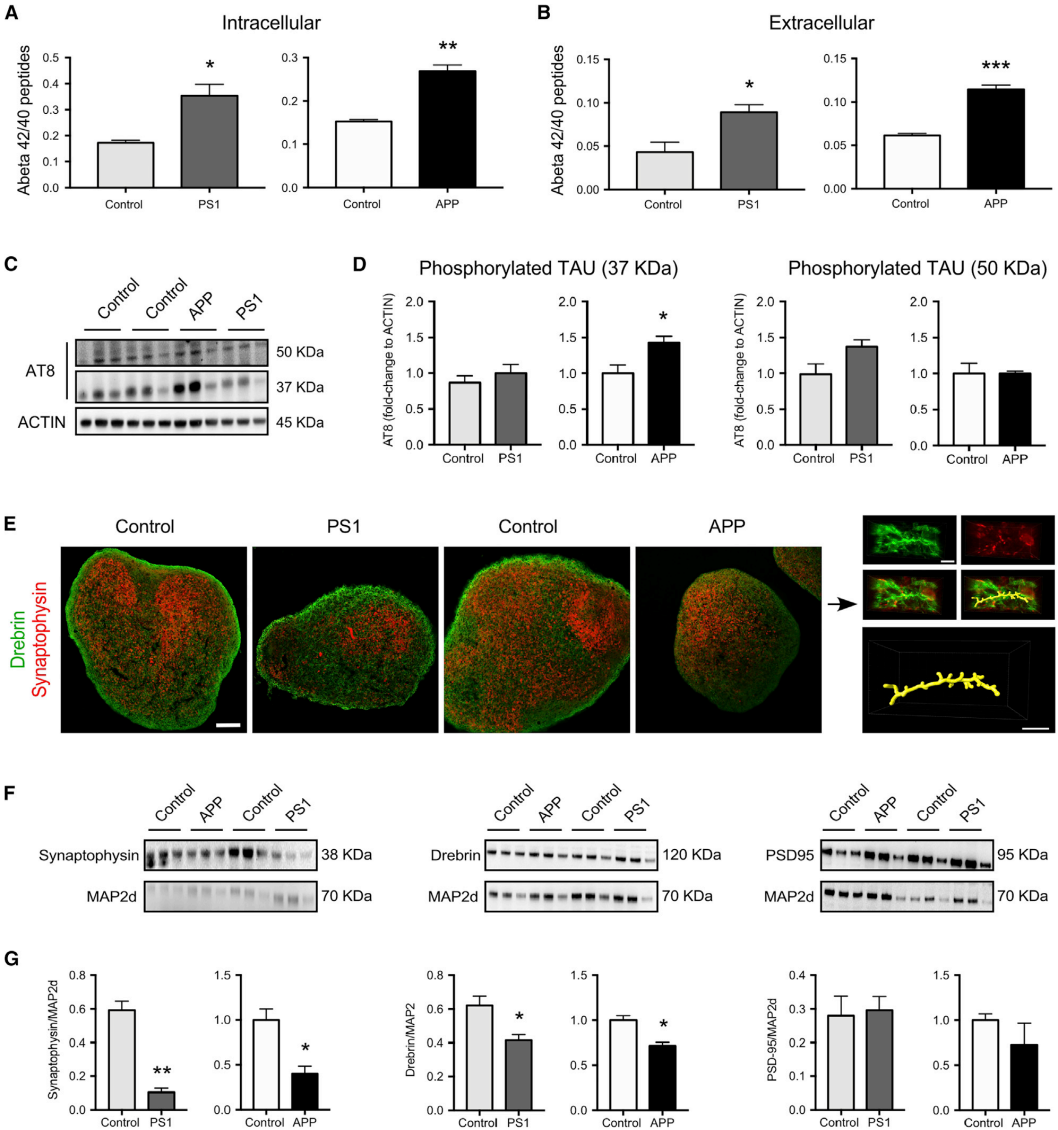
APP and PS1 Variant HSs Exhibit AD-Related Pathology (A and B) Characterization of amyloid-β (Aβ) accumulation (intracellular Aβ, a) and secretion (extracellular Aβ, b) in APP variant, PS1 variant, and gender-matched control HSs at DIV 100. The ratio of Aβ42/Aβ40 in HS lysates (A) and the ratio of Aβ42/Aβ40 secreted from HSs into the medium (B) were measured at day 4 after the last medium change. For quantitation, data were normalized to the total protein. Results are presented as mean ± SEM. n = 3 independent differentiations per genotype. ^∗^p < 0.05, ^∗∗^p < 0.01, ^∗∗∗^p < 0.001. Statistical analysis by two-tailed t test. See also Figure S2. (C and D) Characterization of phosphorylation of tau protein in APP variant, PS1 variant, and gender-matched control HSs. Western blotting analysis of phosphorylation of tau protein in HSs at DIV 100 with actin blot included as a loading control (C). The blots were quantified densitometrically and for quantitation of tau phosphorylation level, data were normalized by the level of actin (D). n = 3 independent differentiations per genotype. ^∗^p < 0.05. Statistical analysis by two-tailed t test. (E–G) Characterization of synaptic proteins in APP variant, PS1 variant, and gender-matched control HSs. Confocal images showing representative examples of immunostaining for synaptic markers synaptophysin and drebrin in APP variant, PS1 variant, and gender-matched control HSs at DIV 100 and 3D surface reconstruction of dendritic segment with putative dendritic spines as delineated by drebrin labeling in close proximity to synaptophysin puncta in control HSs (E). Western blotting analysis of synaptic markers synaptophysin, drebrin, and PSD-95 in HSs at DIV 100 with MAP2d blot included as a loading control (F). The blots were quantified densitometrically and for quantitation of synaptic protein levels, data were normalized by the level of MAP2d (G). Results are presented as mean ± SEM. n = 3 independent differentiations per genotype. ^∗^p < 0.05; ^∗∗^p < 0.01. Statistical analysis by two-tailed t test. Scale bars, 100 μm (HS confocal images) and 5 μm (3D reconstruction images).

At first, we measured the amount of extra- and intracellular Aβ40 and Aβ42 peptides present in 100 DIV HSs. Both APP and PS1 variant HSs and their culture supernatants contained Aβ peptides with a higher ratio of Aβ42/Aβ40 than control HSs (approximately 1.5-fold higher for PS1 variant and approximately 2-fold higher for APP variant; Figures 2A and 2B), which concurs with previous studies (Duering et al., 2005, Murphy and LeVine, 2010). The change in Aβ42/Aβ40 ratio was mainly due to increased levels of Aβ42 peptides (Figure S3), suggesting that the cells carrying both variations had altered metabolism. The levels of released and intracellular Aβ40 peptide were either not altered (as in PS1 variant HSs) or showed a trend toward decreased production (APP variant HSs) (Figure S2). We also examined changes in levels of Aβ38 peptide but found none (Figure S2).

We next examined the state of phosphorylation of tau, previously reported to be enhanced by oligomeric Aβ peptides (Jin et al., 2011), and which is the major protein of the neurofibrillary tangles in AD when in its hyperphosphorylated form (Mandelkow and Mandelkow, 2012). We performed western blotting, using the monoclonal antibody AT8, which recognizes phosphorylation of tau at both serine 202 and threonine 205 (Goedert et al., 1995), and detected two monomeric tau bands (at 50 and 37 kDa molecular weight) in both AD and control samples (Figures 2C and 2D). We found significantly higher levels of the 37-kDa phosphorylated tau protein isoform in APP variant HSs, as compared with gender-matched control HSs.

Since synaptic pathology is an early morphological change in the human AD postmortem brain (Forner et al., 2017, Scheff et al., 2014), we assessed several key synaptic proteins in the HSs. Immunohistochemistry showed the presence of the pre-synaptic protein synaptophysin and the post-synaptic protein drebrin (Figure 2E), suggesting that synaptic contacts are forming in HSs (Figure 2E). However, western blot analysis revealed that their abundance differed between AD and control samples, and levels of the two synaptic proteins were significantly decreased in both APP and PS1 variant HSs compared with their respective controls (Figures 2F and 2G). We also examined if there was a change in levels of the post-synaptic protein PSD-95 but found none (Figures 2F and 2G).

### APP Variant Hippocampal Neurons Exhibit Several Significant Alterations

**Figure 3:**
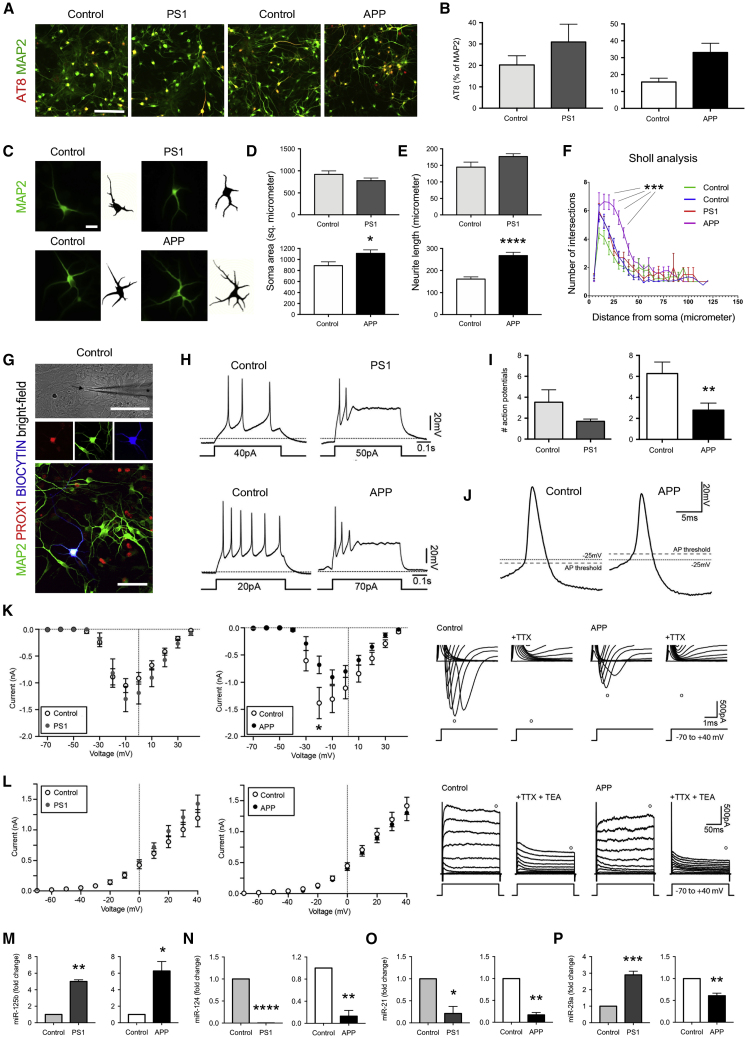
APP Variant Hippocampal Neurons Exhibit Significant Alterations (A and B) Characterization of phosphorylation of tau protein in APP variant, PS1 variant, and gender-matched control hippocampal neurons. Immunostaining for phosphorylated tau protein in hippocampal neurons at DIV 56 (A). Quantification of phosphorylated (AT8-positive) neurons expressed relative to the total number of MAP2-positive neurons in hippocampal neurons at DIV 56 (B). Results are presented as mean ± SEM. n = 1–3 independent differentiations per clone for N = 3 iPSC clones per genotype. ^∗^p < 0.05. Statistical analysis by two-tailed t test. Scale bar, 200 μm. (C–F) Representative original fluorescence and converted binary images of APP variant, PS1 variant, and gender-matched control MAP2-positive hippocampal neurons at DIV 56 (C). Soma area (D), neurite length (E), and dendritic arborization (F) were quantified. Results are presented as mean ± SEM. n = 16–23 cells per line measured from 3 independent differentiations per genotype. ^∗^p < 0.05, ^∗∗∗^p < 0.001, ^∗∗∗∗^p < 0.0001. Statistical analysis by two-tailed t test (D and E) and repeated measures ANOVA (F). Scale bar, 20 μm. (G–L) Whole-cell patch-clamp recordings from APP variant, PS1 variant, and gender-matched control hippocampal neurons at DIV 56–58. Representative bright-field image of a patched control cell (arrow) along with the patch-clamp pipette (^∗^) (G, upper panel) and fluorescence image of a patched PROX1-positive neuron filled with biocytin (G, lower panel). Voltage traces show the ability of hippocampal neurons to fire action potentials (APs) upon current injections (H). Bar diagrams show the maximal number of APs generated upon current injections (I). Expanded voltage traces of the first AP induced by a current ramp of 300 pA and used for determining the AP characteristics (J). Expanded current traces illustrating the inward sodium current (denoted by O) activated during voltage steps ranging from −70 to +40 mV in 10-mV steps (K, right panel). The sodium current was blocked by the presence of 1 μM TTX. The current-voltage plots illustrate the sodium current peak plotted against the voltage steps (K, left panel). Current traces illustrating the outward potassium current (denoted by O) activated during voltage steps ranging from −70 to +40 mV in 10-mV steps (L, right panel). The potassium current was inhibited by the addition of 10 mM TEA. The current-voltage plots illustrate the potassium current plotted against the voltage steps (L, left panel). Results are presented as mean ± SEM. n = 23–26 cells recorded from 3 independent differentiations per genotype. ^∗^p < 0.05. Statistical analysis by Mann-Whitney test (I) and multiple t tests (K and L). Scale bars, 100 μm. (M–P) Characterization of microRNA expression in APP variant, PS1 variant, and control hippocampal neurons. Bar diagrams showing the relative expression of miR-125b (M), miR-124 (N), miR-21 (O), and miR-29a (P) measured with RT-PCR in hippocampal neurons at DIV 56. Results are presented as mean ± SEM. n = 3 independent differentiations per genotype. ^∗^p < 0.05; ^∗∗^p < 0.01. Statistical analysis by two-tailed t test.

To gain insights into possible alterations of young AD hippocampal neurons, we examined phosphorylation of tau, the morphometric characteristics and electrophysiological properties of neurons, and miRNA levels of expression in cultures at 56 DIV. We found a higher proportion of AT8/MAP2-positive neurons in APP, but not in PS1 variant cultures as compared with controls (Figures 3A and 3B). Interestingly, the size of the soma, and the length and complexity of the neurites were altered only in APP variant neurons (Figures 3C–3F). Since morphometric characteristics can affect functional properties of neurons (Deshpande et al., 2017), we examined the functional differences between AD and healthy neurons using whole-cell patch-clamp recording (Figure 3G). Following somatic current injection, both AD and healthy hippocampal neurons were able to fire action potentials (APs) (Figure 3H). However, APP but not PS1 variant hippocampal neurons generated significantly fewer APs, when compared with control hippocampal neurons (Figures 3H and 3I). In addition, APs of APP variant neurons had a more depolarized threshold and lower amplitude (Figure 3G; Table S1). In line with these data, we observed that the amplitude of the TTX-sensitive inward sodium current was significantly reduced for APP but not PS1 variant hippocampal neurons when compared with controls (Figure 3K); whereas the outward, TEA-sensitive K current was unaltered for both APP and PS1 variant hippocampal neurons (Figure 3L).

Because miRNAs play an important role in AD pathogenesis (Miya Shaik et al., 2018), we next examined the levels of expression of several selected miRNAs previously identified to be dysregulated in AD models and patients. miRNA (miR)-125b was shown to be increased in the cerebrospinal fluid of AD patients (Dangla-Valls et al., 2017), and its overexpression causes tau hyperphosphorylation and impairs associative learning when injected into the hippocampus of mice (Banzhaf-Strathmann et al., 2014). We found miR-125b to be significantly upregulated in both APP and PS1 variant neural cultures (Figure 3M). Other microRNAs, such as miR-124, miR-21, and miR-29a/b were reported to be decreased in AD (An et al., 2017, Hebert et al., 2009, Leidinger et al., 2013, Lukiw, 2007, Shioya et al., 2010). Accordingly, we observed miR-124 and miR-21 downregulated in both APP and PS1 variant neuronal cultures (Figures 3N and 3O). Interestingly, miR29a, which is known to be involved in the regulation of APP and β site APP cleaving enzyme 1 (BACE1) expression (Hebert et al., 2009), was downregulated in APP variant but upregulated in PS1 variant neural cultures (Figure 3P). This is in line with the reports showing that miR-29a could be differently expressed in AD brain (Cogswell et al., 2008, Hebert et al., 2008, Shioya et al., 2010).

### APP Variant HSs Exhibit Increased Protein Aggregation

**Figure 4:**
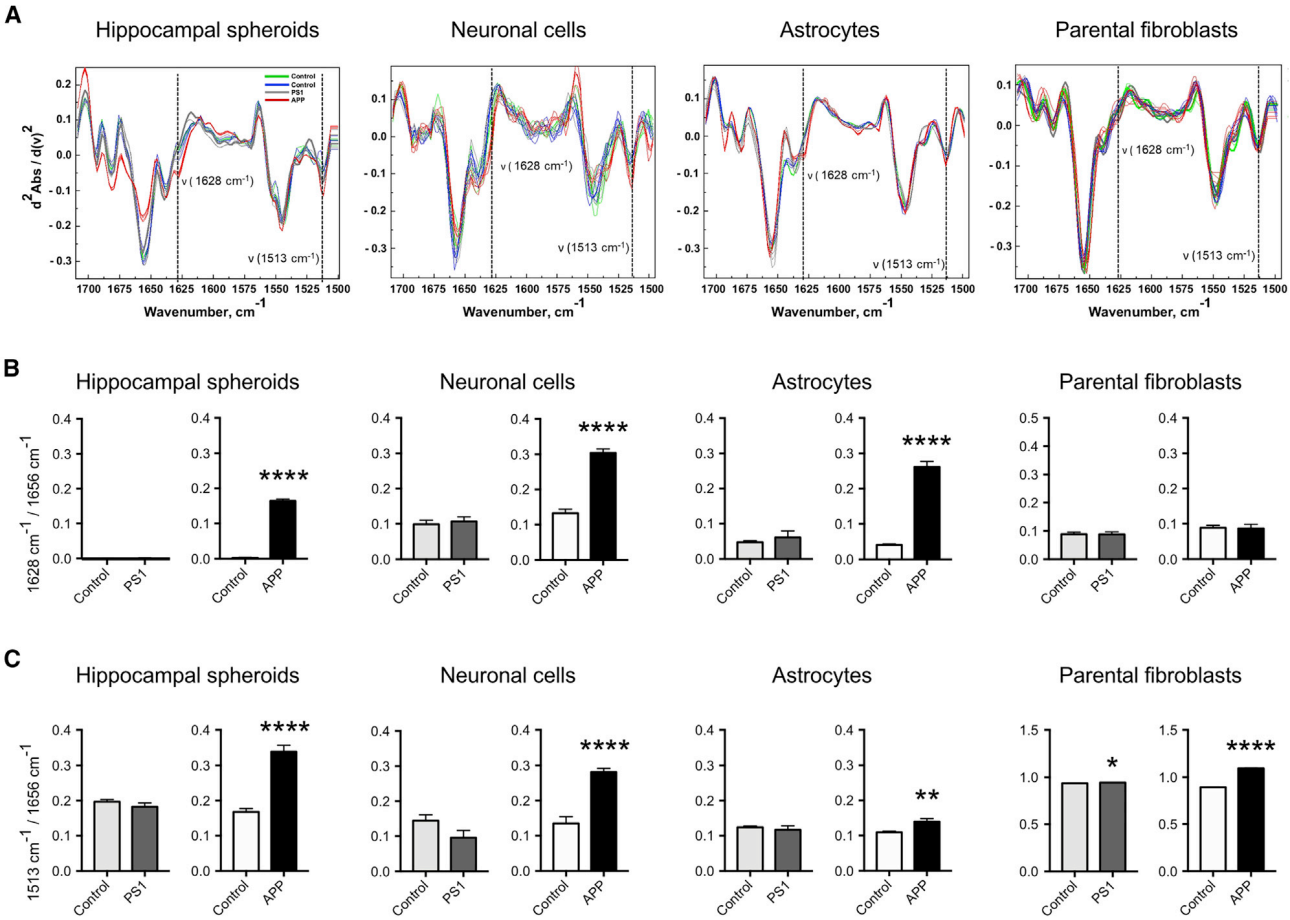
APP Variant HSs Exhibit Increased Protein Aggregation (A) Normalized second derivatives of the infrared light absorbance spectra in Amide I and II regions. Dashed lines and arrows indicate the increased protein aggregation as elevation of β sheet structures (1,628 cm^−1^) and structural changes corresponding to protein aggregation, as elevation at the band positioned at 1,513 cm^−1^ in APP in HSs at DIV 100, hippocampal neurons at DIV 56, astrocytes at DIV 120, and parental fibroblasts. (B) Bar diagrams reflecting the β sheet structure content as shown by the absorbance ratios 1,628 to 1,656 cm^−1^ in the same cells described in (A). Results are presented as mean ± SEM. n = 5–10 spectra per genotype for HSs, n = 20–26 spectra per genotype for parental fibroblasts, n = 3 independent differentiations per genotype for hippocampal neurons and astrocytes. ^∗^p < 0.05, ^∗∗^p < 0.01, ^∗∗∗∗^p < 0.0001. Statistical analysis by two-tailed t test. (C) Bar diagrams reflecting structural changes as shown by the absorbance ratios 1,513 to 1,656 cm^−1^ in same cells described in (A). Results are presented as mean ± SEM. n = 5–10 spectra per one genotype for HSs. n = 20–26 spectra per genotype for parental fibroblasts, n = 3 independent differentiations per genotype for hippocampal neurons and astrocytes. ^∗^p < 0.05, ^∗∗^p < 0.01, ^∗∗∗∗^p < 0.0001. Statistical analysis by two-tailed t test.

Both increased phosphorylation of tau in APP variant HSs and neurons (Figures 2C, 2D, 3A, and 3B) and increase in Aβ42/Aβ40 peptide ratios in both APP and PS1 variant HSs (Figure 2A) suggested that protein aggregation may be occurring in the patient hippocampal cells. To examine intracellular protein aggregation in the HSs, we used FTIR, a microspectroscopy-based imaging technique, as described previously (Klementieva et al., 2017). To better resolve the peak positions, we performed a second derivative analysis (de Aragao and Messaddeq, 2008). Strikingly, analysis of FTIR spectra revealed a significant increase in the content of β sheet structures in APP but not in PS1 variant HSs compared with controls (Figures 4A and 4B). This suggested that, although the Aβ42/Aβ40 ratio was increased in both APP and PS1 variant HSs, protein aggregation may be ongoing in the APP variant HSs only.

Since both neurons and astroglia were found in HSs, we investigated whether these two cell types could contribute to the increased protein conformational changes and aggregation in the AD variant HSs. We performed FTIR in neuronal cultures generated by dissociating HSs and culturing single cells as a monolayer until aged 56 DIV, and astroglial cultures generated by culturing HSs-derived progenitors as monolayers in medium containing ciliary neurotrophic factor until aged 100 DIV. We observed robust and significant increases in β sheet structures in APP hippocampal neurons. These data were confirmed when using a different platform and different iPSC clones (Figure S4). Interestingly, astrocytes derived from APP variant, but not from PS1 variant and control HSs (Figures 4A and 4B), displayed increases in β sheet structures. These data indicated that pathological changes also occur in AD astrocytes, as recently suggested (Oksanen et al., 2017). Importantly, we found no increase in β sheet structures in APP variant parental fibroblasts, demonstrating that protein aggregation is not present in the patient APP variant fibroblasts. This allowed us to rule out the possibility that pathological aggregation had been transferred from the APP variant fibroblasts to the iPSCs during reprogramming (Figures 4A and 4B).

Further assessment of protein aggregation by FTIR revealed elevation in the 1,513-cm^−1^ band, reflecting structural changes in tyrosine and tryptophan amino acids sensitive for phosphorylation (Barth et al., 1994), in APP, but not in PS1 variant HSs, neurons, and astrocytes (Figures 4A and 4C). Interestingly, significant elevation of the 1,513-cm^−1^ band was found in the two AD variants fibroblasts, although the level was low in PS1 variant fibroblasts (Figures 4A and 4C).

### Quantitative Proteomic Analysis Reveals Important Alterations in the Proteome of APP and PS1 Variant HSs

**Figure 5:**
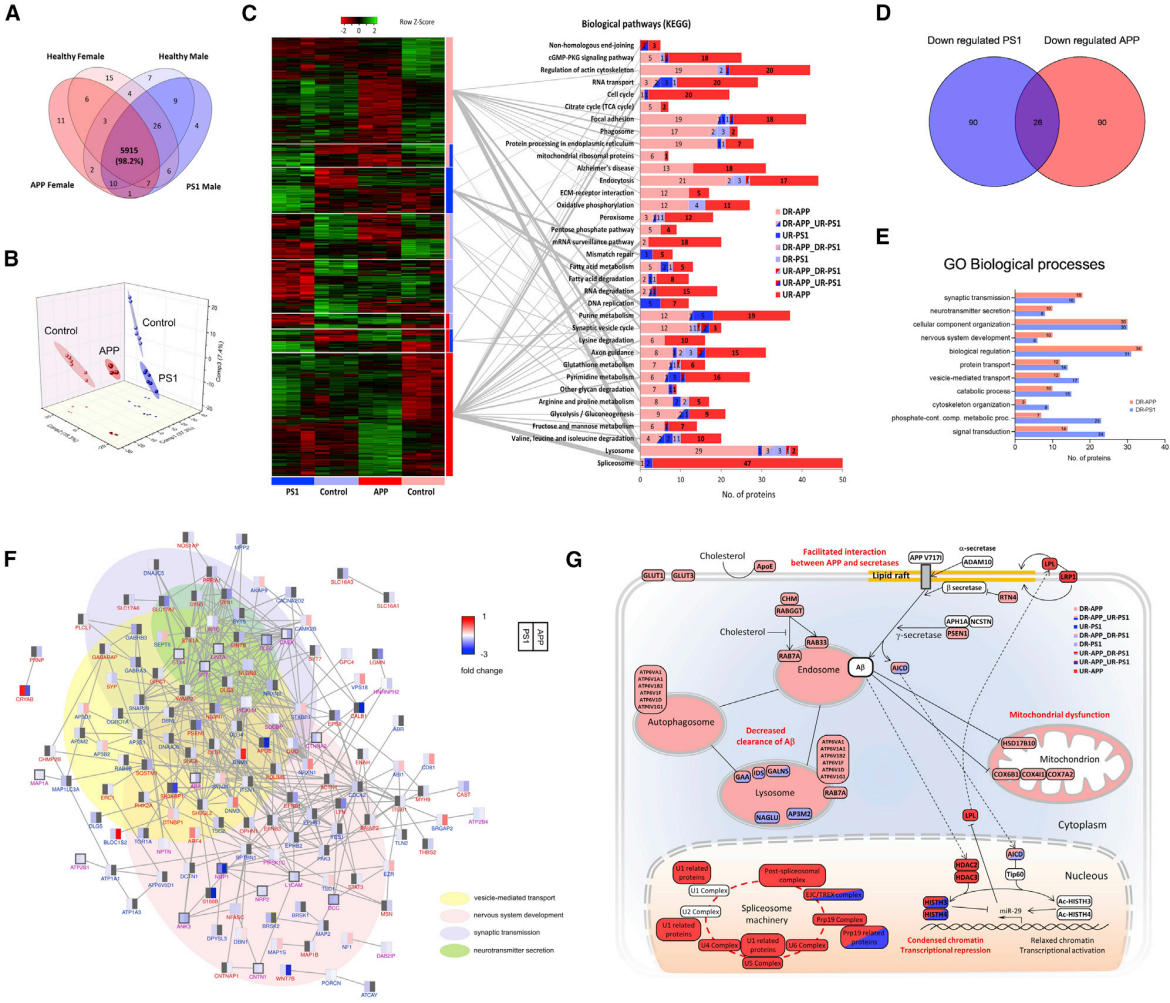
Label-free Quantitative Proteomics Reveals Important Alterations in the Proteome of APP and PS1 Variant HSs (A) Venn diagram representing the number of proteins identified in HSs. The numbers correspond to the set of three different processes of generating HSs (biological replicates) and two technical replicates of the LC-MS/MS analysis. (B) Principal component analysis using the normalized intensities of proteins identified/quantified in all samples. (C) Heatmap of the protein relative abundances that were significantly dysregulated in at least one of the variant samples (left). n = 3 independent differentiations per genotype, n = 2 LC-MS/MS analyses. False discovery rate (FDR) < 0.05 was considered significant. Statistical analysis by two-tailed t test. Biological pathways (Kyoto Encyclopedia of Genes and Genomes) enriched in at least one of the eight blocks of dysregulated proteins (right). The graph shows the distribution of the proteins involved in each biological pathway by blocks of dysregulated proteins. Gray lines connect each block of proteins with their most enriched pathways. (D) Venn diagram representing the number of synaptic proteins significantly downregulated in PS1 and APP variants when compared with their gender controls. n = 3 independent differentiations per genotype, n = 2 LC-MS/MS analyses. p < 0.05 with log2 fold change <−0.2 was considered significant. Statistical analysis by two-tailed t test. (E) Representative enriched biological processes (Panther GO-Slim) for the downregulated synaptic proteins in PS1 and APP variants are shown with the number of corresponding proteins. (F) Interaction network of the synaptic proteins downregulated in at least one of the samples carrying a mutation (n = 206). Each protein is represented by a rectangle divided into two parts (left-right), where different colors specifying the corresponding fold changes in PS1 and APP variants compared with their gender controls. Lines between the nodes (rectangles) indicate protein-protein interactions. Nodes with dark gray border represent synaptic proteins that are significantly downregulated in each variant. Purple label indicates significant downregulation in both variant, while blue and red labels indicate significant downregulation in PS1 or APP variant, respectively. n = 3 independent differentiations per genotype, n = 2 LC-MS/MS analyses. p < 0.05 with log2 fold change <−0.2 was considered significant. Statistical analysis by two-tailed t test. (G) Schematic summary of AD-related dysregulated pathways and proteins in APP and PS1 variant HSs revealed by quantitative proteomics.

To gain insights into cellular network alterations in APP and PS1 variants, we performed a quantitative assessment of the HS proteome, using label-free liquid chromatography-tandem mass spectrometry (LC-MS/MS). We identified over 6,000 proteins present in all 4 studied genotypes (Figure 5A) and could accurately quantify 98% of them (Table S2). Principal component analysis confirmed the HS segregation based on the gender (component 1) and the genotype (component 2) of the fibroblast donors (Figure 5B), hinting that gender-specific comparisons are important when analyzing disease phenotypes, as suggested previously in rodent studies (Richetin et al., 2017b). The largest sets of significantly dysregulated proteins were detected in APP variant HS (Table S2).

Subsequently, we performed pathway enrichment analysis of the identified dysregulated proteins, using the online bioinformatics tool DAVID. Notably, we found that proteins dysregulated in APP and PS1 variant HSs displayed a diversity of functions and were involved in several distinct pathways. For example, spliceosome, purine metabolism, and mRNA surveillance pathways were the highest of the significantly enriched pathways identified in APP variant HSs, where proteins were upregulated (Figure 5C). In contrast, in HSs harboring the PS1 variation, we identified DNA replication and DNA repair pathways as the most strongly enriched in upregulated proteins (Figure 5C). Similarly, we identified heterogeneity between the two AD variants when examining pathways enriched in downregulated proteins. Thus, in APP variant HSs, lysosomes, and phagosomes, endocytosis, oxidative phosphorylation, and protein processing in endoplasmic reticulum were the highly enriched pathways, among others, in downregulated proteins, whereas, in PS1 variant HSs, oxidative phosphorylation, endocytosis, axon guidance, lysosome and phagosome were the most strongly enriched pathways in downregulated proteins (Figure 5C).

Since we found decreased levels of synaptic proteins synaptophysin and drebrin by western blot (Figure 2F), we further examined synaptoproteomic alterations. Of all synaptic-related proteins analyzed, 26 were commonly downregulated in both APP and PS1 variant HSs (Figure 5D). Among them, we identified LIN7 proteins, which are important mediators of synaptic vesicle exocytosis (Butz et al., 1998). Interestingly, an equal number of 90 different synaptic proteins were found specifically downregulated in APP or PS1 variant HSs. Gene ontology enrichment analysis revealed that these proteins were associated with biological processes centered around synaptic function, and included synaptic transmission, neurotransmitter secretion, and vesicle-mediated transport (Figures 5E and 5F; Table S3).

Finally, quantitative LC-MS/MS revealed alterations in several AD-related pathways (Figure 5G). Notably, specifically in APP variant HSs, we observed downregulation of mitochondrial proteins involved in oxidative phosphorylation, particularly complex IV and 3-hydroxyacyl-CoA dehydrogenase (HSD17B10), supporting the hypothesis of mitochondrial dysfunction in AD (He et al., 2018). We found a decrease in the levels of APOE, the major genetic risk factor for developing late-onset AD (Kim et al., 2009), in the APP variant HSs. Other groups of proteins were found downregulated in both variant samples, although this was more profound with the APP variant, e.g., proteins responsible for clearance of Aβ peptide. For example, the lysosome and the phagosome were the most enriched pathways among the downregulated ones in the samples carrying the APP variation. Among the most upregulated proteins, the components of the spliceosome machinery, including almost all the complexes in the pathway, were upregulated in both APP and PS1 variant samples. Interestingly, two proteins belonging to the histone deacetylase class 1 group (HDAC2 and HDAC3), which are known as transcriptional repressors (Yamakawa et al., 2017), were upregulated only in APP variant HSs. Similarly, the proteins lipoprotein lipase and low-density lipoprotein receptor-related protein 1, which were previously found to be overexpressed in AD (Baum et al., 1999), were upregulated in the APP variant HS sample.

### ND1 *Ex Vivo* Gene Therapy Modulates the Transcription of Genes Relevant to AD Pathogenesis

We previously demonstrated that viral delivery of single transcription factor ND1 is sufficient to increase the maturation of rodent neural progenitors into neurons (Roybon et al., 2009a, Roybon et al., 2009b, Roybon et al., 2015), and reverse synaptic dysfunction and restore spatial memory in a rodent model of AD (Richetin et al., 2015, Richetin et al., 2017a). To evaluate the potential of human HSs to serve as a translational platform for the development and/or testing of therapies for AD, we examined whether viral delivery of ND1 was sufficient to modulate the expression of genes differentially expressed in AD patient HS neurons compared with control.

**Figure 6:**
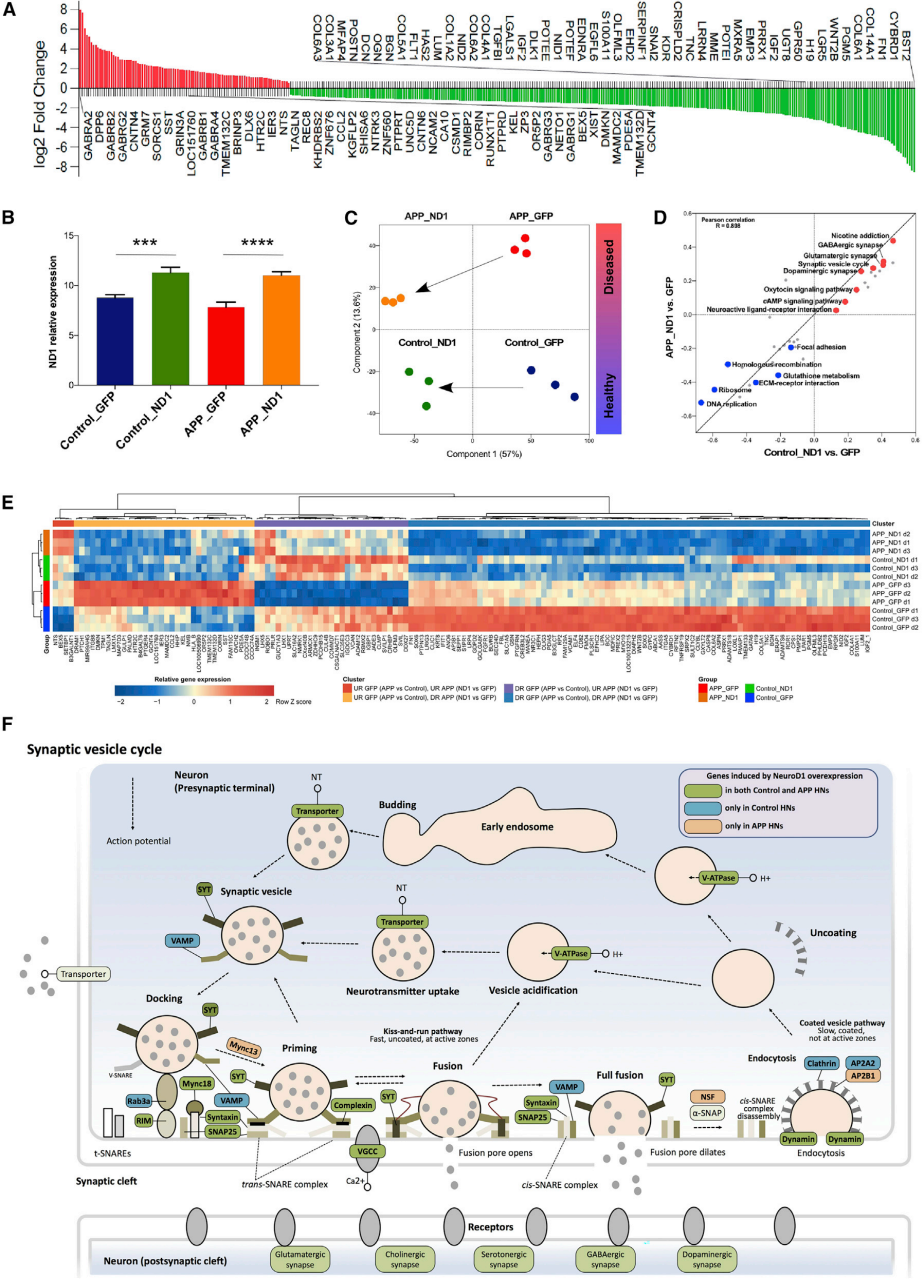
ND1 *Ex Vivo* Gene Therapy Modulates Transcription of Genes Relevant to AD (A) Genes dysregulated in APP variant hippocampal neurons at DIV 50. The top 50 genes upregulated (in red) and downregulated (in green) are annotated. n = 3 independent differentiations per genotype. FDR < 0.05 was considered significant. Statistical analysis by two-tailed t test. (B) Relative expression levels of ND1 gene. Results are presented as mean ± S.D. n = 3 independent differentiations per genotype. ^∗∗∗^p < 0.001, ^∗∗∗∗^p < 0.0001. Statistical analysis by one-way ANOVA followed by Tukey's *post hoc* test. (C) Principal component analysis using gene expression profiles of hippocampal neurons. (D) 2D annotation enrichment analysis for biological pathways based on the differential expression profiles of transcripts between hippocampal neurons transduced with ND1 and GFP, in both control (x axis) and APP variant (y axis). Pathways significantly enriched after ND1 overexpression are highlighted, downregulated (blue) and upregulated (red). n = 3 independent differentiations per genotype. Enriched pathways were selected according to a Benjamini-Hochberg FDR of 0.01 (Table S5). (E) Heatmap of the relative expression of genes that were significantly dysregulated in APP variant hippocampal neurons and modulated by ND1 overexpression (Table S5). n = 3 independent differentiations per genotype. Statistical analysis by two-tailed t test. (F) Schematic representation of synaptic vesicle cycle pathway in hippocampal neurons. Genes whose expression was induced by ND1 overexpression are highlighted.

We used Affymetrix GeneChip microarray to examine early transcriptomic changes in APP variant cells compared with control, upon viral delivery of ND1. We detected 377 genes whose expression was changed in APP variant cells compared with control (Figure 6A; Table S4). While the majority of genes (282), products of which participate in various biological processes, were downregulated in purified APP variant GFP-positive cells compared with control GFP-positive cells, 95 genes were upregulated, including genes encoding various GABA receptors, expression of which is altered in the early stages of AD (Saura et al., 2015).

Following transduction, ND1 expression was robustly identified (Figure 6B). Importantly, principal component analysis showed that ND1 overexpression changed the transcriptomic profile of APP variant GFP-positive cells, from a diseased state to an almost healthy control one (Figure 6C). In particular, ND1 positively modulated the expression of genes involved in synaptic transmission (Figures 6D–6F; and Table S5).

## Discussion

Here, we report an innovative method to chemically induce HSs from human iPSCs, which we used in a proof-of-concept study to model two patients carrying typical and atypical AD-related gene variations. We show that exposure of human iPSCs from different genders (male or female) and molecular diagnosis (diseased or healthy) to chemical agents LDN-193189, SB-431542, XAV-939, cyclopamine, and CHIR-99021 is sufficient to generate HSs containing LEF1-positive medial pallium progenitors, which when matured further formed hippocampal neural cells at different stages of maturation. While this original protocol produces approximately the same percentage of cells positive for ZBTB20 and PROX1, as recently reported by Sakaguchi et al. (2015) using human embryonic stem cells, it is more rapid and does not require long-term attachment culture. It also does not require iPSC-derived hippocampal neural progenitors to be plated on a monolayer of primary hippocampal astrocytes to generate mature neurons (Yu et al., 2014). Although HSs contain functional neurons, they were almost devoid of astrocytes. This is a significant drawback as astrocytes recently emerged as a new important target of AD (Jones et al., 2017, Konttinen et al., 2019b, Oksanen et al., 2017). Hence, to examine protein aggregation in astrocytes, we had to modify our protocol to generate astrocytic monolayer cultures. Since during human development astrocytes are generated later than neurons, we may assume that the number of astrocytes in the HSs may increase over time. With the constant improvement of protocols aiming at generating oligodendrocytes from human iPSCs (Djelloul et al., 2015, Madhavan et al., 2018), it would be valuable to also examine AD pathology in this cell type in future studies, since white matter myelin loss occurs in the AD patient brain (Mitew et al., 2010, Nasrabady et al., 2018). Cellular pathology was recently examined in iPSC-derived microglia (Konttinen et al., 2019a), a cell type that plays an important role in AD pathology (Venegas et al., 2017).

We utilized 3D hippocampal structures generated from iPSCs, to examine cellular dysfunction in typical and atypical familial AD. We used several techniques, from routine (western blotting, immunocytochemistry, qRT-PCR, MSD multi-array, and Affymetrix GeneChip microarray) to more sophisticated (whole-cell patch-clamp recording, synchrotron-based FTIR, and quantitative LC-MS/MS) and identified a wide variety of disease phenotypes. Some of them were reported previously when analyzing postmortem tissue, e.g., increased ratio of Aβ42/Aβ40 peptides, which can be neurotoxic (Kuperstein et al., 2010), and decreased levels of synaptic proteins levels (de Wilde et al., 2016), which are well-known cellular hallmarks of AD. Importantly, we identified cellular alterations that varied between the two AD variants, as also reported by others (Israel et al., 2012, Kondo et al., 2013). For example, we provide evidence of a significant increase in the content of β sheet structures and in the level of tau phosphorylation in APP but not in PS1 variant HSs compared with the controls. The difference of disease phenotypes, which was more prominent, or speculatively more advanced, in the APP variant when compared with the PS1 variant, could potentially be attributed to the fact that this patient carried a homozygous variation p.V717I in the APP gene (Sorbi et al., 1993), which could promote a stronger cellular pathology than if it was in a heterozygous form (Kondo et al., 2013, Ovchinnikov et al., 2018, Woodruff et al., 2016). Alternatively, it is possible that the PS1 variant displayed milder cellular phenotypes due to the nature of the variation. Indeed, patients carrying this PS1 p.R278K variation show a wide clinical spectrum, even between family members, from classical AD to pure spastic paraparesis (Assini et al., 2003). In this particular case, spastic paraparesis was the initial feature, which we speculate might explain the lesser phenotypic changes (e.g., protein aggregation) in HSs from the PS1 compared with APP variants. The correlation between patients' symptoms and cellular pathogenesis was demonstrated by several groups, including Woodard et al. (2014) who studied iPSC-derived dopaminergic neurons generated from GBA mutant Parkinsonian monozygotic twins clinically discordant in their symptoms: the iPSC-derived dopaminergic neurons generated from the symptomatic twin exhibited strong cellular pathogenesis, as opposed to those generated from the asymptomatic brother. In addition, the wide variety and heterogeneity of the phenotypes identified could be influenced by factors that exacerbate or alleviate them.

One limitation of this study is the number of patients used. This is due to the uniqueness of the variations they carry: homozygous APP London variation is rare (Sorbi et al., 1993); and PS1 p.R278K variation was only reported in one family (Assini et al., 2003). To minimize this limitation, we used several independent iPSC clones per individual, to examine the efficacy of our protocol and the main readout assays (tau phosphorylation and protein aggregation). Nevertheless, HSs allowed us to identify early disease phenotypes consistent across the different iPSC clones studied, which is not possible with current imaging methods, such as positron electron tomography or magnetic resonance imaging.

Our work also raises the question whether AD is a neurodevelopmental disorder? AD is considered, such as Parkinson disease and several other common neurodegenerative disorders, as a disease of the aging brain (Wyss-Coray, 2016). Early disease cellular phenotypes can be identified using pre- and post-natal rodent brain cells (Almeida et al., 2005, Nagai et al., 2007), while disease onset declares itself only after several months in the rodent models; and early changes can be observed when using advanced techniques, such as FTIR (Klementieva et al., 2017). This raises even more attention when modeling diseases using iPSCs. Indeed, even if iPSCs are non-natural cell types generated by the reprogramming of (often) aged somatic cells (Dimos et al., 2008), they are embryonic-like in their nature (Ho et al., 2016, Zhao et al., 2018). Over the last decade, experimental work using iPSCs suggests that early disease phenotypes can be identified as soon as a few weeks to few months *in vitro* for several neurodegenerative disorders (Fujimori et al., 2018, Guo et al., 2017, Muratore et al., 2014, Woodard et al., 2014). Moreover, recent work suggested that early therapeutic intervention could alleviate early behavioral, cellular, and molecular changes seen in Huntington disease (HD) (Siebzehnrubl et al., 2018), and disease phenotypes have been reversed in neuronal cells generated from iPSCs of HD patients (Consortium, 2017). This raises the possibility that early pharmacological intervention in AD (and other neurodegenerative disorders) might also be valuable. Toward this aim, we evaluated the effect of ND1 on human HSs-derived neuronal progenitors. This strategy was previously used with *in vitro* and *in vivo* systems, and it proved to be beneficial in rodent models of AD by reversing memory impairment (Richetin et al., 2015, Richetin et al., 2017a). We found that ND1 positively modulated the expression of genes involved in synaptic transmission (Figures 6D–6F). This is important since synaptic transmission impairment is a hallmark of AD pathology (de Wilde et al., 2016), and ND1 overexpression is sufficient to stimulate synaptic connectivity and excitability of new adult hippocampal neurons (Richetin et al., 2015, Richetin et al., 2017a). This proof-of-concept study further showed that HSs could serve as a platform for developing therapeutic strategies and evaluate their mechanism of action at the cellular level.

In conclusion, iPSC-derived HSs provide a complementary technology to 2D human hippocampal monolayers developed recently (Sarkar et al., 2018, Yu et al., 2014), and offer the possibility to examine early pathological changes in a minimalist human AD hippocampal parenchyma-like model, although devoid of oligodendrocytes, microglia, and vasculature. HSs can further be grafted into immunodeficient mice, either as single cells, as we did, or as a whole (Mansour et al., 2018), which can further offer an invaluable tool to examine how cellular pathology develops, as well as the effects of potential drugs on human diseased cells, *in vivo*, over time.

## Experimental Procedures

All reagents and procedures details can be found in Supplemental Experimental Procedures.

### Generation of iPSCs

Primary human dermal fibroblasts were harvested by punch skin biopsy from AD patients and healthy donors after written informed consent. Reprogramming factors (OCT-3/4, KLF-4, SOX-2, and c-MYC) were delivered using a non-integrating Sendai virus vectors kit (Thermo Fisher Scientific). A month later, several colonies were collected and expanded as single clones for 7 days. Two to three iPSC clones per individuals were selected for further expansion and characterization (Figure S1). The reprogramming of patient cells into iPSC was approved by the Swedish work environment authority. An additional female control line (TALSCTRL15.12) was received from Target ALS (http://www.targetals.org/) and NHCDR Repositories hosted by RUCDR. Work was carried out according to European and Swedish national rules, with the highest level of ethics.

### Differentiation of iPSCs to HSs

Human iPSC colonies were dissociated using dispase and transferred into ultra-low adherent flasks (Corning) in WiCell medium supplemented with 20 ng/mL fibroblast growth factor 2 and 20 μM ROCK inhibitor Y-27632 (Selleck Chemicals, Munich, Germany). Next day, WiCell was replaced with neural induction medium composed of advanced DMEM/F12, 2% B27 Supplement without vitamin A (v/v), 1% N2 Supplement (v/v), 1% NEAA (v/v), 2 mM L-glutamine and 1% penicillin-streptomycin (v/v). For dorsomedial telencephalic neural specification, LDN-193189 (Stemgent, 0.1 μM), Cyclopamine (Selleck Chemicals, 1 μM), SB431542 (Sigma-Aldrich, 10 μM) and XAV-939 (Tocris, 5 μM) were added to the medium for the first 10 days with medium change every other day. On the 10th day, the free-floating spheres were transferred to neuronal differentiation medium (NDM) containing Neurobasal medium, 1% N2 (v/v), 1% NEAA (v/v), L-glutamine, and 1% penicillin-streptomycin (v/v). To promote hippocampal differentiation, NDM was supplemented with CHIR-99021 (Stemgent, 0.5 mM) and brain-derived neurotrophic factor (PeproTech, 20 ng/mL) for 90 days with medium changes every second day.

### Data and Code Availability

The mass spectrometry proteomics data have been deposited to the ProteomeXchange Consortium via the PRIDE partner repository with the dataset identifier PXD012524. Affymetrix GeneChip microarray data are available in Table S4 and contain main information on the probes; the full dataset has been deposited to the Gene Expression Omnibus repository with the dataset identifier GSE149599. Omics data are also available at the Roybon laboratory website (https://www.ipsc-cns-disease.lu.se/ipsc-laboratory-for-cns-disease-modeling/resources), or via simple email request.

## Author Contributions

Y.P. and L.R. conceived the experiments and wrote the manuscript; all authors performed or assisted with the experiments; and provided reagents, expertise, and conducted a critical analysis and review of the manuscript. All authors provided input during the editing of the manuscript and approved its content.

## Declaration of Interests

The authors declare no competing financial interests.

## Supplemental Information

Document S1. Supplemental Experimental Procedures, Figures S1–S4, and Tables S1 and S6–S8

Table S2. Summary of the Quantitative Proteomic Data

Table S3. Summary of the Quantitative Proteomic Data—A Subset of Synaptic-Related Proteins

Table S4. Relative Expression of Genes in Control and APP Variant Hippocampal Neurons after GFP or ND1 Gene Transfer

Table S5. Relative Expression of Annotated Genes Dysregulated in Control and APP Variant Hippocampal Neurons upon ND1 Gene Delivery

Document S2. Article plus Supplemental Information
