# Human iPSC-derived hippocampal spheroids: An innovative tool for stratifying Alzheimer disease patient-specific cellular phenotypes and developing therapies

**DOI:** 10.1016/j.stemcr.2021.10.003

**Published:** 2021-11-09

**Authors:** Yuriy Pomeshchik, Oxana Klementieva, Jeovanis Gil, Isak Martinsson, Marita Grønning Hansen, Tessa de Vries, Anna Sancho-Balsells, Kaspar Russ, Ekaterina Savchenko, Anna Collin, Ana Rita Vaz, Silvia Bagnoli, Benedetta Nacmias, Claire Rampon, Sandro Sorbi, Dora Brites, György Marko-Varga, Zaal Kokaia, Melinda Rezeli, Gunnar K. Gouras, Laurent Roybon

## Main text

(Stem Cell Reports *15,* 256–273, July 14, 2020)

During editing of the manuscript, the symbol “μ” was mistakenly converted to “m” in three places. This led to unintentional changes in measurement units, converting “μM” to “mM” for CHIR-99021 in one place in the main text and two places in the supplemental experimental procedures.

The authors would like to thank their colleagues for identifying this error and apologize for any inconvenience caused. These instances in the manuscript online and in the supplemental information have now been corrected. The text that reads differently is provided below.

(1) Page 269 of the main text. In the “Experimental Procedures” section, in the subsection “Differentiation of iPSCs to HSs,” the text “To promote hippocampal differentiation, NDM was supplemented with CHIR-99021 (Stemgent, 0.5 mM)…” now reads, “To promote hippocampal differentiation, NDM was supplemented with CHIR-99021 (Stemgent, 0.5 μM)…”
(2) Page 4 of the supplemental information. In the section “SUPPLEMENTAL EXPERIMENTAL PROCEDURES,” in the subsection “Differentiation of iPSCs to hippocampal spheroids,” the text “To promote hippocampal differentiation, NDM was supplemented with CHIR-99021 (Stemgent, 0.5 mM)…” now reads, “To promote hippocampal differentiation, NDM was supplemented with CHIR-99021 (Stemgent, 0.5 μM)…”
(3) Page 4 of the supplemental information. In the section “SUPPLEMENTAL EXPERIMENTAL PROCEDURES,” in the subsection “Differentiation of iPSCs to hippocampal spheroids,” the text “Adherent cells were grown for 6 days in NDM supplemented with CHIR-99021 (0.5 mM)…” now reads, “Adherent cells were grown for 6 days in NDM supplemented with CHIR-99021 (0.5 μM)…”

