# Human iPSC-Derived Hippocampal Spheroids: An Innovative Tool for Stratifying Alzheimer Disease Patient-Specific Cellular Phenotypes and Developing Therapies

**DOI:** 10.1016/j.stemcr.2023.04.009

**Published:** 2023-05-09

**Authors:** Yuriy Pomeshchik, Oxana Klementieva, Jeovanis Gil, Isak Martinsson, Marita Grønning Hansen, Tessa de Vries, Anna Sancho-Balsells, Kaspar Russ, Ekaterina Savchenko, Anna Collin, Ana Rita Vaz, Silvia Bagnoli, Benedetta Nacmias, Claire Rampon, Sandro Sorbi, Dora Brites, György Marko-Varga, Zaal Kokaia, Melinda Rezeli, Gunnar K. Gouras, Laurent Roybon

## Main text

(Stem Cell Reports *15*, 256–273; July 14, 2020)

During the preparation of a follow-up manuscript, the authors identified a sequencing error for one of the iPSC lines originally published in this study. New sequencing data of the iPSC line clearly indicates heterozygosity of the APP patient cells. The authors would like to apologize for not having identified this error earlier and any inconvenience caused. The error does not invalidate any of the data and conclusions previously reported. The text that requires emendation in light of this sequencing error is provided below.

1. In the “Results” section of our originally published article, in the subsection, “Generation and characterization of HSs from iPSC of AD Patients and Healthy Individuals,” the text, “The female patient carried a homozygous variation in the APP gene (APP p.V717I)…” should have read, “The female patient carried a heterozygous variation in the APP gene (APP p.V717I)…”
2. In the “Discussion” section of our originally published article, the sentence, “The difference of disease phenotypes, which was more prominent, or speculatively more advanced, in the APP variant when compared with the PS1 variant, could potentially be attributed to the fact that this patient carried a homozygous variation p.V717I in the APP gene (Sorbi et al., 1993), which could promote a stronger cellular pathology than if it was in a heterozygous form (Kondo et al., 2013, Ovchinnikov et al., 2018, Woodruff et al., 2016),” should have read, “The difference of disease phenotypes, which was more prominent, or speculatively more advanced, in the APP variant when compared with the PS1 variant, could potentially be attributed to the fact that this patient carried a heterozygous variation p.V717I in the APP gene (Sorbi et al., 1993), which could promote a stronger cellular pathology (Kondo et al., 2013, Ovchinnikov et al., 2018, Woodruff et al., 2016).”
3. In the “Discussion” section of our originally published article, the sentence, “This is due to the uniqueness of the variations they carry: homozygous APP London variation is rare (Sorbi et al., 1993); and PS1 p.R278K variation was only reported in one family (Assini et al., 2003),” should have read, “This is due to the uniqueness of the variations they carry: heterozygous APP London variation is rare (Sorbi et al., 1993); and PS1 p.R278K variation was only reported in one family (Assini et al., 2003).”
4. In the supplemental information that accompanied our originally published article, the legend for Figure S1E, “e) Sequencing confirming the heterozygous p.R278K PS1 and homozygous p.V717I APP variation in patient-derived iPSCs,” should have read, “e) Sequencing confirming the heterozygous p.R278K PS1 and heterozygous p.V717I APP variation in patient-derived iPSCs.”
5. In Figure S1E in our originally published article, the chromatin confirming the p.V717I APP variation was not heterozygous, but in the corrected Figure S1E below, it is.

**Figure:**
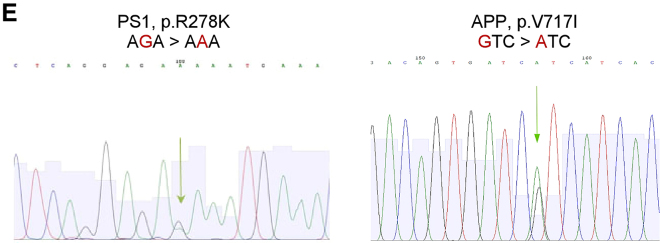
Figure S1E. Generation and characterization of iPSCs from AD patients and healthy individuals, Related to Figure 1

