# Supplementary material for: Human iPSC-Derived Hippocampal Spheroids: An Innovative Tool for Stratifying Alzheimer Disease Patient-Specific Cellular Phenotypes and Developing Therapies
Source: Stem Cell Reports. 2020 Jun 25;15(1):256–73. doi: 10.1016/j.stemcr.2020.06.001 (PMC7363942; doi:10.1016/j.stemcr.2020.06.001)

Stem Cell Reports, Volume *15*

Supplemental Information

Human iPSC-Derived Hippocampal Spheroids: An Innovative Tool for Stratifying Alzheimer Disease Patient-Specific Cellular Phenotypes and Developing Therapies

Yuriy Pomeshchik, Oxana Klementieva, Jeovanis Gil, Isak Martinsson, Marita Grønning Hansen, Tessa de Vries, Anna Sancho-Balsells, Kaspar Russ, Ekaterina Savchenko, Anna Collin, Ana Rita Vaz, Silvia Bagnoli, Benedetta Nacmias, Claire Rampon, Sandro Sorbi, Dora Brites, György Marko-Varga, Zaal Kokaia, Melinda Rezeli, Gunnar K. Gouras, and Laurent Roybon

SUPPLEMENTAL INFORMATION DIRECTORY

Human iPSC-derived hippocampal spheroids: an innovative tool for modeling typical and atypical familial Alzheimer's disease and developing therapies

Yuriy Pomeshchik, Oxana Klementieva, Jeovanis Gil, Isak Martinsson, Marita Grønning Hansen, Tessa de Vries, Anna Sancho Balsells, Kaspar Russ, Ekaterina Savchenko, Anna Collin, Ana Rita Vaz, Silvia Bagnoli, Benedetta Nacmias, Claire Rampon, Sandro Sorbi, Dora Brites, György Marko-Varga, Zaal Kokaia, Melinda Rezeli, Gunnar K. Gouras, Laurent Roybon

Pomeshchik et al. Supplementary Fig.1: **Generation and characterization of iPSCs from AD patients and healthy individuals** - related to Fig. 1
a) Summary of the iPSC lines used in this study.
b) Bright-field images showing representative colonies of APP variant, PS1 variant and gender-matched control iPSCs. Scale bars: 100 µm.
c) Immunostaining for Sendai virus, pluripotent markers OCT4, NANOG, TRA1-81, markers of the three germ layers alpha-fetoprotein (AFP) (endoderm), smooth muscle antibody (SMA) (mesoderm), and beta III-tubulin (B-III-TUB) (ectoderm) in APP variant, PS1 variant and gender-matched control iPSC colonies. Nuclei are counterstained with 4',6-diamidino-2-2phenylindole (DAPI). iPSC colonies stain positive for alkaline phosphatase activity and form EBs. Scale bars: 100 µm.
d) Karyogram of APP variant, PS1 variant and gender-matched control iPSC lines stained using Giemsa (G-banding).
e) Sequencing confirming the heterozygeous p.R278K PS1 and homozygous p.V717I APP variation in patient-derived iPSCs.
f) STR allele analysis for PS1 and APP variant iPSC lines showing identity to parental fibroblasts.

Pomeshchik et al. Supplementary Fig. 2: **Bright-field images of iPSC cultures and GABA expression in neuronal cultures aged 56 DIV** – related to Fig. 1
a) Bright-field images showing the morphology of APP variant, PS1 variant and gender-matched control cultures aged 56 DIV. Scale bars: 100 µm.
b) Percentage of GABA-positive neurons in APP variant, PS1 variant and gender-matched control cultures aged 56 DIV. Results are presented as mean ± S.E.M. n = 1-3 independent differentiations per clone for N = 3 iPSC clones per genotype. Statistical analysis by two-tailed t-test.

Pomeshchik et al. Supplementary Fig. 3: **Accumulation and secretion of amyloid-β (Aβ) peptides in HSs** – related to Fig. 2
a) Characterization of Aβ accumulation (intracellular Aβ) in APP variant, PS1 variant and gender-matched control HSs at DIV 100. Aβ38, Aβ40 and Aβ42 in HS lysates were measured at day 4 after the last medium change. For quantitation, data were normalized to the total protein. Results are presented as mean ± S.E.M. n = 3 independent differentiations per genotype. Statistical analysis by two-tailed t-test.
b) Characterization of Aβ secretion (extracellular Aβ) in APP variant, PS1 variant and gender-matched control HSs at DIV 100. Aβ38, Aβ40 and Aβ42 secreted from HSs into the medium were measured at day 4 after the last medium change. For quantitation, data were normalized to the total protein. Results are presented as mean ± S.E.M. n = 3 independent differentiations

per genotype. P values: * = P<0.05; ** = P<0.01; *** = P<0.001. Statistical analysis by two-tailed t-test.

Pomeshchik et al. Supplementary Fig. 4: **APP variant HSs exhibit increased protein aggregation.** – related to Fig. 4
Bar diagrams reflecting the β-sheet structure content, as shown by the absorbance ratios 1628 cm^{-1} to 1656 cm^{-1} for APP variant, PS1 variant and gender-matched control hippocampal neurons at DIV56. Results are presented as mean ± S.E.M. n = 2-3 iPSC clones per genotype. P values: * = P<0.05. Statistical analysis by two-tailed t-test.

Pomeshchik et al. Supplementary table 1. **Electrophysiological characteristics of human iPSC-derived hippocampal neurons**

Pomeshchik et al. Supplementary table 2. **Summary of the quantitative proteomic data**

Pomeshchik et al. Supplementary table 3. **Summary of the quantitative proteomic data – a subset of synaptic related proteins**

Pomeshchik et al. Supplementary table 4. **Relative expression of genes in control and APP variant hippocampal neurons after GFP or ND1 gene transfer.**

Pomeshchik et al. Supplementary table 5. **Relative expression of annotated genes dysregulated in control and APP variant hippocampal neurons upon ND1 gene delivery.**

Pomeshchik et al. Supplementary table 6. **Primary and secondary antibodies used for immunocyto- and immunohistochemistry**

Pomeshchik et al. Supplementary table 7. **List of primer sequences used in qRT-PCR for microRNA expression**

Pomeshchik et al. Supplementary table 8. **Primary and secondary antibodies used for Western Blotting**

SUPPLEMENTAL EXPERIMENTAL PROCEDURES

All materials were purchased from ThermoFisher Scientific, unless specified.

Generation of iPSCs
Primary human dermal fibroblasts were harvested by punch skin biopsy from AD patients and healthy donors after written informed consent. Fibroblasts harboring PS1 mutation were obtained from the Galliera Genetic Bank; the fibroblasts harboring APP mutation were provided by Prof. Sorbi. Fibroblasts were cultured and expanded in Dulbecco's modified eagle's medium (DMEM) supplemented with 10% fetal bovine serum and 1% Penicillin-Streptomycin (P/S; v/v). Cells were passaged with 0.05% trypsin, cryopreserved in liquid nitrogen and stored at -150 °C. For reprogramming, fibroblasts were seeded on 0.1% gelatin-coated 12-well plate (75.000 cells/well) and two days later transduced using the three vector preparations (MOI=5, 5, 3) included in the CytoTune™-iPS 2.0 Sendai Reprogramming Kit. Reprogramming factors (OCT-3/4, KLF-4, SOX-2 and c-MYC) were delivered by using non-integrating Sendai virus vectors from the kit. The medium was replaced daily for 7 days, after which the cells were re-

seeded onto irradiated mouse embryonic fibroblast feeder cells (CF-1 MEF, GlobalStem). From the day 8 and until day 28, the cells were cultured in WiCell medium composed of advanced DMEM/F12, 20% Knock-Out Serum Replacement (v/v), 1% nonessential amino acids (NEAA; v/v), 2 mM L-glutamine, and 0.1mM β-mercaptoethanol (Sigma-Aldrich) supplemented with 20 ng/ml FGF2. At day 28, several colonies were collected and expanded as single clones for 7 days. Two to three iPSC clones per individuals were selected for further expansion and characterization (Supplementary Fig. 1a). The reprogramming of patient cells into iPSC was approved by the Swedish work environment authority. All individuals carried the common neutral isoform ApoE3/E3 of the *APOE* gene. An additional female control line (TALSCTRL15.12) was received from Target ALS (http://www.targetals.org/) and NHCDR Repositories hosted by RUCDR.

Alkaline phosphatase activity
Alkaline phosphatase activity was detected using Alkaline Phosphatase Staining Kit (Stemgent, MA) according to the manufacturer's protocol.

Karyotype analysis
The G-banding analysis was performed at 300–400 band resolution in a clinical diagnostic setting.

Variation sequencing
Genomic DNA was extracted from fibroblasts and iPSCs with the use of conventional lysis buffer composed of 100 mM Tris (pH 8.0), 200 mM NaCl, 5 mM EDTA, 1.5 mg/ml Proteinase K, and 0.2% SDS in distilled autoclaved water. The presence of APP and PS1 variations was confirmed by direct DNA sequencing (Macrogen Europe, Amsterdam, The Netherlands).

DNA fingerprinting
DNA fingerprinting analysis was performed by the IdentiCell STR profiling service (Department of Molecular Medicine, Aarhus University Hospital, Skejby, Denmark). In vitro differentiation by embryoid body (EB) formation iPSCs were transferred onto ultra-low adherent 24-well plates (Corning) and for 14 days were grown as free-floating embryoid bodies (EBs) in WiCell medium supplemented with 20ng/ml FGF2. For subsequent spontaneous differentiation, EBs were seeded on a 0.1% gelatin-coated 96-well plate in DMEM media containing 10% fetal bovine serum (v/v) and 1% Penicillin-Streptomycin (v/v). Two weeks later, the cells were fixed and immunostained for germ layer markers.

Differentiation of iPSCs to hippocampal spheroids
For differentiation to HSs, one to three clones per individuals were thawed and expanded over several passages. To form EBs, intact iPSC colonies were dissociated using dispase (1 mg/ml) for ~20 minutes and transferred into ultra-low adherent flasks (Corning) in WiCell medium supplemented with 20 ng/ml FGF2 and 20μM ROCK-Inhibitor Y-27632 (Selleck Chemicals, Munich, Germany). Next day, WiCell was replaced with neural induction medium (NIM) composed of advanced DMEM/F12, 2% B27 Supplement without vitamin A (v/v), 1% N2 Supplement (v/v), 1% NEAA (v/v), 2 mM L-glutamine and 1% Penicillin-Streptomycin (v/v). For dorsomedial telencephalic neural specification, LDN-193189 (Stemgent, 0.1 μM), Cyclopamine (Selleck Chemicals, 1 μM), SB431542 (Sigma-Aldrich, 10 μM) and XAV-939 (Tocris, 5 μM) were added to the medium for the first ten days with medium change every other day. On the tenth day, the free-floating spheres were transferred to neuronal differentiation medium (NDM) containing Neurobasal® medium, 1% N2 (v/v), 1% NEAA (v/v), L-glutamine and 1% Penicillin-Streptomycin (v/v). To promote hippocampal differentiation, NDM was

supplemented with CHIR-99021 (Stemgent, 0.5 mM) and brain derived neurotrophic factor (BDNF, PeproTech, 20 ng/ml) for 90 days with medium changes every second day. On the day in vitro 50, spheroids were dissociated into single cells with Trypsin 1X and plated in NDM on polyornithine/laminin coated surfaces. Adherent cells were grown for 6 days in NDM supplemented with CHIR-99021 (0.5 mM) and BDNF (20 ng/ml) with media change every second day.

Differentiation of iPSCs to astrocytes
On the day in vitro 50, spheroids were dissociated and transferred into new ultra-low adherent flask in neural expansion medium (NEM) containing DMEM/F12, 2% B27 without vitamin A (v/v), 1% NEAA (v/v), 2 mM L-glutamine, 1% Penicillin-Streptomycin (v/v), and 0.2 µg/ml heparin (Sigma-Aldrich). NEM was supplemented with 20 ng/ml FGF2 and 20ng/ml EGF (Peprotech) for 30 days with media change every 2-3 days. On the day in vitro 80, free-floating spheroids were dissociated into single cells and seeded to polyornithine/laminin coated culture flasks in NDM. To promote astroglial differentiation, NDM was supplemented with ciliary neurotrophic factor (CNTF, R&D Systems, 20 ng/ml) for 40 days with media change every 2-3 days. Cells were passaged at confluency.

In vivo transplantation
For in vivo transplantation, male 10–12 weeks old RAG-1-deficient mice (n=3) generated by Lund University breeding facility were used. All experimental procedures were conducted in accordance with the European Union Directive (2010/63/EU) on the subject of animal rights, and were approved by the committees for the use of laboratory animals at Lund University and the Swedish Board of Agriculture. On the day in vitro 50, spheroids were dissociated into single cells and resuspended in PBS to a final concentration of 100 000 cells/µl. The mice were anesthetized with isoflurane (Baxter, Deerfield, IL) in oxygen (initial dose of 5% which was reduced to 1–1.5% for maintenance of surgical depth anesthesia) delivered through a nose mask during the surgery. The body temperature was maintained at 37.0°C during the surgery using a thermostatically controlled rectal probe connected to a homeothermic blanket. The skull was exposed by the skin incision after subcutaneous analgesia with Marcaine (50 µl of 2.5 mg/ml stock solution, Astra Zeneca). Transplantation was stereotaxically performed for each mouse through drilled hole in the skull using a 5-µl Hamilton syringe with 32-gauge needle and an injecting minipump ((Nanomite Injector
Syringe Pump; Harvard Apparatus, Holliston, MA, USA). A volume of 2 µl of cell suspension was injected at a rate of 0.5 µl/min at the following coordinates (from bregma and brain surface): anterior/posterior (AP): -2.54 mm; medial/lateral (M/L): -1.5 mm; dorsal/ventral (D/V): -1-75 mm. The needle was left in situ for 7 min after injection before being slowly raised, and the wound was sutured.

Rodent brain tissue collection and processing
Five weeks after transplantation, the mice were transcardially perfused with PBS followed by perfusion with 50 ml of 4 % paraformaldehyde (PFA). The dissected brains were post-fixed in the same fixative overnight at 4°C. After fixation, brains were cryoprotected in 30% sucrose (Sigma- Aldrich) for 48 hours at 4°C before being cut into 40 µm thick coronal serial sections on a sliding microtome (Leica Microsystems GmH, Wetzlar Germany). The free-floating brain sections were then stored in antifreeze solution at -20°C until immunohistochemical staining.

Immunostainings, microscopy and image analyses
For immunocytochemistry, the adherent cells aged 56 days were fixed with 2% PFA for 10 min followed by fixation with 4% PFA for 10 min at RT, blocked for 1hr at RT with 10% normal

donkey (NDS, VWR) or normal goat serum (NGS) in PBS with 0.1% Triton-X (Sigma-Aldrich) and incubated overnight with target primary antibodies (Supplementary Table 6) prepared in 10% NDS/NGS in PBS at 4ºC. On the next day, the cells were incubated with appropriate Alexa-fluor 488, 555- or 647- conjugated secondary antibodies (Supplementary Table 6) in PBS for 1hr at RT in the dark. Cell nuclei were counterstained with DAPI (1:10,000). Spheroids aged 100 days were fixed for 45 minutes with 4% PFA at 4ºC followed by incubation in PBS overnight. Next day, spheroids were cryoprotected with 30% sucrose for 4-6 hours, embedded in Tissue-Tek O.C.T. Compound (Sakura Finetek, Zouterwoude, the Netherlands), snap frozen on liquid nitrogen and cryostat (Leica Microsystems GmH, Wetzlar Germany) at 30 µm and then stored at -80°C until immunohistochemical staining. For immunocytochemistry, frozen sections were air-dried for 1 hour, washed 3 times in PBS with 0.5% Tween (PBST) pH 7.4 and permeabilized with 0.3% Triton X-100 (for spheroid sections) or 0.4% Triton X-100 (for mouse brain sections) for 30 min followed by blocking with 10% NDS in PBST and incubation with target primary antibodies (Supplementary Table 6) in 10% NDS in PBST at RT with slow agitation overnight. For staining detecting human nuclei (HuNu), M.O.M.TM Mouse Ig Blocking Reagent (Vector Laboratories) was used to block endogenous mouse antibody in the brain sections. On the next day, sections were incubated with appropriate Alexa-fluor 488-, 555- or 647-conjugated secondary antibody (Supplementary Table 6) in 10% NDS in PBST at RT for 1 hour. Image acquisition was performed using inverted epifluorescence microscope LRI-Olympus IX- 73 or confocal microscope (Carl Zeiss, Germany). Automated quantitative image analysis of stained cell cultures was performed with the MetaMorph Software V7.6 (Molecular Devices) using the Multi-Wavelength Cell Scoring application. For a specific marker, positive cells were identified as having signal intensity above the selected intensity threshold. Intensity thresholds were set blinded to sample identity. Three-dimensional surface reconstructions of confocal z-stacks were created using the surface object and filament functions in Imaris software 8.4.1 (Bitplane).

Morphometric analysis

All morphometric analyses were performed using ImageJ software on the adherent cells aged 56 days stained with the MAP2 antibody. For soma area calculation, the Map2-positive cell bodies were manually outlined and the area within was measured. The lengths of all individual dendrites were counted and summed to estimate total dendrite length. To assess arborization, dendrites were subjected to Sholl analysis using the Simple Neurite Tracer plugin (Ferreira et al., 2014). Briefly, concentric circles were drawn around the cell center with increasing radii and the algorithm then counts the number of intersections of the binarized neurite skeletons with the concentric circles.

Electrophysiology

Electrophysiology recordings were performed on the adherent cells aged 56-58 days. Coverslips were placed in the recording chamber during recordings and constantly perfused with carbogenated artificial cerebral spinal fluid (ACSF, in mM: 119 NaCl, 2.5 KCl, 1.3 $MgSO_4$, 2.5 $CaCl_2$, 26 $NaHCO_3$, 1.25 NaH_2PO_4, and 11 glucose, pH ~7.4) at 34°C. Whole-cell patch-clamp recordings were performed using a potassium gluconate-based intracellular solution (in mM: 122.5 potassium gluconate, 12.5 KCl, 10 HEPES, 2.0 Na_2ATP, 0.3 Na_2-GTP, and 8.0 NaCl). Biocytin (1-3mg/ml, Biotium) was dissolved in the pipette solution for post-hoc identification of recorded cells. Whole-cell patch-clamp recordings were performed with a HEKA double patch clamp EPC10 amplifier using PatchMaster for data acquisition. Voltage and current clamp recordings were used for the electrophysiological characterization. Sodium and potassium currents were evoked by a series of 200ms long voltage steps ranging from -70mV to +40mV in 10mV steps and their sensitivity to, respectively, 1µM TTx (Latoxan) and

10mM TEA (Abcam) were determined. A series of current steps, going from 0pA to 190pA in 10pA steps and lasting 500ms, were performed from a membrane potential of approximately -70mV to determine the cells ability to generate APs. To study the AP characteristics a current ramp protocol going from 0-300pA in 1s was used. Current was injected, when needed, to keep the membrane potential around -70mV. Data were analyzed offline with FitMaster (HEKA Elektronik, Lambrecht, Germany) and IgorPro (Wavemet— rics, Lake Oswego, OR, USA).

Quantitative RT-PCR
Expression of miRNAs was assessed by qRT-PCR in the adherent cells aged 56 days. Total RNA was extracted from cells using TRIzol® (LifeTechnologies, Carlsbad, CA, USA), according to manufacturer's instructions and as previously described (Gomes et al., 2019). Total RNA was quantified using Nanodrop ND-100 Spectrophotometer (NanoDrop Technologies, Wilmington, DE, USA) and conversion of cDNA was achieved with the miRCURY LNA RT Kit (Qiagen, Hilden, Germany), using 5 ng total RNA. The Power SYBR® Green PCR Master Mix (Applied Biosystems) were used in combination with predesigned primers (Qiagen), summarized in Supplementary Table 7, using as reference genes U6, RNU1A1 and a RNA-spike provided with the kit (all from Qiagen). The reaction conditions consisted of polymerase activation/denaturation and well-factor determination at 95ºC for 10 min, followed by 50 amplification cycles at 95ºC for 10 s and 60ºC for 1 min (ramprate 1.6º/s). Each sample was performed in triplicate, and a non-template control was included for miRNA analysis. The expression fold change vs. respective controls was determined by the 2- ΔΔCT equation.

MSD MULTI-ARRAY Assay
Culture media (CM) were collected from HSs aged 100 days at four days after the last medium change. The pellets of HSs were lysed with M-PER Mammalian Protein Extraction Reagent complemented with Halt Protease Inhibitor Cocktail (100X). Total protein was determined using the PierceTM BCA Protein Assay Kit according to the manufacturer instructions. Aβ levels were quantified in CM and HS lysates using the multiplex Aβ Peptide Panel 1 (6E10, MesoScale Discovery, USA) according to the manufacturer's protocol. Samples below or above the sensitivity of the assay were set to zero or the max value, respectively. Concentrations were normalized to total protein (pg/µg protein) and averaged between three wells per differentiation.

Western blot analysis
Spheroids aged 100 days were lysed with RIPA buffer or M-PER Mammalian Protein Extraction Reagent complemented with Halt Protease Inhibitor and Halt Phosphatase Inhibitor Cocktails. Protein concentrations were determined using the PierceTM BCA Protein Assay Kit according to the manufacturer instructions. Samples were mixed with loading buffer, heated at 70 °C for 10 min and loaded into 10–20% Tricine gels. After electrophoresis the samples were transferred to polyvinylidine difluoride (PVDF) membranes. Membranes were blocked in PBS containing 0.1% Tween-20 (PBST) and 5% milk or 5 % BSA, and incubated with target primary antibodies (Supplementary Table 8) overnight. Next day, membranes were incubated with HRP-conjugated secondary antibodies (Supplementary Table 8) for 1 h diluted in PBST and 5% milk or 5% BSA. The blots were then visualized using Pierce Enhanced Chemo-Luminescence solution and imaged in a Biorad Chemi-Doc chemo-luminescence system (BioRad). Bands were quantified using Image Lab (Bio-Rad Laboratories).

Fourier transform infrared micro-spectroscopy
FTIR is a spectroscopic tool that can be used to detect molecular vibrations of main-chain carbonyl groups, that occur in the wave number range of 1500 to 1700 cm^{-1} (Amide I and II

regions), allowing the detection of β-sheet structures (Baker et al., 2014; Miller et al., 2013). FTIR further allows to determine protein aggregation measured as average of β-sheet structures (1628 cm^{-1}) to total protein (1656 cm^{-1}) absorption. FTIR micro-spectroscopy analyses were performed on the parental fibroblasts, neurons aged 56 days, astrocytes aged 120 days, and HSs aged 100 days, and using two different platforms. Cell pellets were homogenized, spread (1 µl) on the 1 mm thick CaF_2 spectrophotometric window and dried under nitrogen flow. For reproducibility, infrared spectra were taken from different areas of the cell pellet deposited on CaF_2. The HSs spectra were recorded on a Hyperion 3000 IR microscope (BrukerScientific Instruments, Billerica, MA, USA) coupled to a Tensor 27, which was used as the IR light source with 15Å~ IR objective and MCT (mercury cadmium telluride) detector, located at Lund University (Sweden). The measuring range was 900–4000 cm^{-1}, the spectra collection was done in transmission mode at 4 cm^{-1} resolution from 250-500 co-added scans. For the parental fibroblasts, neurons and astrocytes FTIR spectro-microscopy was performed at the SMIS beamline of the SOLEIL synchrotron (France) using a Thermo Fisher Scientific Continuum XL FTIR microscope through a 32× magnification, 0.65 NA Schwarzschild objective. For the collection parameters were: spectral range 1000−4000 cm^{-1}, in transmission mode at 4 cm^{-1} spectral resolution, with 8 µm × 8 µm aperture dimensions, using 256 coadded scans. Background spectra were collected from a clean area of the same CaF_2 window. All measurements were made at room temperature. For analysis of FTIR spectra OPUS software (Bruker) and Orange (University of Ljubljana) were used and included atmospheric compensation. Derivation of the spectra to the second order using Savitsky-Golay of 3rd polynomial order 3 with 9 smoothing points, was used to unmask the number of discriminative features and to eliminate the baseline contribution.

Viral vectors, gene transfer and FACS-based purification of GFP-positive cells

Spheroids aged 30 DIV were dissociated into single cells and plated in NDM on poly-ornithine/laminin coated surfaces. Two hours later the cells were transduced with either the eGFP or ND1-expressing Moloney murine leukemia-derived retroviral vectors (pCMMP-IRES2eGFP-WPRE or pCMMP-NeuroD1-IRES2eGFP-WPRE) at a multiplicity of infection (MOI) of 5, in proliferation medium supplemented by protamine-sulfate (4 mg/ ml, Sigma-Aldrich). The viruses were produced and titrated as previously described (Roybon et al., 2009). Adherent cells were then cultured for 20 days in NDM supplemented with CHIR-99021 and BDNF. Media was changed every other day. Twenty days later, the cells were detached with Trypsin 1X, resuspended in Neurobasal medium without red-phenol and transferred to a FACS tube. After centrifugation, the pellet was resuspended in Neurobasal medium without red-phenol containing 10% normal serum. For exclusion of dead cells, fluorescent live-cell impermeant DNA dye (DRAQ7, BioStatus, 1:1000) was added, and the cells were incubated on ice for 5 min before sorting. Samples were analyzed using a BD FACSAria III (BD Biosciences) with FACSDiva v8.0 software (BD Biosciences) at the MultiPark Cellomics and Flow Cytometry Core technical platform, at Lund University. The GFP-positive cells were collected in Eppendorf tubes, snap-frozen in liquid nitrogen and stored at -80°C until RNA extraction.

Affymetrix GeneChip microarray

Total RNA was extracted from the FACS-sorted GFP+ transduced cells using RNeasy Micro Kit (QIAGEN, Hilden, Germany), according to the manufacturer instructions. After adding 350µl buffer RLT containing 1% beta-mercaptoethanol, the thawed samples were mixed using a vortex and homogenized with QIAshredder spin columns. Next, 1 volume of 70% ethanol was added and the samples were applied to RNeasy MinElute spin columns followed by an on-column DNase digestion and several wash steps. Finally, total RNA was eluted in 14 µl of

nuclease free water. Purity and integrity of the RNA was assessed on the Agilent 2100 Bioanalyzer with the RNA 6000 Nano LabChip reagent set (Agilent, Palo Alto, CA, USA). Sample preparation for microarray hybridization was carried out as described in the Affymetrix GeneChip WT PLUS Reagent Kit User Manual (Affymetrix, Inc., Santa Clara, CA, USA). In brief, 200 ng of total RNA was used to generate double-stranded cDNA. 15 µg of subsequently synthesized cRNA was purified and reverse transcribed into sense-strand (ss) cDNA, whereat unnatural dUTP residues were incorporated. Purified ss cDNA was fragmented using a combination of uracil DNA glycosylase (UDG) and apurinic/apyrimidinic endonuclease 1 (APE 1) followed by a terminal labeling with biotin. 5.5 µg fragmented and labeled ss cDNA were hybridized to Affymetrix Clariom D human arrays for 16 h at 45° C and 60 rpm in a GeneChip hybridization oven 640. Hybridized arrays were washed and stained in an Affymetrix Fluidics Station FS450, and the fluorescent signals were measured with an Affymetrix GeneChip Scanner 3000 7G. Fluidics and scan functions were controlled by the Affymetrix GeneChip Command Console v4.3.3 software. RNA extraction and sample processing were performed at an Affymetrix Service Provider and Core Facility, "KFB - Center of Excellence for Fluorescent Bioanalytics" (Regensburg, Germany; www.kfbregensburg.de).
Summarized probe set signals in log2 scale were calculated by using the GCCN-SST-RMA algorithm with the Affymetrix GeneChip Expression Console v1.4 Software. The output matrix was processed in Perseus framework. To simplify the analysis, we examined transcriptomic changes for annotated genes having a gene name and a protein accession number (representing a total of 21,467 genes out of the 135,750 probes examined). The 2D annotation enrichment analysis was performed using the biological pathways information available in KEGG repository and the procedure was provided in Perseus software. Briefly, the average intensity was calculated for each transcript in the four groups (Control:GFP, Control:ND1, APP:GFP, and APP:ND1). Later, the GFP values were subtracted from their corresponding ND1 values in both control and APP variant. The resulting values correspond to the difference between ND1 and GFP in both control and APP variant. For the enrichment analysis, pathways significantly enriched were filtered through a FDR (Benjamini-Hochberg) with a q-value threshold of 0.01.

Label-free liquid chromatography-tandem mass spectrometry
100 µl of lysis buffer (50 mM dithiothreitol (DTT) and 2 w/v% sodium dodecyl sulfate (SDS) in 100 mM Tris-Cl buffer, pH 8.6) was added to the spheroid sample. The sample was boiled at 95 °C for 5 minutes, then proteins were extracted by sonication executing 30 cycles of 15s on and 15 s off at 4 °C using a Bioruptor plus (model UCD-300) (Diagenode). Iodoacetamide (IAA) was added to the lysate at the final concentration of 20 mM and was incubated at RT for 30 minutes in dark. The proteins were precipitated using 9 volumes of cold ethanol O/N at -20 °C and the protein pellet was dissolved in 50 mM ammonium-bicarbonate (AmBic) with 0.5% SDC (sodium deoxycholate). The protein concentration was measured using the Micro BCA Protein Assay Kit (Thermo Scientific) prior the digestion. Sequencing Grade Modified Trypsin (Promega, Madison, WI) was added to the sample in 1:25 enzyme-substrate ratio and was incubated for 18 hours at 37 °C. SDC was removed by EtOAc (ethyl acetate) extraction under acidic conditions (Gil et al., 2017). Peptide concentration of the sample was measured using the Pierce Quantitative Colorimetric Peptide Assay. The peptide samples were analyzed on a Q Exactive HF-X mass spectrometer coupled to an Ultimate 3000 RSCLnano pump (Thermo Scientific). Acclaim PepMap100 C18 (5 µm, 100 Å, 75 µm i.d. x 2 cm, nanoViper) was used as trap column and EASY-spray RSLC C18 (2 µm, 100 Å, 75 µm i.d. x 25 cm) as analytical column. Solvent A was 0.1% formic acid (FA), solvent B was 80% acetonitrile (ACN) with 0.08% FA. The flow-rate was set to 0.3 µl/min and the column temperature was 45 °C. The peptides were separated using a 120 min non-linear gradient and analyzed with a top 20 DDA (data dependent acquisition) method. MS1 scans were acquired using a mass range of 375-1500

m/z, with a resolution of 120,000 (@ 200 m/z), a target AGC value of 3e06 and a maximum injection time (IT) of 100 ms. The 20 most intense peaks were fragmented with a normalized collision energy (NCE) of 28. MS2 scans were acquired with a resolution of 15,000, a target AGC value of 1e05 and a maximum IT of 50 ms. The ion selection threshold was set to 8.00e03 and the dynamic exclusion to 40 s while single charged ions were excluded from the analysis. The precursor isolation window was set to 1.2 Th. The raw files were analyzed using Proteome Discoverer (PD) v2.2 (Thermo Scientific). Peptide and protein identification were performed against UniProt human database (released 20180207, 42213 sequences including isoforms) with SEQUEST HT as search engine. The following parameters were set during the search: max. 2 missed cleavages, cysteine carbamidomethylation as static modification, methionine oxidation, phosphorylation at serine, threonine and tyrosine as dynamic modifications, acetylation as a dynamic N-terminal protein modification, 10 ppm precursor mass tolerance and 0.02 Da fragment mass tolerance. 1% false discovery rate (FDR) at both peptide and protein levels was applied. For label-free quantitative analysis, protein abundance data was processed with the aid of Perseus v1.6.1.2. In order to normalize and standardize the data, raw intensities were log2 transformed and subtracted from the median of all proteins in each sample. To determine proteins with significant differences in abundance between samples under study, t-tests followed by FDR adjustment were performed. In all cases the q-value was set at 5%. The quantitative data was also used for principal component analysis and hierarchical clustering by means of Perseus. Groups of interesting proteins were submitted to biological processes and pathways enrichment analysis, performed through the online tool DAVID Bioinformatics Resources v6.8 (Huang da et al., 2009a, b).

Statistical analyses
All data were analyzed using GraphPad Prism 7 software and presented as mean ± S.E.M. with 2-5 independent differentiations per cell line (indicated in the figure legend). Unpaired two-tailed t-test or Mann-Whitney U test were used to compare two groups, repeated measures ANOVA was employed for Scholl analysis. A P-value of < 0.05 was considered significant.

References to supplemental experimental procedures

Baker, M.J., Trevisan, J., Bassan, P., Bhargava, R., Butler, H.J., Dorling, K.M., Fielden, P.R., Fogarty, S.W., Fullwood, N.J., Heys, K.A., *et al.* (2014). Using Fourier transform IR spectroscopy to analyze biological materials. Nat Protoc *9*, 1771-1791.

Gil, J., Ramirez-Torres, A., Chiappe, D., Luna-Penaloza, J., Fernandez-Reyes, F.C., Arcos-Encarnacion, B., Contreras, S., and Encarnacion-Guevara, S. (2017). Lysine acetylation stoichiometry and proteomics analyses reveal pathways regulated by sirtuin 1 in human cells. J Biol Chem *292*, 18129-18144.

Gomes, C., Cunha, C., Nascimento, F., Ribeiro, J.A., Vaz, A.R., and Brites, D. (2019). Cortical Neurotoxic Astrocytes with Early ALS Pathology and miR-146a Deficit Replicate Gliosis Markers of Symptomatic SOD1G93A Mouse Model. Mol Neurobiol *56*, 2137-2158.

Huang da, W., Sherman, B.T., and Lempicki, R.A. (2009a). Bioinformatics enrichment tools: paths toward the comprehensive functional analysis of large gene lists. Nucleic Acids Res *37*, 1-13.

Huang da, W., Sherman, B.T., and Lempicki, R.A. (2009b). Systematic and integrative analysis of large gene lists using DAVID bioinformatics resources. Nat Protoc *4*, 44-57.

Miller, L.M., Bourassa, M.W., and Smith, R.J. (2013). FTIR spectroscopic imaging of protein aggregation in living cells. Biochim Biophys Acta *1828*, 2339-2346.

Roybon, L., Hjalt, T., Stott, S., Guillemot, F., Li, J.Y., and Brundin, P. (2009). Neurogenin2 directs granule neuroblast production and amplification while NeuroD1 specifies neuronal fate during hippocampal neurogenesis. PLoS One *4*, e4779.

Supplementary Table 1. Electrophysiological characteristics of human iPSC-derived hippocampal neurons

	Control 1	PS1	Control 2	APP
**n**	23	23	26	23
**V_{rest} (mV)**	-38.28 ± 2,201	-36.76 ± 2.20	-39.94 ± 1.93	-38.41 ± 2.00
**R_{input} (MΩ)**	3116 ± 802	2229 ± 312	2981 ± 485	3365 ± 504
**C (pF)**	1.060 ± 0.139	1.488 ± 0.206	1.666 ± 0.349	1.215 ± 0.250
**AP threshold (mV)**	-20.42 ± 1.12	-18.54 ± 0.95	-24.24 ± 0.73	-20.83 ± 1.24*
**AP amplitude (mV)**	37.19 ± 1.93	35.96 ± 2.15	46.81 ± 3.25	34.67 ± 2.96*
**AP rise time (ms)**	1.71 ± 0.14	1.70 ± 0.12	2.03 ± 0.34	2.25 ± 0.30
**½ AP amp. width (ms)**	2.04 ± 0.17	1.99 ± 0.17	2.47 ± 0.30	2.75 ± 0.37
**AHP peak (mV)**	19.47 ± 4.62	22.63 ± 1.73	20.89 ± 1.56	20.71 ± 2.21

Abbreviations: V_{rest}, resting membrane potential, R_{input}, input resistance, C, membrane capacitance. n=23-26. P values: * = P<0.05.

Statistical analysis of V_{rest} (p=07251), Ri_{nput} (p=0.4481) and C (p=0.4763) by Kruskal-Wallis test show no significant differences between any of the four lines.

Control 1 vs PS1:
AP threshold, Unpaired t-test: p=0.2076
AP amplitude, Mann-Whitney test: p=0.5276
AP rise time, Mann-Whitney test: p=0.8448
½ AP amp. width, Mann-Whitney test: p=0.7482
AHP peak, Mann-Whitney test: p=0.9826.

Control 2 vs APP:
AP threshold, Unpaired t-test: p=0.0186
AP amplitude, unpaired t-test: p=0.0088
AP rise time, Mann-Whitney test: p=0.4440
½ AP amp. width, Mann-Whitney test: p=0.8504
AHP peak, unpaired t-test: p=0.9461

Supplementary Table 6. Primary and secondary antibodies used for immunocyto- and immunohistochemistry.

Antibody	Species	Type	Dilution	Manufacturer	Reference
**Primary antibodies**					
AFP	Mouse	Monoclonal	1:500	Sigma-Aldrich	A8452
AT8	Mouse	Monoclonal	1:500	Thermo Fisher	MN1020
Beta-III-tubulin	Mouse	Monoclonal	1:500	Sigma-Aldrich	T8660
Calbindin	Mouse	Monoclonal	1:500	Sigma Aldrich	C9848
Calretinin	Rabbit	Polyclonal	1:500	Swant	CR7697
Doublecortin	Rabbit	Monoclonal	1:400	Cell Signalling	4604
Drebrin	Rabbit	Polyclonal	1:1000	Abcam	ab11068
GABA	Rabbit	Polyclonal	1:500	Thermo Fisher	PA5-32241
GFAP	Rabbit	Polyclonal	1:2000	DAKO	Z0334
NCAM	Mouse	Monoclonal	1:400	Santa Cruz	SC-106
Human nuclear antigen (hNuclei)	Mouse	Monoclonal	1:250	Merck Millipore	MAB1281
LEF1	Rabbit	Monoclonal	1:200	Cell Signalling	2230

MAP2	Chicken	Polyclonal	1:1000	Abcam	AB92434
NANOG, PE-conjugated	Mouse	Monoclonal	1:200	BD Biosciences	560483
O4	Mouse	Monoclonal	1:200	Gift from J. Goldman, Columbia University, NY	
OCT4	Mouse	Monoclonal	1:200	Merck Millipore	MAB4401
PAX6	Mouse	Monoclonal	1:400	Thermo Fisher	MA1-109
PROX1	Rabbit	Monoclonal	1:400	Abcam	ab199359
Sendai virus	Chicken	Polyclonal	1:1000	Abcam	ab33988
SMA	Mouse	Monoclonal	1:200	Sigma Aldrich	A2547
Synaptophysin	Mouse	Monoclonal	1:1000	Merck Millipore	MAB5258
TBR1	Rabbit	Polyclonal	1:500	Merck Millipore	AB10554
TRA-1-81	Mouse	Monoclonal	1:200	Thermo Fisher	41-1100
ZBTB20	Rabbit	Polyclonal	1:200	Sigma Aldrich	HPA016815
**Secondary antibodies**					
Anti-chicken Alexa Fluor® 488	Donkey	Polyclonal	1:400	Jackson Immuno Research	703-545-155
Anti-mouse Alexa Fluor® 488	Donkey	Polyclonal	1:400	Thermo Fisher	A21202
Anti-rabbit Alexa Fluor® 488	Donkey	Polyclonal	1:400	Thermo Fisher	A21206
Anti-mouse Alexa Fluor® 555	Donkey	Polyclonal	1:400	Thermo Fisher	A31570
Anti-rabbit Alexa Fluor® 555	Donkey	Polyclonal	1:400	Thermo Fisher	A31572
Anti-chicken Alexa Fluor® 647	Donkey	Polyclonal	1:400	Merck Millipore	AP194SA6
Anti-mouse Alexa Fluor® 647	Donkey	Polyclonal	1:400	Thermo Fisher	A31571
Anti-chicken Alexa Fluor® 647	Goat	Polyclonal	1:500	Jackson Immuno Research	103-605-155
Anti-mouse Alexa Fluor® 647	Goat	Polyclonal	1:500	Jackson Immuno Research	115-605-146
Anti-mouse Alexa Fluor® 488	Goat	Polyclonal	1:500	Jackson Immuno Research	115-545-146
Anti-rabbit Alexa Fluor® Cy3	Goat	Polyclonal	1:500	Jackson Immuno Research	111-165-144
Streptavidin, Alexa Fluor™ 647 conjugate			1:400	Thermo Fisher	S32357

Supplementary Table 7. List of primer sequences used in qRT-PCR for microRNA expression

microRNA	Target Sequence
hsa-miR-21-5p	5’- UAGCUUAUCAGACUGAUGUUGA-3’
hsa-miR-29a-5p	5'ACUGAUUUCUUUUGGUGUUCAG-3’
hsa-miR-124-3p	5'UAAGGCACGCGGUGAAUGCC-3’
hsa-miR-125b-5p	5’-UCCCUGAGACCCUAACUUGUGA-3’
U6 snRNA	Reference gene
RNU1A1	Reference gene

Supplementary Table 8. Primary and secondary antibodies used for Western Blotting

Antibody	Species	Type	Dilution	Manufacturer	Reference
**Primary antibodies**					
Actin	Mouse	Monoclonal	1:1000	Sigma	A5316
AT8	Mouse	Monoclonal	1:1000	Thermo Fisher	MN1020
Drebrin	Rabbit	Polyclonal	1:1000	Abcam	ab11068
MAP2	Mouse	Monoclonal	1:1000	Sigma	M4403
PSD-95	Mouse	Monoclonal	1:1000	Merck Millipore	MAB1596
Synaptophysin	Mouse	Monoclonal	1:1000	Merck Millipore	MAB5258
**Secondary antibodies**					
Anti-mouse IGG HRP-conjugated	Goat	Polyclonal	1:1000	R&D Systems	HAF007
Anti-rabbit IGG HRP-conjugated	Goat	Polyclonal	1:1000	R&D Systems	HAF008

a

Diagnosis	Molecular analysis	Age at biopsy	Gender	iPSC line ID
Ctrl [Non demented healthy individual (NDHI)]	NA	65	Male	CSC-36C, D and E
Ctrl (NDHI)	NA	56	Female	CSC-37N and 37R
Ctrl (NDHI; Target ALS, cat# ND50004)	NA	49	Female	TALSCTRL15.12
See Assini et al., Neurology, 2003; 60;150	PS1, p.R278K	53	Male	CSC-12D, E and F
See Sorbi et al., Nature Genetics, 1993, v4	APP, p.V717I	50	Female	CSC-17F, F.1 and F.2

b

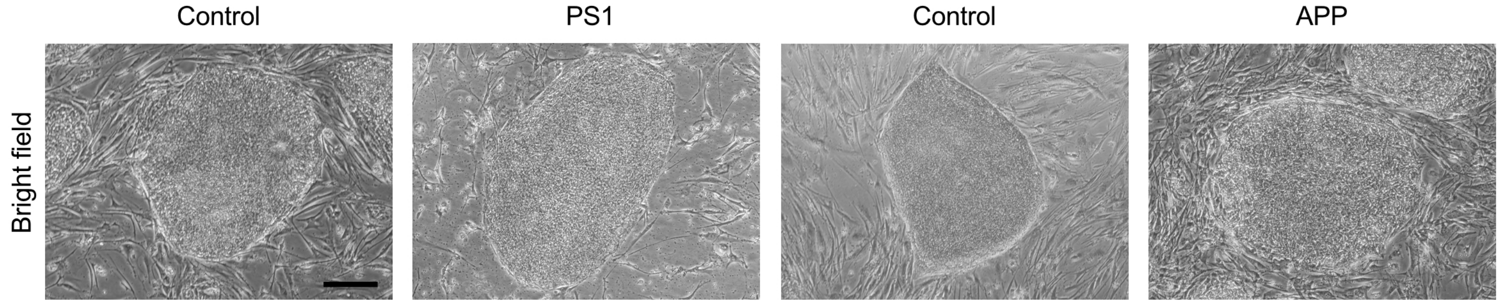

c

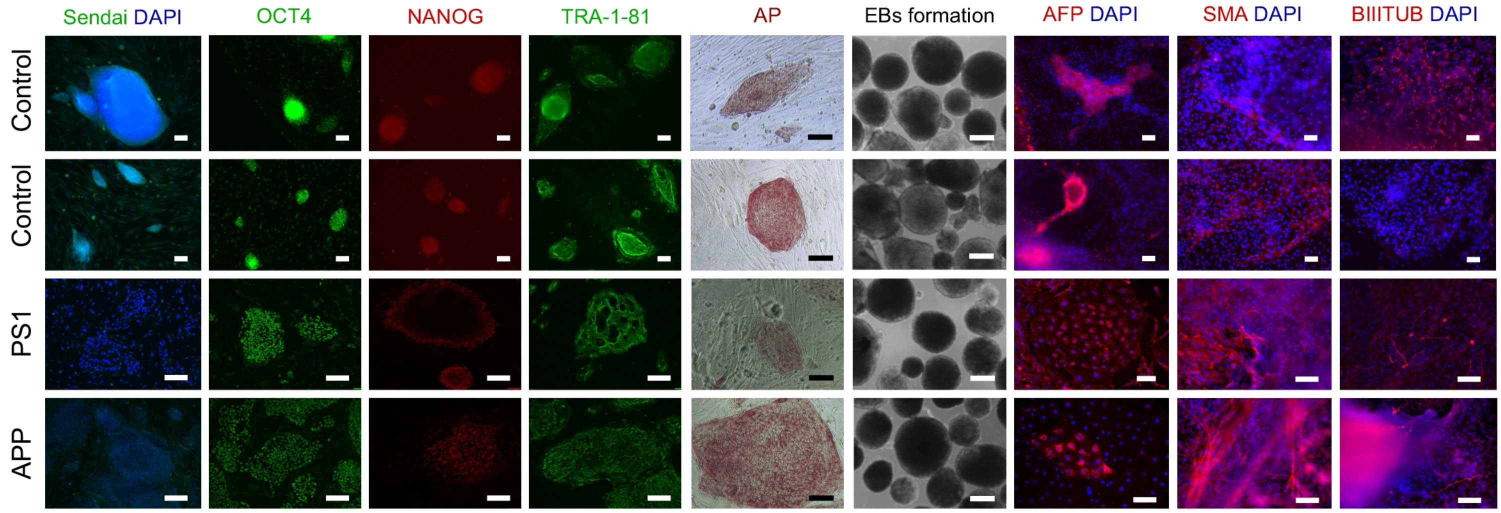

d

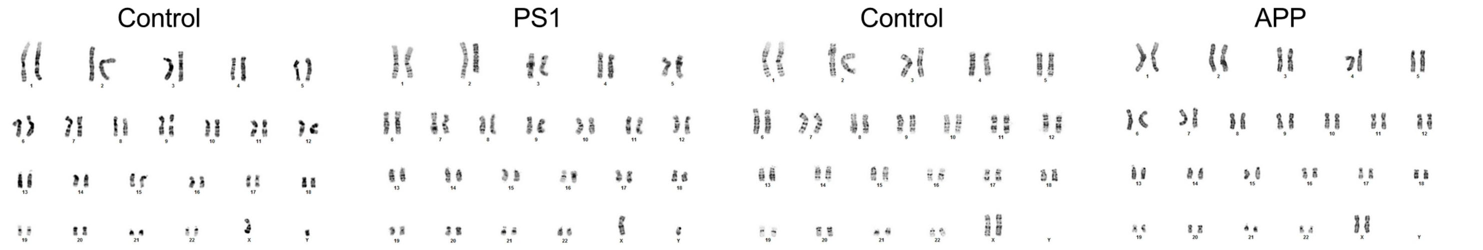

e

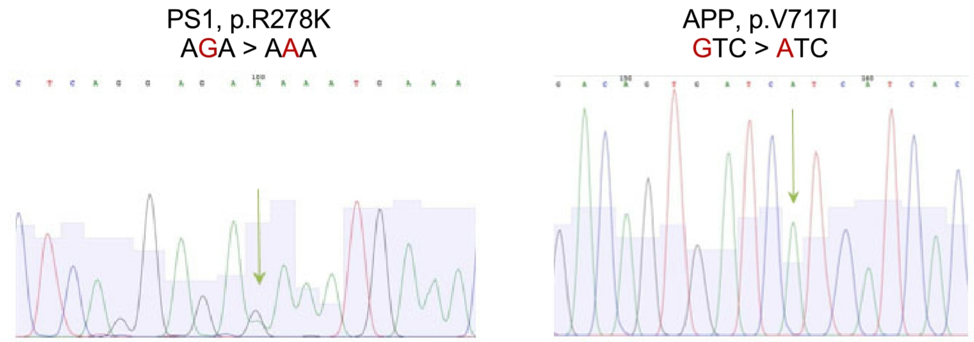

f

CSC-12 and parental fibroblasts

Marker	Allele(s)	
AMEL	X	Y
CSF1PO	10	11
D13S317	8	11
D16S539	10	12
D21S11	28	31.2

CSC-17 and parental fibroblasts

Marker	Allele(s)	
AMEL	X	
CSF1PO	12	
D13S317	10	12
D16S539	9	13
D21S11	29	30

Pomeshchik et al., Fig.S2

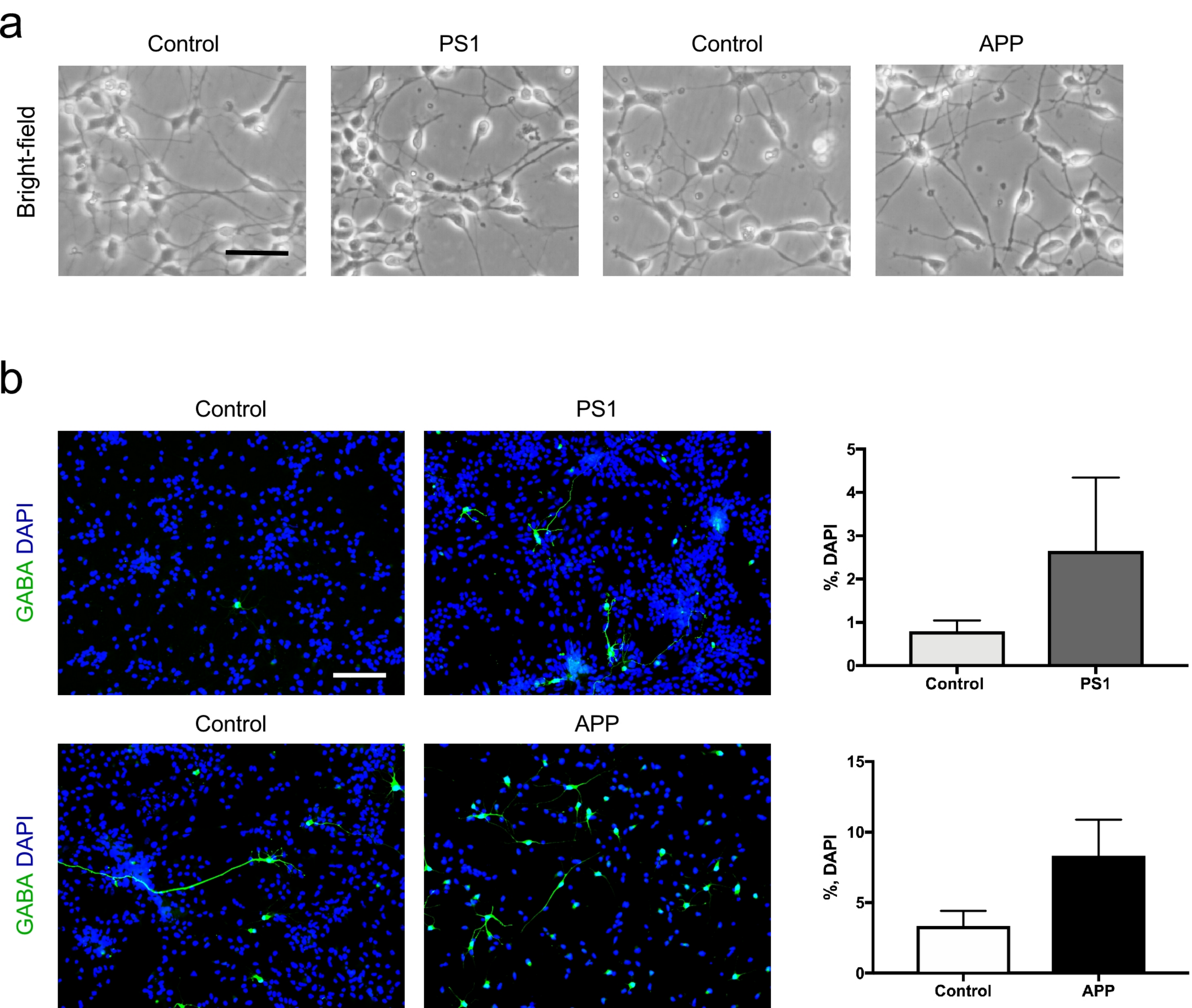

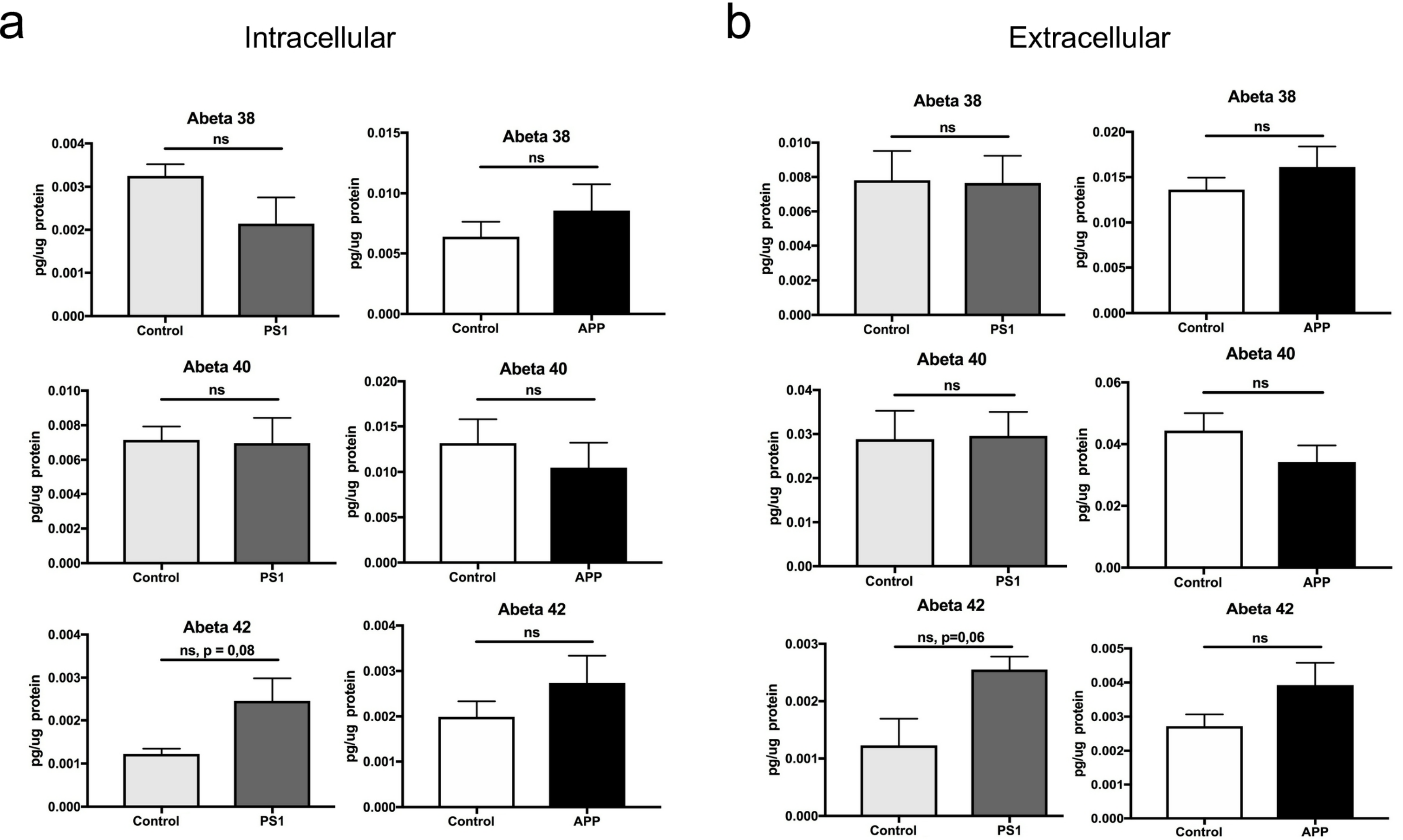
a
Intracellular
b
Extracellular
Abeta 38
Abeta 40
Abeta 42
ns
ns, p = 0,08
ns, p=0,06
pg/ug protein
Control
PS1
APP
0.000
0.001
0.002
0.003
0.004
0.005
0.006
0.008
0.010
0.015
0.020
0.00
0.01
0.02
0.03
0.04
0.06

Pomeshchik et al., Fig.S4

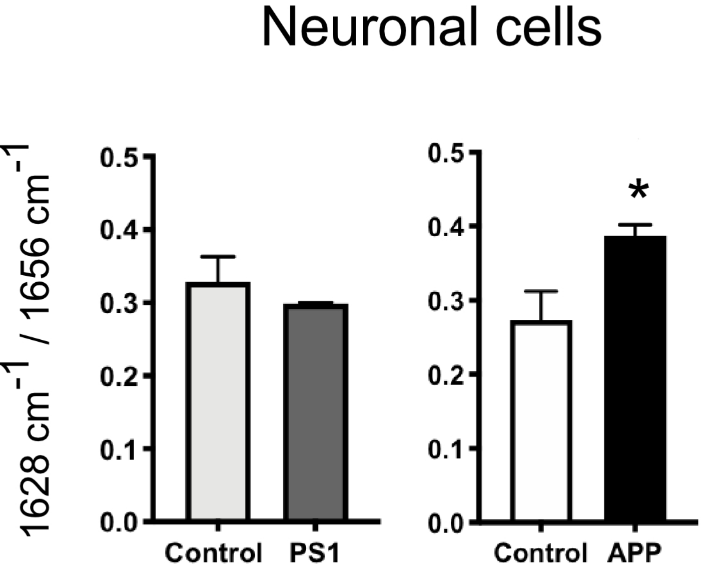

Supplement: Document S1. Supplemental Experimental Procedures, Figures S1–S4, and Tables S1 and S6–S8 [file mmc1.pdf]
