# Supplementary material for: Human iPSC-Derived Hippocampal Spheroids: An Innovative Tool for Stratifying Alzheimer Disease Patient-Specific Cellular Phenotypes and Developing Therapies
Source: Stem Cell Reports. 2020 Jun 25;15(1):256–73. doi: 10.1016/j.stemcr.2020.06.001 (PMC7363942; doi:10.1016/j.stemcr.2020.06.001)

Stem Cell Reports
Resource
OPEN ACCESS

Human iPSC-Derived Hippocampal Spheroids: An Innovative Tool for Stratifying Alzheimer Disease Patient-Specific Cellular Phenotypes and Developing Therapies

Yuriy Pomeshchik,[1,2,3] Oxana Klementieva,[2,4,5] Jeovanis Gil,[6] Isak Martinsson,[2,5] Marita Grønning Hansen,[3,7] Tessa de Vries,[1,2,3] Anna Sancho-Balsells,[1,2,3] Kaspar Russ,[1,2,3] Ekaterina Savchenko,[1,2,3] Anna Collin,[8] Ana Rita Vaz,[9,10] Silvia Bagnoli,[11,12] Benedetta Nacmias,[11,12] Claire Rampon,[13] Sandro Sorbi,[11,12] Dora Brites,[9,10] György Marko-Varga,[6] Zaal Kokaia,[3,7] Melinda Rezeli,[6] Gunnar K. Gouras,[2,5] and Laurent Roybon[1,2,3,*]

[1]iPSC Laboratory for CNS Disease Modeling, Department of Experimental Medical Science, BMC D10, Lund University, Lund SE-221 84, Sweden
[2]Strategic Research Area MultiPark, Lund University, Lund SE-221 84, Sweden
[3]Lund Stem Cell Center, Lund University, Lund SE-221 84, Sweden
[4]Medical Microspectroscopy, Department of Experimental Medical Science, BMC B11, Lund University, Lund SE-221 84, Sweden
[5]Experimental Dementia Research Unit, Department of Experimental Medical Science, BMC B11, Lund University, Lund SE-221 84, Sweden
[6]Clinical Protein Science and Imaging, Department of Biomedical Engineering, BMC D13, Lund University, Lund SE-221 84, Sweden
[7]Laboratory of Stem Cells and Restorative Neurology, Department of Clinical Sciences, BMC B10, Lund University, Lund SE-221 84, Sweden
[8]Department of Clinical Genetics and Pathology, Office for Medical Services, Lund SE-221 85, Sweden
[9]Research Institute for Medicines (iMed.ULisboa), Faculty of Pharmacy, Universidade de Lisboa, Lisbon, Portugal
[10]Department of Biochemistry and Human Biology, Faculty of Pharmacy, Universidade de Lisboa, Lisbon, Portugal
[11]Laboratorio di Neurogenetica, Dipartimento di Neuroscienze, Psicologia, Area del Farmaco e Salute del Bambino- NEUROFARBA, Università degli Studi di Firenze, Florence 50134, Italy
[12]IRCCS Fondazione Don Carlo Gnocchi, Florence, Italy
[13]Centre de Recherches sur la Cognition Animale (CRCA), Centre de Biologie Intégrative (CBI), Université de Toulouse; CNRS, UPS, Toulouse Cedex 9, France
*Correspondence: laurent.roybon@med.lu.se
https://doi.org/10.1016/j.stemcr.2020.06.001

SUMMARY

The hippocampus is important for memory formation and is severely affected in the brain with Alzheimer disease (AD). Our understanding of early pathogenic processes occurring in hippocampi in AD is limited due to tissue unavailability. Here, we report a chemical approach to rapidly generate free-floating hippocampal spheroids (HSs), from human induced pluripotent stem cells. When used to model AD, both APP and atypical PS1 variant HSs displayed increased Aβ42/Aβ40 peptide ratios and decreased synaptic protein levels, which are common features of AD. However, the two variants differed in tau hyperphosphorylation, protein aggregation, and protein network alterations. NeuroD1-mediated gene therapy in HSs-derived progenitors resulted in modulation of expression of numerous genes, including those involved in synaptic transmission. Thus, HSs can be harnessed to unravel the mechanisms underlying early pathogenic changes in the hippocampi of AD patients, and provide a robust platform for the development of therapeutic strategies targeting early stage AD.

INTRODUCTION

Alzheimer disease (AD) is the most common cause of dementia in the elderly, resulting in memory impairments and cognitive decline, and eventually leading to significant disability. Despite decades of intensive research, AD still remains incurable and represents a major clinical, social and economic problem (https://www.alzheimers.net/resources/alzheimers-statistics/).

The hippocampus is involved in the formation of new memories, learning, and emotions, and is one of the first regions of the brain that atrophies in AD (Mueller et al., 2010). However, since human brain tissue is generally available only postmortem, our understanding of early pathogenic events occurring in the hippocampus, specifically at the cellular level, is limited to brain imaging techniques (Boutet et al., 2014; Schuff et al., 2009). Hence, it is difficult to identify the mechanisms underlying cognitive impairment in AD. Transgenic rodents are commonly used as an experimental model of AD for preclinical investigation (Richetin et al., 2015; Tampellini et al., 2010). However, all therapies that were successful in rodent models have failed in human clinical trials, questioning whether rodent AD models are appropriate for modeling human AD (Morris et al., 2014).

Therefore, to develop efficient therapies for AD, we need to develop advanced models for preclinical investigations. These models should closely recapitulate human AD pathology, and allow investigation of early cellular changes, to identify druggable targets that could delay disease onset, and eventually overcome memory impairment. Even if minimalist, the models should mimic the human brain parenchyma and allow 3D interaction between mature cell types (Clevers, 2016; Sloan et al., 2017). Such models

This is an open access article under the CC BY license (http://creativecommons.org/licenses/by/4.0/).

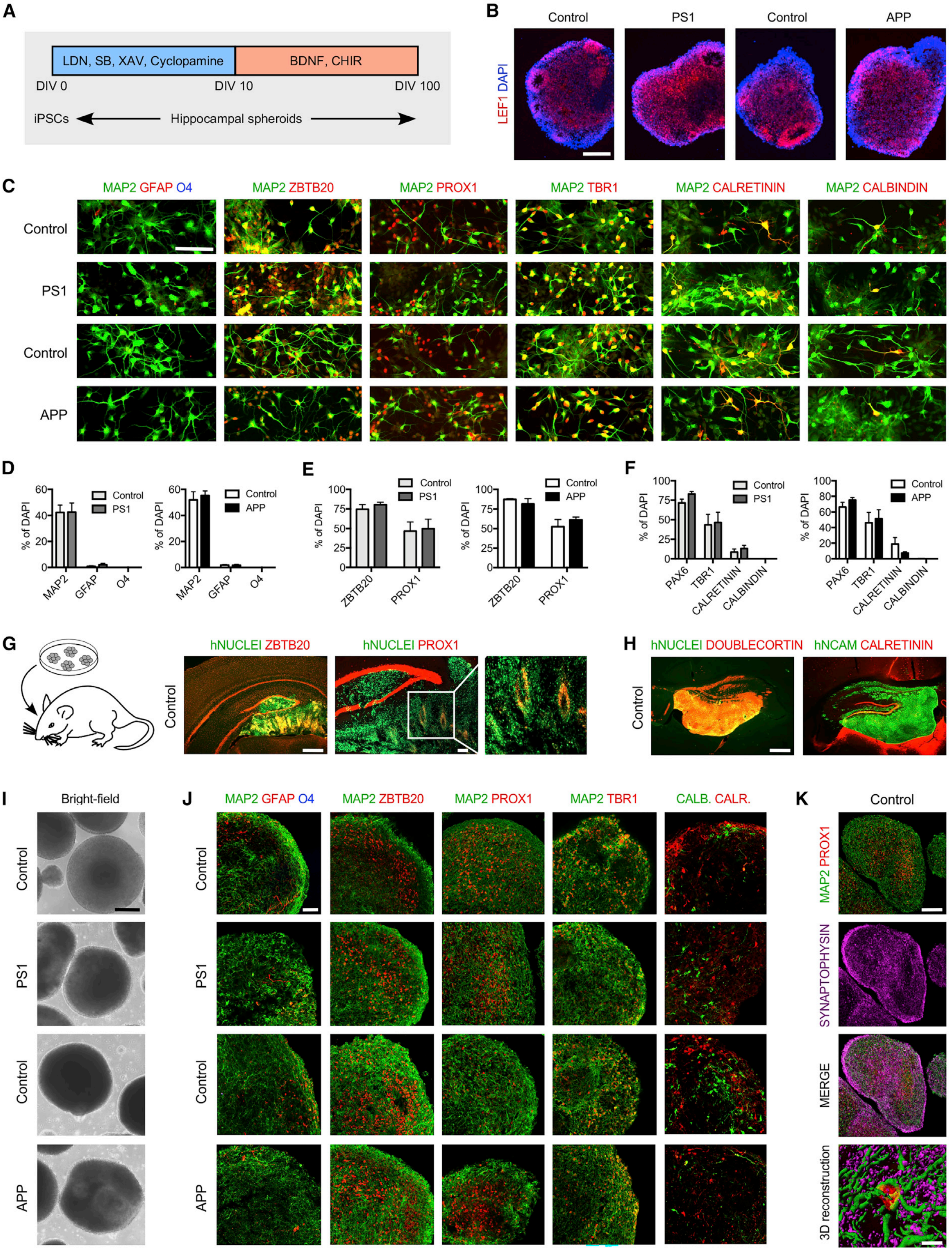

(legend on next page)

should be inexhaustible and scalable to provide enough material for different assays, including omics analyses. They should also be useful to develop therapeutic strategies. Induced pluripotent stem cells (iPSCs) harboring the genetic background of the patient they are derived from, are an attractive source of human material that can be used to generate physiologically relevant models for studying neurological diseases, including AD (Arber et al., 2017). Given the crucial role played by the hippocampus in AD, the development of human iPSC-based hippocampal cellular models would be an important step forward for AD research.

Here, we describe an innovative strategy allowing to differentiate human iPSCs into hippocampal spheroids (HSs), enriched in hippocampal neurons expressing the zinc finger and BTB domain-containing protein 20 (ZBTB20) and the prospero homeobox protein 1 (PROX1) (Lavado and Oliver, 2007; Nielsen et al., 2014). The HSs generated from two AD patients carrying variations in amyloid precursor protein (APP) or presenilin 1 (PS1) genes exhibited cardinal cellular pathological features of AD, including loss of synaptic proteins and increased ratio of intracellular and extracellular Aβ42/Aβ40 peptides. However, they also exhibited differences in protein aggregation measured by the non-destructive label-free Fourier transform infrared (FTIR) microspectroscopy (Baker et al., 2014), tau phosphorylation, miRNA pattern, and protein network alterations. Hippocampal neurons derived from APP variant HSs demonstrated profound transcriptomic alterations, which could be modulated by overexpression of NeuroD1 (ND1), resulting in the upregulation of genes, gene products of which are associated with synaptic transmission and are altered in AD (de Wilde et al., 2016).

RESULTS

Generation and Characterization of HSs from iPSCs of AD Patients and Healthy Individuals

To investigate the effect of PS1 and APP variation in human hippocampal cells, we generated iPSC lines from the skin fibroblasts of two patients, one female and one male, diagnosed with genetic AD (Figure S1), using well-established methodology (Djelloul et al., 2015; Holmqvist et al., 2016). The female patient carried a homozygous variation in the APP gene (APP p.V717I) (Sorbi et al., 1993). This variation, known as the AD London mutation, is the most common missense variation of the APP gene. The male patient with atypical AD carried a rare variation in the PS1 gene (PS1 p.R278K) (Assini et al., 2003). In addition, we generated multiple iPSC lines from age- and gender-matched non-demented healthy individuals.

Next, we developed an original protocol (Figure 1A) based on the embryoid (EB) culture methodology described by Pasca et al. (2015). To generate dorsomedial telencephalic neural precursors, the EBs were simultaneously treated with dual smad signaling chemical inhibitors LDN-193189 and SB-431542 (Chambers et al., 2009;

Figure 1. Generation and Characterization of HSs from AD Patient and Control Human iPSCs

(A) Schematic model of the differentiation paradigm to generate HSs from iPSCs. LDN, LDN-193189, SB, SB-431542, XAV, XAV-939, CHIR, and CHIR-99021.

(B) Immunostaining for medial pallium marker LEF1 in APP variant, PS1 variant, and gender-matched control EBs aged 30 DIV. Scale bar, 150 μm.

(C) Immunostaining for brain cell subtype markers GFAP, O4 and MAP2, hippocampal markers ZBTB20 and PROX1, and neuronal markers TBR1, calretinin, and calbindin in APP variant, PS1 variant, and gender-matched control cultures at DIV 56. Scale bar, 200 μm.

(D–F) Quantification of GFAP-positive, O4-positive, and MAP2-positive cells (D), ZBTB20-positive and PROX1-positive cells (E), and PAX6-positive, TBR1-positive, calretinin-positive, and calbindin-positive cells (F) expressed relative to the total number of DAPI-labeled cells in APP variant, PS1 variant, and gender-matched control cultures at DIV 56. Results are presented as mean ± SEM. n = 1–4 independent differentiations per clone for N = 3 iPSC clones per genotype. $^*p < 0.05$; N/D means not detected. Statistical analysis by two-tailed t test.

(G) Immunostaining for human nuclei marker and hippocampal markers ZBTB20 and PROX1 in control cells 5 weeks after transplantation into mouse hippocampus. Host mouse ZBTB20 and PROX1-positive cells are not colocalized with human nuclei marker. Scale bars, 100 μm.

(H) Immunostaining for human nuclei marker, human cell surface marker NCAM, and neuronal markers doublecortin and calretinin in control cells 5 weeks after transplantation into mouse hippocampus. Host mouse doublecortin and calretinin-positive cells are not colocalized with human markers. Scale bar, 100 μm.

(I) Bright-field images showing representative examples of APP variant, PS1 variant, and gender-matched control HSs at DIV 100. Scale bar, 200 μm.

(J) Confocal images showing representative examples of immunostaining for brain cell subtype markers GFAP, O4 and MAP2, hippocampal markers ZBTB20, PROX1, and neuronal markers TBR1, calretinin, and calbindin in APP variant, PS1 variant, and gender-matched control HSs at DIV 100. Scale bar, 50 μm.

(K) Immunostaining for neuronal marker MAP2, hippocampal granule neuron marker PROX1 and pre-synaptic marker synaptophysin in control HSs at DIV 100 and 3D surface reconstruction of confocal z stacks showing $PROX1^+$/$synaptophysin^+$/$MAP2^+$ granule neuron in control HSs. Scale bars, 100 μm (HS confocal images) and 10 μm (3D reconstruction).

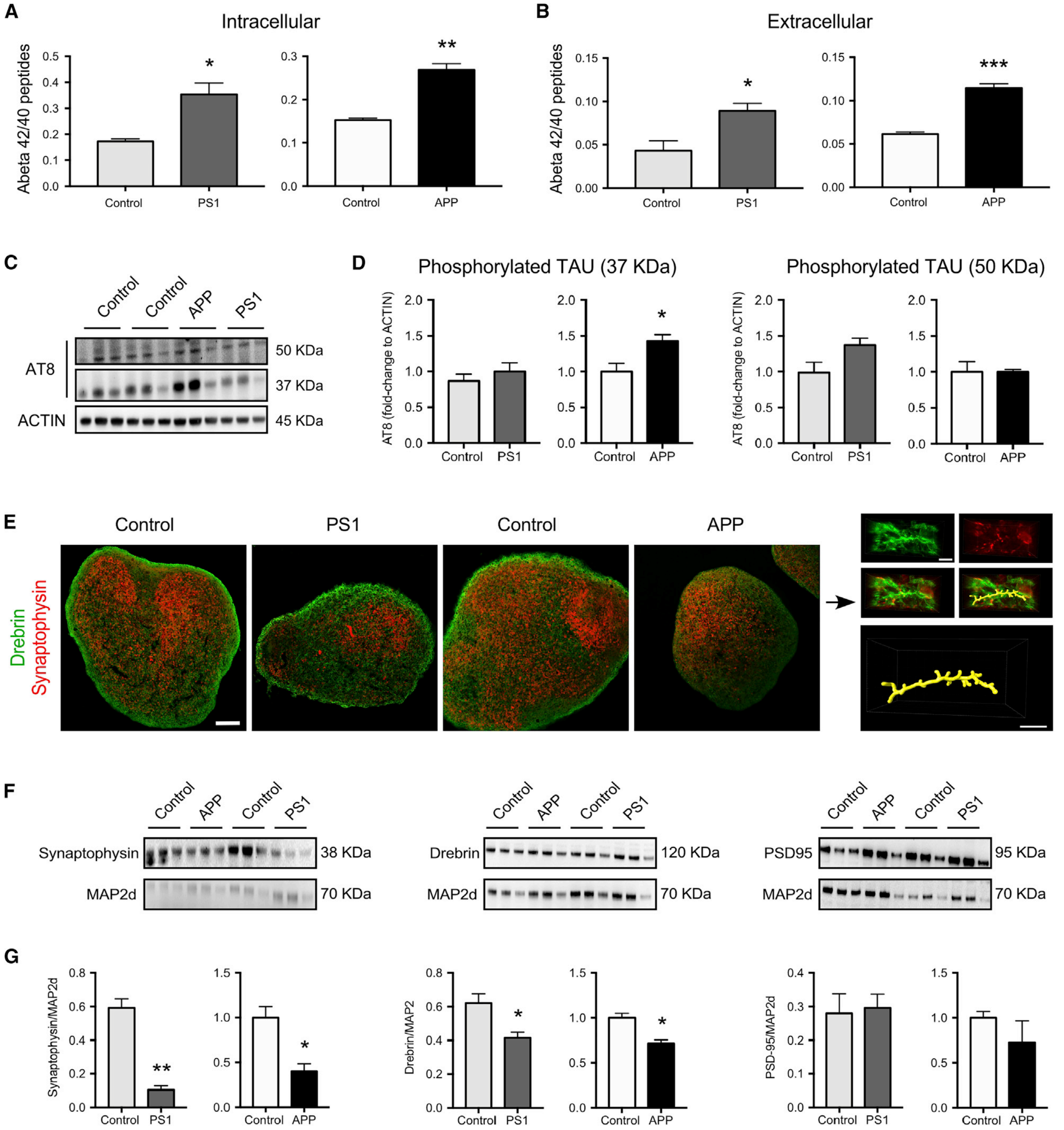

Figure 2. APP and PS1 Variant HSs Exhibit AD-Related Pathology

(A and B) Characterization of amyloid-β (Aβ) accumulation (intracellular Aβ, a) and secretion (extracellular Aβ, b) in APP variant, PS1 variant, and gender-matched control HSs at DIV 100. The ratio of Aβ42/Aβ40 in HS lysates (A) and the ratio of Aβ42/Aβ40 secreted from HSs into the medium (B) were measured at day 4 after the last medium change. For quantitation, data were normalized to the total protein. Results are presented as mean ± SEM. n = 3 independent differentiations per genotype. *p < 0.05, **p < 0.01, ***p < 0.001. Statistical analysis by two-tailed t test. See also Figure S2.

(C and D) Characterization of phosphorylation of tau protein in APP variant, PS1 variant, and gender-matched control HSs. Western blotting analysis of phosphorylation of tau protein in HSs at DIV 100 with actin blot included as a loading control (C). The blots were

(legend continued on next page)

Roybon et al., 2013). Dickkopf-specific chemical agonist XAV-939 (Nicoleau et al., 2013) and smoothen-binding chemical inhibitor of hedgehog signaling cyclopamine (Chen et al., 2002) were used to antagonize the formation of caudal and ventral tissue, specifying the cells toward a dorsal telencephalic fate (Watanabe et al., 2005). To specify the hippocampal identity, EBs were exposed to CHIR-99021, a chemical activator of WNT signaling (Lee et al., 2000), and brain-derived neurotrophic factor to allow expansion of hippocampal neural progenitors (Bull and Bartlett, 2005).

EBs aged 30 days *in vitro* (DIV) were almost exclusively composed of LEF1-positive cells (Figure 1B), suggesting that our protocol led to the formation of neural progenitors regionalized toward medial pallium tissue (Abellan et al., 2014). Quantitative analysis reveals that the EBs contained mainly neurons (Figure S2A) that were positive for microtubule-associated protein 2 (MAP2). Glial fibrillary acidic protein (GFAP)-positive astrocytes represented less than 2% of the population, and O4-positive oligodendrocytes were absent (Figures 1C and 1D). Importantly, 90% of the cells were positive for ZBTB20 and 45%–60% were positive for PROX1 (Figures 1C and 1E). The cultures also contained PAX6-, TBR1-, calretinin-, and calbindin-positive cells (Figures 1C and 1F), indicating that the hippocampal cells were at different stages of maturation (Roybon et al., 2009b). Few GABA-positive cells were identified (Figure S2B).

To validate our finding, we transplanted single-cell suspension from 50-day-old EBs of one of the iPSC control lines (CSC-37N) into the hippocampi of adult RAG-1-deficient mice and examined the graft composition 5 weeks later. The grafted cells (human nuclei-positive) co-expressed ZBTB20 and PROX1 (Figure 1G), as well as doublecortin and calretinin (Figure 1H).

When aged 100 DIV, the EBs were large in size, with no obvious alterations (Figure 1I). Although they contained some GFAP-positive astrocytes, they were primarily composed by MAP2-positive neurons (Figure 1J) co-expressing ZBTB20 and PROX1. They also contained TBR1 and an almost equal ratio of calretinin/calbindin-positive cells (Figure 1J). Detailed analysis of EBs revealed the presence of pre-synaptic synaptophysin-positive puncta at the surface of MAP2/PROX1-positive neurons (Figure 1K). We named these HSs, and further examined their relevance for modeling AD.

APP and PS1 Variant HSs Exhibit AD-Related Pathology

At first, we measured the amount of extra- and intracellular Aβ40 and Aβ42 peptides present in 100 DIV HSs. Both APP and PS1 variant HSs and their culture supernatants contained Aβ peptides with a higher ratio of Aβ42/Aβ40 than control HSs (approximately 1.5-fold higher for PS1 variant and approximately 2-fold higher for APP variant; Figures 2A and 2B), which concurs with previous studies (Duering et al., 2005; Murphy and LeVine, 2010). The change in Aβ42/Aβ40 ratio was mainly due to increased levels of Aβ42 peptides (Figure S3), suggesting that the cells carrying both variations had altered metabolism. The levels of released and intracellular Aβ40 peptide were either not altered (as in PS1 variant HSs) or showed a trend toward decreased production (APP variant HSs) (Figure S2). We also examined changes in levels of Aβ38 peptide but found none (Figure S2).

We next examined the state of phosphorylation of tau, previously reported to be enhanced by oligomeric Aβ peptides (Jin et al., 2011), and which is the major protein of the neurofibrillary tangles in AD when in its hyperphosphorylated form (Mandelkow and Mandelkow, 2012). We performed western blotting, using the monoclonal antibody AT8, which recognizes phosphorylation of tau at both serine 202 and threonine 205 (Goedert et al., 1995), and detected two monomeric tau bands (at 50 and 37 kDa molecular weight) in both AD and control samples (Figures 2C and 2D). We found significantly higher levels of the 37-kDa phosphorylated tau protein isoform in APP variant HSs, as compared with gender-matched control HSs.

Since synaptic pathology is an early morphological change in the human AD postmortem brain (Forner et al., 2017; Scheff et al., 2014), we assessed several key synaptic proteins in the HSs. Immunohistochemistry showed the presence of the pre-synaptic protein synaptophysin and the post-synaptic protein drebrin (Figure 2E), suggesting that synaptic contacts are forming in HSs (Figure 2E).

quantified densitometrically and for quantitation of tau phosphorylation level, data were normalized by the level of actin (D). n = 3 independent differentiations per genotype. *$p < 0.05$. Statistical analysis by two-tailed t test.

(E–G) Characterization of synaptic proteins in APP variant, PS1 variant, and gender-matched control HSs. Confocal images showing representative examples of immunostaining for synaptic markers synaptophysin and drebrin in APP variant, PS1 variant, and gender-matched control HSs at DIV 100 and 3D surface reconstruction of dendritic segment with putative dendritic spines as delineated by drebrin labeling in close proximity to synaptophysin puncta in control HSs (E). Western blotting analysis of synaptic markers synaptophysin, drebrin, and PSD-95 in HSs at DIV 100 with MAP2d blot included as a loading control (F). The blots were quantified densitometrically and for quantitation of synaptic protein levels, data were normalized by the level of MAP2d (G). Results are presented as mean ± SEM. n = 3 independent differentiations per genotype. *$p < 0.05$; **$p < 0.01$. Statistical analysis by two-tailed t test. Scale bars, 100 μm (HS confocal images) and 5 μm (3D reconstruction images).

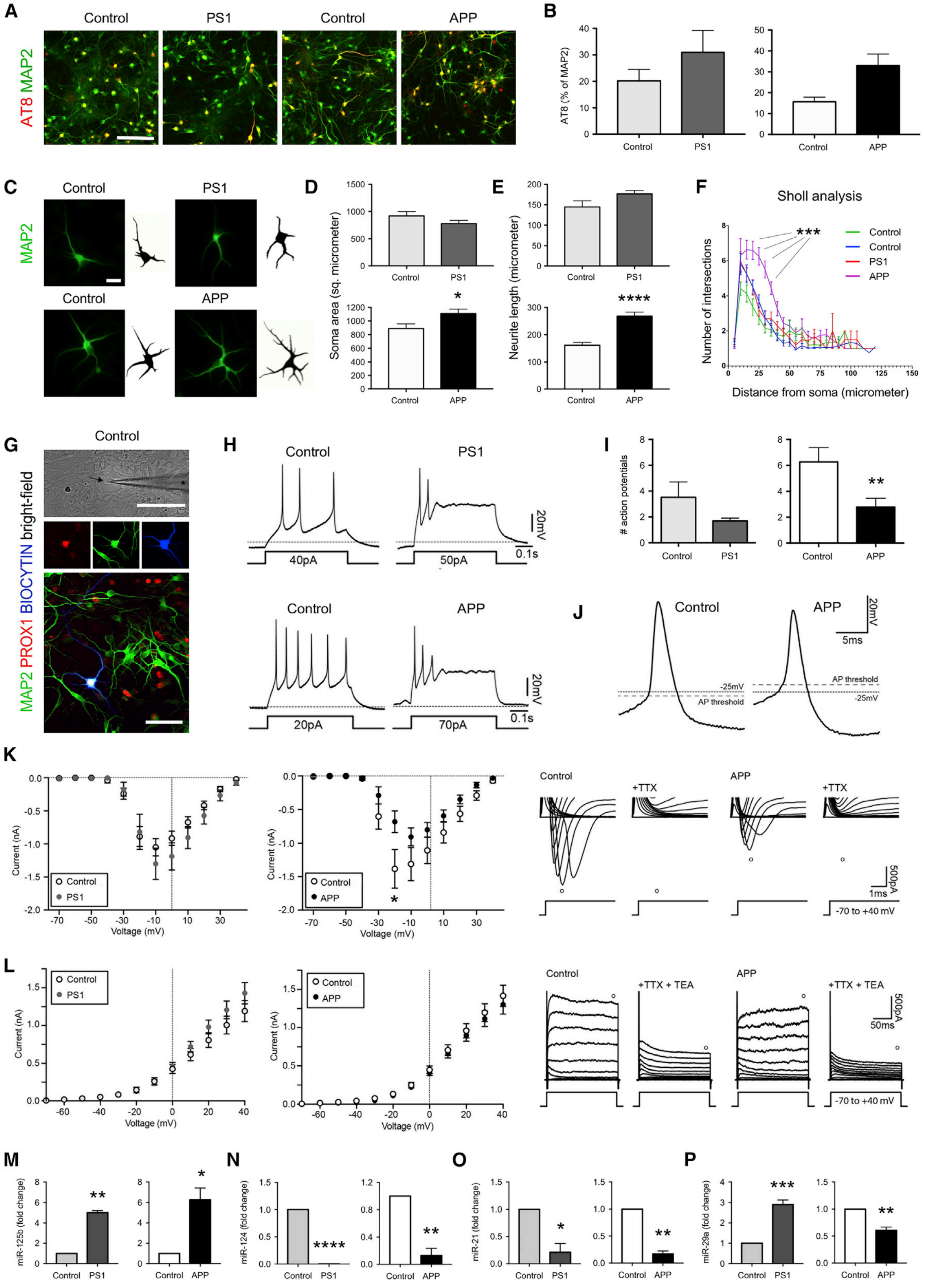

(legend on next page)

However, western blot analysis revealed that their abundance differed between AD and control samples, and levels of the two synaptic proteins were significantly decreased in both APP and PS1 variant HSs compared with their respective controls (Figures 2F and 2G). We also examined if there was a change in levels of the post-synaptic protein PSD-95 but found none (Figures 2F and 2G).

APP Variant Hippocampal Neurons Exhibit Several Significant Alterations

To gain insights into possible alterations of young AD hippocampal neurons, we examined phosphorylation of tau, the morphometric characteristics and electrophysiological properties of neurons, and miRNA levels of expression in cultures at 56 DIV. We found a higher proportion of AT8/MAP2-positive neurons in APP, but not in PS1 variant cultures as compared with controls (Figures 3A and 3B). Interestingly, the size of the soma, and the length and complexity of the neurites were altered only in APP variant neurons (Figures 3C–3F). Since morphometric characteristics can affect functional properties of neurons (Deshpande et al., 2017), we examined the functional differences between AD and healthy neurons using whole-cell patch-clamp recording (Figure 3G). Following somatic current injection, both AD and healthy hippocampal neurons were able to fire action potentials (APs) (Figure 3H). However, APP but not PS1 variant hippocampal neurons generated significantly fewer APs, when compared with control hippocampal neurons (Figures 3H and 3I). In addition, APs of APP variant neurons had a more depolarized threshold and lower amplitude (Figure 3G; Table S1). In line with these data, we observed that the amplitude of the TTX-sensitive inward sodium current was significantly reduced for APP but not PS1 variant hippocampal neurons when compared with controls (Figure 3K); whereas the outward, TEA-sensitive K current was unaltered for both APP and PS1 variant hippocampal neurons (Figure 3L).

Because miRNAs play an important role in AD pathogenesis (Miya Shaik et al., 2018), we next examined the levels of expression of several selected miRNAs previously identified to be dysregulated in AD models and patients. miRNA (miR)-125b was shown to be increased in the cerebrospinal fluid of AD patients (Dangla-Valls et al., 2017), and its overexpression causes tau hyperphosphorylation and impairs associative learning when injected into the hippocampus of mice (Banzhaf-Strathmann et al., 2014). We found miR-125b to be significantly upregulated in both APP and PS1 variant neural cultures (Figure 3M). Other microRNAs, such as miR-124, miR-21, and miR-29a/b were reported to be decreased in AD (An et al., 2017; Hebert et al., 2009; Leidinger et al., 2013; Lukiw, 2007; Shioya et al., 2010). Accordingly, we observed miR-124 and miR-21 downregulated in both APP and PS1 variant neuronal cultures (Figures 3N and 3O). Interestingly, miR29a, which is known

Figure 3. APP Variant Hippocampal Neurons Exhibit Significant Alterations

(A and B) Characterization of phosphorylation of tau protein in APP variant, PS1 variant, and gender-matched control hippocampal neurons. Immunostaining for phosphorylated tau protein in hippocampal neurons at DIV 56 (A). Quantification of phosphorylated (AT8-positive) neurons expressed relative to the total number of MAP2-positive neurons in hippocampal neurons at DIV 56 (B). Results are presented as mean ± SEM. n = 1–3 independent differentiations per clone for N = 3 iPSC clones per genotype. $^*p < 0.05$. Statistical analysis by two-tailed t test. Scale bar, 200 μm.

(C–F) Representative original fluorescence and converted binary images of APP variant, PS1 variant, and gender-matched control MAP2-positive hippocampal neurons at DIV 56 (C). Soma area (D), neurite length (E), and dendritic arborization (F) were quantified. Results are presented as mean ± SEM. n = 16–23 cells per line measured from 3 independent differentiations per genotype. $^*p < 0.05$, $^{***}p < 0.001$, $^{****}p < 0.0001$. Statistical analysis by two-tailed t test (D and E) and repeated measures ANOVA (F). Scale bar, 20 μm.

(G–L) Whole-cell patch-clamp recordings from APP variant, PS1 variant, and gender-matched control hippocampal neurons at DIV 56–58. Representative bright-field image of a patched control cell (arrow) along with the patch-clamp pipette (*) (G, upper panel) and fluorescence image of a patched PROX1-positive neuron filled with biocytin (G, lower panel). Voltage traces show the ability of hippocampal neurons to fire action potentials (APs) upon current injections (H). Bar diagrams show the maximal number of APs generated upon current injections (I). Expanded voltage traces of the first AP induced by a current ramp of 300 pA and used for determining the AP characteristics (J). Expanded current traces illustrating the inward sodium current (denoted by 0) activated during voltage steps ranging from −70 to +40 mV in 10-mV steps (K, right panel). The sodium current was blocked by the presence of 1 μM TTX. The current-voltage plots illustrate the sodium current peak plotted against the voltage steps (K, left panel). Current traces illustrating the outward potassium current (denoted by 0) activated during voltage steps ranging from −70 to +40 mV in 10-mV steps (L, right panel). The potassium current was inhibited by the addition of 10 mM TEA. The current-voltage plots illustrate the potassium current plotted against the voltage steps (L, left panel). Results are presented as mean ± SEM. n = 23–26 cells recorded from 3 independent differentiations per genotype. $^*p < 0.05$. Statistical analysis by Mann-Whitney test (I) and multiple t tests (K and L). Scale bars, 100 μm.

(M–P) Characterization of microRNA expression in APP variant, PS1 variant, and control hippocampal neurons. Bar diagrams showing the relative expression of miR-125b (M), miR-124 (N), miR-21 (O), and miR-29a (P) measured with RT-PCR in hippocampal neurons at DIV 56. Results are presented as mean ± SEM. n = 3 independent differentiations per genotype. $^*p < 0.05$; $^{**}p < 0.01$. Statistical analysis by two-tailed t test.

to be involved in the regulation of APP and β site APP cleaving enzyme 1 (BACE1) expression (Hebert et al., 2009), was downregulated in APP variant but upregulated in PS1 variant neural cultures (Figure 3P). This is in line with the reports showing that miR-29a could be differently expressed in AD brain (Cogswell et al., 2008; Hebert et al., 2008; Shioya et al., 2010).

APP Variant HSs Exhibit Increased Protein Aggregation

Both increased phosphorylation of tau in APP variant HSs and neurons (Figures 2C, 2D, 3A, and 3B) and increase in Aβ42/Aβ40 peptide ratios in both APP and PS1 variant HSs (Figure 2A) suggested that protein aggregation may be occurring in the patient hippocampal cells. To examine intracellular protein aggregation in the HSs, we used FTIR, a microspectroscopy-based imaging technique, as described previously (Klementieva et al., 2017). To better resolve the peak positions, we performed a second derivative analysis (de Aragao and Messaddeq, 2008). Strikingly, analysis of FTIR spectra revealed a significant increase in the content of β sheet structures in APP but not in PS1 variant HSs compared with controls (Figures 4A and 4B). This suggested that, although the Aβ42/Aβ40 ratio was increased in both APP and PS1 variant HSs, protein aggregation may be ongoing in the APP variant HSs only.

Since both neurons and astroglia were found in HSs, we investigated whether these two cell types could contribute to the increased protein conformational changes and aggregation in the AD variant HSs. We performed FTIR in neuronal cultures generated by dissociating HSs and culturing single cells as a monolayer until aged 56 DIV, and astroglial cultures generated by culturing HSs-derived progenitors as monolayers in medium containing ciliary neurotrophic factor until aged 100 DIV. We observed robust and significant increases in β sheet structures in APP hippocampal neurons. These data were confirmed when using a different platform and different iPSC clones (Figure S4). Interestingly, astrocytes derived from APP variant, but not from PS1 variant and control HSs (Figures 4A and 4B), displayed increases in β sheet structures. These data indicated that pathological changes also occur in AD astrocytes, as recently suggested (Oksanen et al., 2017). Importantly, we found no increase in β sheet structures in APP variant parental fibroblasts, demonstrating that protein aggregation is not present in the patient APP variant fibroblasts. This allowed us to rule out the possibility that pathological aggregation had been transferred from the APP variant fibroblasts to the iPSCs during reprogramming (Figures 4A and 4B).

Further assessment of protein aggregation by FTIR revealed elevation in the 1,513-cm^{-1} band, reflecting structural changes in tyrosine and tryptophan amino acids sensitive for phosphorylation (Barth et al., 1994), in APP, but not in PS1 variant HSs, neurons, and astrocytes (Figures 4A and 4C). Interestingly, significant elevation of the 1,513-cm^{-1} band was found in the two AD variants fibroblasts, although the level was low in PS1 variant fibroblasts (Figures 4A and 4C).

Quantitative Proteomic Analysis Reveals Important Alterations in the Proteome of APP and PS1 Variant HSs

To gain insights into cellular network alterations in APP and PS1 variants, we performed a quantitative assessment of the HS proteome, using label-free liquid chromatography-tandem mass spectrometry (LC-MS/MS). We identified over 6,000 proteins present in all 4 studied genotypes (Figure 5A) and could accurately quantify 98% of them (Table S2). Principal component analysis confirmed the HS segregation based on the gender (component 1) and the genotype (component 2) of the fibroblast donors (Figure 5B), hinting that gender-specific comparisons are important when analyzing disease phenotypes, as suggested previously in rodent studies (Richetin et al., 2017b). The largest sets of significantly dysregulated proteins were detected in APP variant HS (Table S2).

Subsequently, we performed pathway enrichment analysis of the identified dysregulated proteins, using the online bioinformatics tool DAVID. Notably, we found that proteins dysregulated in APP and PS1 variant HSs displayed a diversity of functions and were involved in several distinct pathways. For example, spliceosome, purine metabolism, and mRNA surveillance pathways were the highest of the significantly enriched pathways identified in APP variant HSs, where proteins were upregulated (Figure 5C). In contrast, in HSs harboring the PS1 variation, we identified DNA replication and DNA repair pathways as the most strongly enriched in upregulated proteins (Figure 5C). Similarly, we identified heterogeneity between the two AD variants when examining pathways enriched in downregulated proteins. Thus, in APP variant HSs, lysosomes, and phagosomes, endocytosis, oxidative phosphorylation, and protein processing in endoplasmic reticulum were the highly enriched pathways, among others, in downregulated proteins, whereas, in PS1 variant HSs, oxidative phosphorylation, endocytosis, axon guidance, lysosome and phagosome were the most strongly enriched pathways in downregulated proteins (Figure 5C).

Since we found decreased levels of synaptic proteins synaptophysin and drebrin by western blot (Figure 2F), we further examined synaptoproteomic alterations. Of all synaptic-related proteins analyzed, 26 were commonly downregulated in both APP and PS1 variant HSs (Figure 5D). Among them, we identified LIN7 proteins, which are important mediators of synaptic vesicle exocytosis (Butz

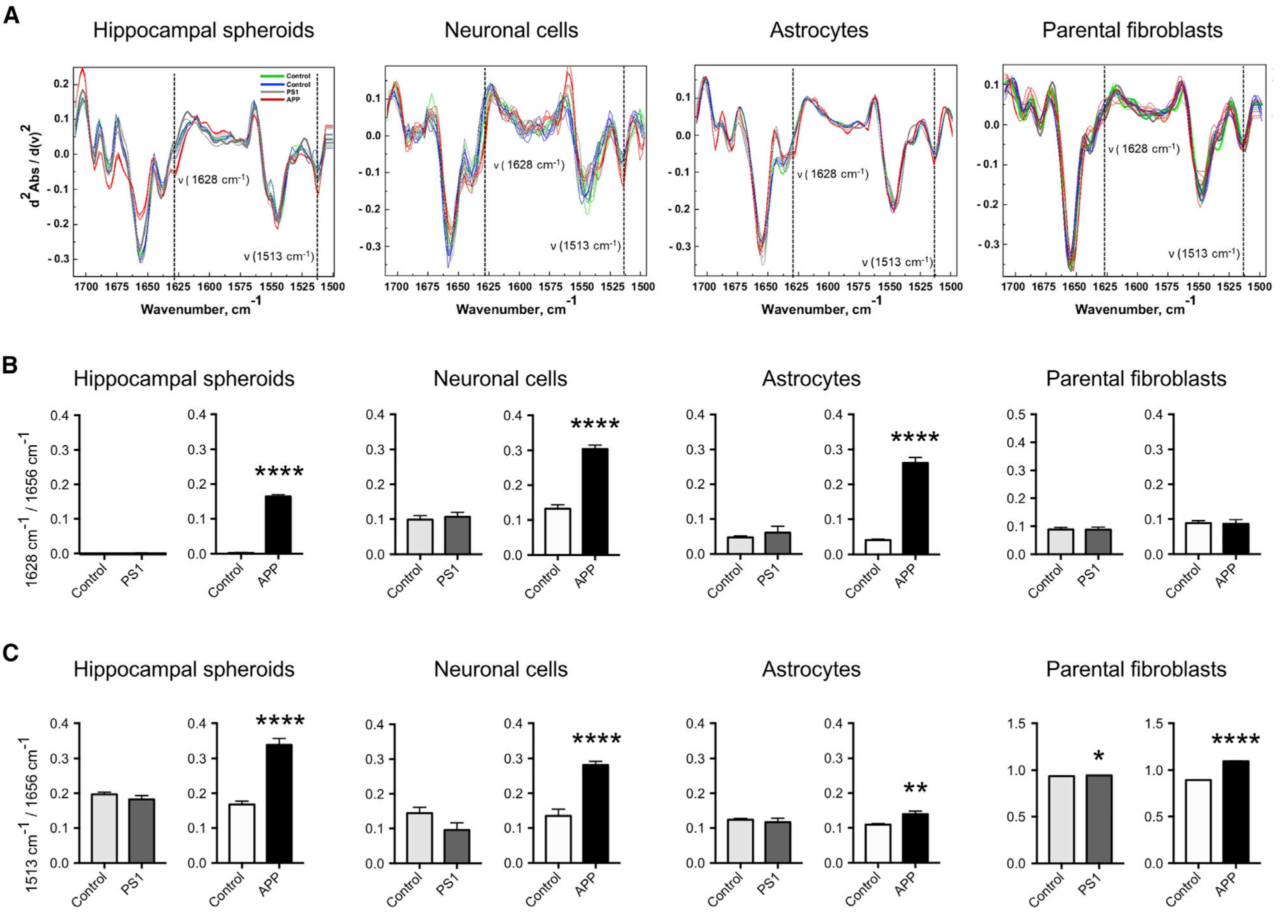

Figure 4. APP Variant HSs Exhibit Increased Protein Aggregation

(A) Normalized second derivatives of the infrared light absorbance spectra in Amide I and II regions. Dashed lines and arrows indicate the increased protein aggregation as elevation of β sheet structures (1,628 cm^{-1}) and structural changes corresponding to protein aggregation, as elevation at the band positioned at 1,513 cm^{-1} in APP in HSs at DIV 100, hippocampal neurons at DIV 56, astrocytes at DIV 120, and parental fibroblasts.

(B) Bar diagrams reflecting the β sheet structure content as shown by the absorbance ratios 1,628 to 1,656 cm^{-1} in the same cells described in (A). Results are presented as mean ± SEM. n = 5–10 spectra per genotype for HSs, n = 20–26 spectra per genotype for parental fibroblasts, n = 3 independent differentiations per genotype for hippocampal neurons and astrocytes. *$p < 0.05$, **$p < 0.01$, ****$p < 0.0001$. Statistical analysis by two-tailed t test.

(C) Bar diagrams reflecting structural changes as shown by the absorbance ratios 1,513 to 1,656 cm^{-1} in same cells described in (A). Results are presented as mean ± SEM. n = 5–10 spectra per one genotype for HSs. n = 20–26 spectra per genotype for parental fibroblasts, n = 3 independent differentiations per genotype for hippocampal neurons and astrocytes. *$p < 0.05$, **$p < 0.01$, ****$p < 0.0001$. Statistical analysis by two-tailed t test.

et al., 1998). Interestingly, an equal number of 90 different synaptic proteins were found specifically downregulated in APP or PS1 variant HSs. Gene ontology enrichment analysis revealed that these proteins were associated with biological processes centered around synaptic function, and included synaptic transmission, neurotransmitter secretion, and vesicle-mediated transport (Figures 5E and 5F; Table S3).

Finally, quantitative LC-MS/MS revealed alterations in several AD-related pathways (Figure 5G). Notably, specifically in APP variant HSs, we observed downregulation of mitochondrial proteins involved in oxidative phosphorylation, particularly complex IV and 3-hydroxyacyl-CoA dehydrogenase (HSD17B10), supporting the hypothesis of mitochondrial dysfunction in AD (He et al., 2018). We found a decrease in the levels of APOE, the major genetic risk factor for developing late-onset AD (Kim et al., 2009), in the APP variant HSs. Other groups of proteins were found downregulated in both variant samples, although this was more profound with the APP variant, e.g., proteins

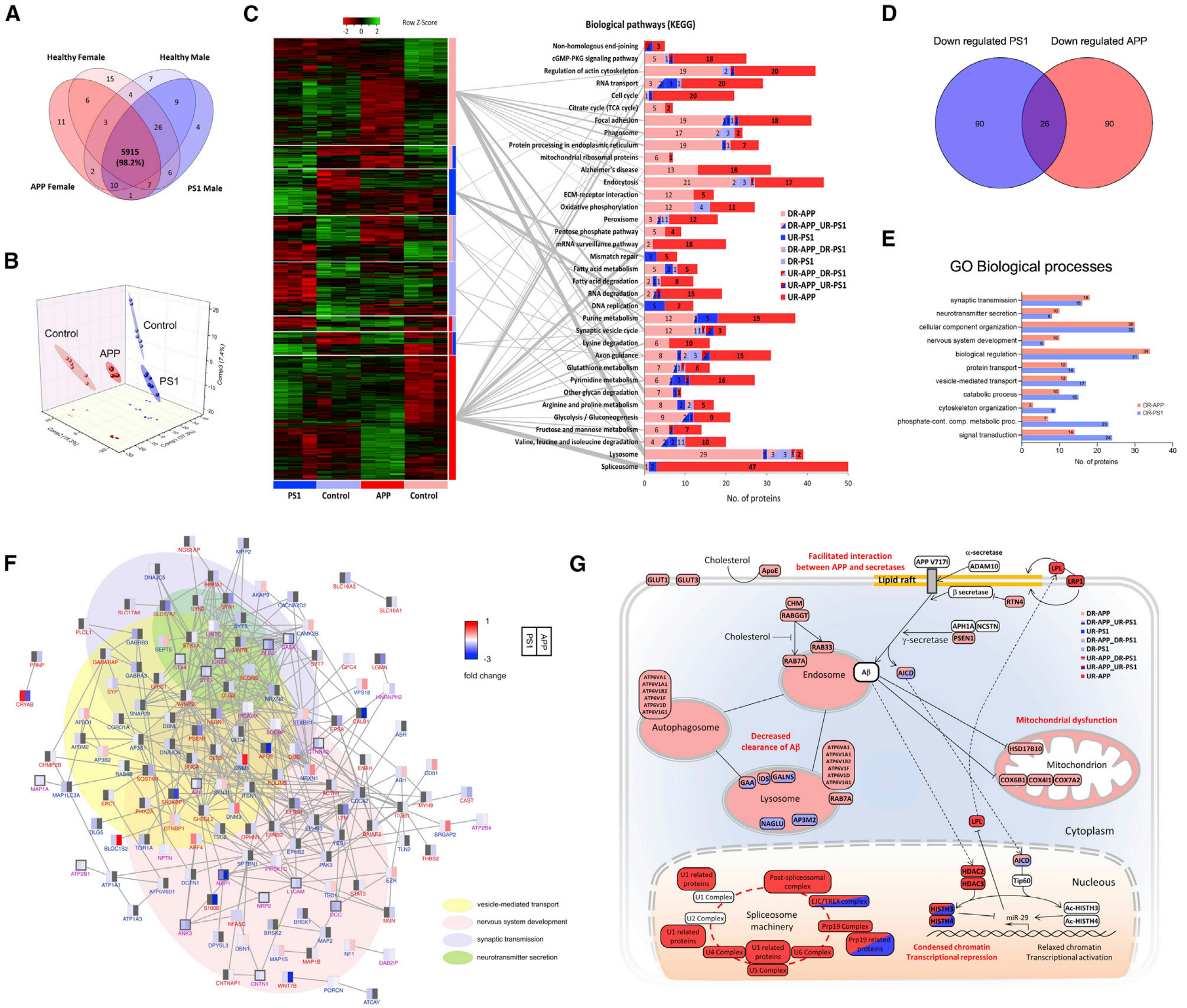

Figure 5. Label-free Quantitative Proteomics Reveals Important Alterations in the Proteome of APP and PS1 Variant HSs

(A) Venn diagram representing the number of proteins identified in HSs. The numbers correspond to the set of three different processes of generating HSs (biological replicates) and two technical replicates of the LC-MS/MS analysis.

(B) Principal component analysis using the normalized intensities of proteins identified/quantified in all samples.

(C) Heatmap of the protein relative abundances that were significantly dysregulated in at least one of the variant samples (left). n = 3 independent differentiations per genotype, n = 2 LC-MS/MS analyses. False discovery rate (FDR) < 0.05 was considered significant. Statistical analysis by two-tailed t test. Biological pathways (Kyoto Encyclopedia of Genes and Genomes) enriched in at least one of the eight blocks of dysregulated proteins (right). The graph shows the distribution of the proteins involved in each biological pathway by blocks of dysregulated proteins. Gray lines connect each block of proteins with their most enriched pathways.

(D) Venn diagram representing the number of synaptic proteins significantly downregulated in PS1 and APP variants when compared with their gender controls. n = 3 independent differentiations per genotype, n = 2 LC-MS/MS analyses. $p < 0.05$ with log2 fold change < -0.2 was considered significant. Statistical analysis by two-tailed t test.

(E) Representative enriched biological processes (Panther GO-Slim) for the downregulated synaptic proteins in PS1 and APP variants are shown with the number of corresponding proteins.

(F) Interaction network of the synaptic proteins downregulated in at least one of the samples carrying a mutation (n = 206). Each protein is represented by a rectangle divided into two parts (left-right), where different colors specifying the corresponding fold changes in PS1 and APP variants compared with their gender controls. Lines between the nodes (rectangles) indicate protein-protein interactions. Nodes with dark gray border represent synaptic proteins that are significantly downregulated in each variant. Purple label indicates significant

(legend continued on next page)

responsible for clearance of Aβ peptide. For example, the lysosome and the phagosome were the most enriched pathways among the downregulated ones in the samples carrying the APP variation. Among the most upregulated proteins, the components of the spliceosome machinery, including almost all the complexes in the pathway, were upregulated in both APP and PS1 variant samples. Interestingly, two proteins belonging to the histone deacetylase class 1 group (HDAC2 and HDAC3), which are known as transcriptional repressors (Yamakawa et al., 2017), were upregulated only in APP variant HSs. Similarly, the proteins lipoprotein lipase and low-density lipoprotein receptor-related protein 1, which were previously found to be overexpressed in AD (Baum et al., 1999), were upregulated in the APP variant HS sample.

ND1 *Ex Vivo* Gene Therapy Modulates the Transcription of Genes Relevant to AD Pathogenesis

We previously demonstrated that viral delivery of single transcription factor ND1 is sufficient to increase the maturation of rodent neural progenitors into neurons (Roybon et al., 2009a, 2009b, 2015), and reverse synaptic dysfunction and restore spatial memory in a rodent model of AD (Richetin et al., 2015, 2017a). To evaluate the potential of human HSs to serve as a translational platform for the development and/or testing of therapies for AD, we examined whether viral delivery of ND1 was sufficient to modulate the expression of genes differentially expressed in AD patient HS neurons compared with control.

We used Affymetrix GeneChip microarray to examine early transcriptomic changes in APP variant cells compared with control, upon viral delivery of ND1. We detected 377 genes whose expression was changed in APP variant cells compared with control (Figure 6A; Table S4). While the majority of genes (282), products of which participate in various biological processes, were downregulated in purified APP variant GFP-positive cells compared with control GFP-positive cells, 95 genes were upregulated, including genes encoding various GABA receptors, expression of which is altered in the early stages of AD (Saura et al., 2015).

Following transduction, ND1 expression was robustly identified (Figure 6B). Importantly, principal component analysis showed that ND1 overexpression changed the transcriptomic profile of APP variant GFP-positive cells, from a diseased state to an almost healthy control one (Figure 6C). In particular, ND1 positively modulated the expression of genes involved in synaptic transmission (Figures 6D–6F; and Table S5).

DISCUSSION

Here, we report an innovative method to chemically induce HSs from human iPSCs, which we used in a proof-of-concept study to model two patients carrying typical and atypical AD-related gene variations. We show that exposure of human iPSCs from different genders (male or female) and molecular diagnosis (diseased or healthy) to chemical agents LDN-193189, SB-431542, XAV-939, cyclopamine, and CHIR-99021 is sufficient to generate HSs containing LEF1-positive medial pallium progenitors, which when matured further formed hippocampal neural cells at different stages of maturation. While this original protocol produces approximately the same percentage of cells positive for ZBTB20 and PROX1, as recently reported by Sakaguchi et al. (2015) using human embryonic stem cells, it is more rapid and does not require long-term attachment culture. It also does not require iPSC-derived hippocampal neural progenitors to be plated on a monolayer of primary hippocampal astrocytes to generate mature neurons (Yu et al., 2014). Although HSs contain functional neurons, they were almost devoid of astrocytes. This is a significant drawback as astrocytes recently emerged as a new important target of AD (Jones et al., 2017; Konttinen et al., 2019b; Oksanen et al., 2017). Hence, to examine protein aggregation in astrocytes, we had to modify our protocol to generate astrocytic monolayer cultures. Since during human development astrocytes are generated later than neurons, we may assume that the number of astrocytes in the HSs may increase over time. With the constant improvement of protocols aiming at generating oligodendrocytes from human iPSCs (Djelloul et al., 2015; Madhavan et al., 2018), it would be valuable to also examine AD pathology in this cell type in future studies, since white matter myelin loss occurs in the AD patient brain (Mitew et al., 2010; Nasrabady et al., 2018). Cellular pathology was recently examined in iPSC-derived microglia (Konttinen et al., 2019a), a cell type that plays an important role in AD pathology (Venegas et al., 2017).

We utilized 3D hippocampal structures generated from iPSCs, to examine cellular dysfunction in typical and atypical familial AD. We used several techniques, from routine (western blotting, immunocytochemistry, qRT-PCR, MSD multi-array, and Affymetrix GeneChip microarray) to more sophisticated (whole-cell patch-clamp recording, synchrotron-based FTIR, and quantitative LC-MS/MS) and identified a wide variety of disease phenotypes. Some of them were reported previously when analyzing

downregulation in both variant, while blue and red labels indicate significant downregulation in PS1 or APP variant, respectively. n = 3 independent differentiations per genotype, n = 2 LC-MS/MS analyses. $p < 0.05$ with log2 fold change <-0.2 was considered significant. Statistical analysis by two-tailed t test.

(G) Schematic summary of AD-related dysregulated pathways and proteins in APP and PS1 variant HSs revealed by quantitative proteomics.

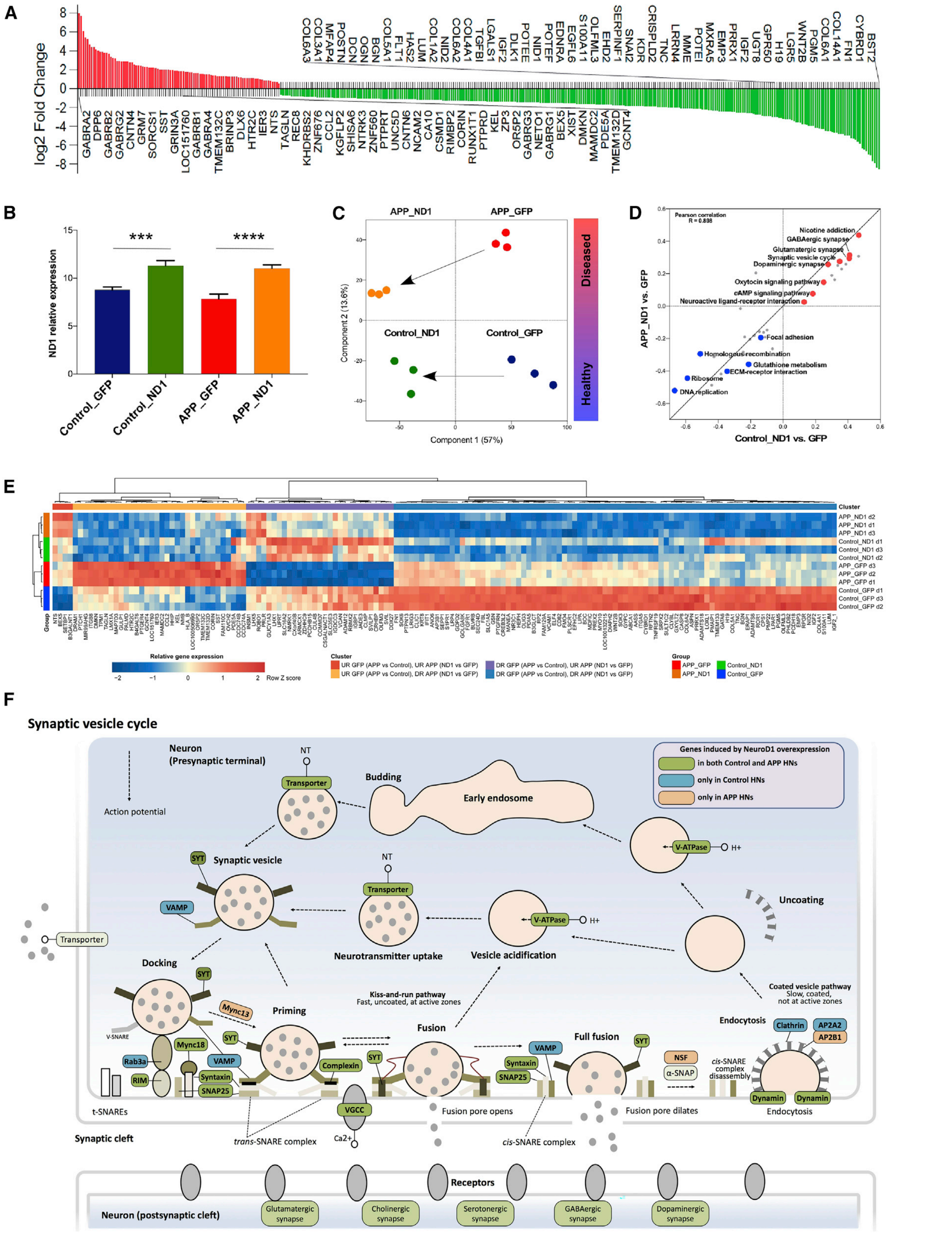

(legend on next page)

postmortem tissue, e.g., increased ratio of Aβ42/Aβ40 peptides, which can be neurotoxic (Kuperstein et al., 2010), and decreased levels of synaptic proteins levels (de Wilde et al., 2016), which are well-known cellular hallmarks of AD. Importantly, we identified cellular alterations that varied between the two AD variants, as also reported by others (Israel et al., 2012; Kondo et al., 2013). For example, we provide evidence of a significant increase in the content of β sheet structures and in the level of tau phosphorylation in APP but not in PS1 variant HSs compared with the controls. The difference of disease phenotypes, which was more prominent, or speculatively more advanced, in the APP variant when compared with the PS1 variant, could potentially be attributed to the fact that this patient carried a homozygous variation p.V717I in the APP gene (Sorbi et al., 1993), which could promote a stronger cellular pathology than if it was in a heterozygous form (Kondo et al., 2013; Ovchinnikov et al., 2018; Woodruff et al., 2016). Alternatively, it is possible that the PS1 variant displayed milder cellular phenotypes due to the nature of the variation. Indeed, patients carrying this PS1 p.R278K variation show a wide clinical spectrum, even between family members, from classical AD to pure spastic paraparesis (Assini et al., 2003). In this particular case, spastic paraparesis was the initial feature, which we speculate might explain the lesser phenotypic changes (e.g., protein aggregation) in HSs from the PS1 compared with APP variants. The correlation between patients' symptoms and cellular pathogenesis was demonstrated by several groups, including Woodard et al. (2014) who studied iPSC-derived dopaminergic neurons generated from GBA mutant Parkinsonian monozygotic twins clinically discordant in their symptoms: the iPSC-derived dopaminergic neurons generated from the symptomatic twin exhibited strong cellular pathogenesis, as opposed to those generated from the asymptomatic brother. In addition, the wide variety and heterogeneity of the phenotypes identified could be influenced by factors that exacerbate or alleviate them.

One limitation of this study is the number of patients used. This is due to the uniqueness of the variations they carry: homozygous APP London variation is rare (Sorbi et al., 1993); and PS1 p.R278K variation was only reported in one family (Assini et al., 2003). To minimize this limitation, we used several independent iPSC clones per individual, to examine the efficacy of our protocol and the main readout assays (tau phosphorylation and protein aggregation). Nevertheless, HSs allowed us to identify early disease phenotypes consistent across the different iPSC clones studied, which is not possible with current imaging methods, such as positron electron tomography or magnetic resonance imaging.

Our work also raises the question whether AD is a neurodevelopmental disorder? AD is considered, such as Parkinson disease and several other common neurodegenerative disorders, as a disease of the aging brain (Wyss-Coray, 2016). Early disease cellular phenotypes can be identified using pre- and post-natal rodent brain cells (Almeida et al., 2005; Nagai et al., 2007), while disease onset declares itself only after several months in the rodent models; and early changes can be observed when using advanced techniques, such as FTIR (Klementieva et al., 2017). This raises even more attention when modeling diseases using iPSCs. Indeed, even if iPSCs are non-natural cell types generated by the reprogramming of (often) aged somatic cells (Dimos et al., 2008), they are embryonic-like in their nature (Ho et al., 2016; Zhao et al., 2018). Over the last decade, experimental work using iPSCs suggests that early disease phenotypes can be identified as soon as a few weeks to few months *in vitro* for several neurodegenerative disorders (Fujimori et al., 2018; Guo et al., 2017; Muratore et al., 2014; Woodard et al., 2014). Moreover, recent work suggested that early therapeutic intervention could alleviate early behavioral, cellular, and molecular changes seen in Huntington disease (HD) (Siebzehnrubl et al., 2018), and disease phenotypes have been reversed in neuronal cells generated from iPSCs of HD patients (Consortium, 2017). This raises the

Figure 6. ND1 *Ex Vivo* Gene Therapy Modulates Transcription of Genes Relevant to AD

(A) Genes dysregulated in APP variant hippocampal neurons at DIV 50. The top 50 genes upregulated (in red) and downregulated (in green) are annotated. n = 3 independent differentiations per genotype. FDR < 0.05 was considered significant. Statistical analysis by two-tailed t test.

(B) Relative expression levels of ND1 gene. Results are presented as mean ± S.D. n = 3 independent differentiations per genotype. ***p < 0.001, ****p < 0.0001. Statistical analysis by one-way ANOVA followed by Tukey's *post hoc* test.

(C) Principal component analysis using gene expression profiles of hippocampal neurons.

(D) 2D annotation enrichment analysis for biological pathways based on the differential expression profiles of transcripts between hippocampal neurons transduced with ND1 and GFP, in both control (x axis) and APP variant (y axis). Pathways significantly enriched after ND1 overexpression are highlighted, downregulated (blue) and upregulated (red). n = 3 independent differentiations per genotype. Enriched pathways were selected according to a Benjamini-Hochberg FDR of 0.01 (Table S5).

(E) Heatmap of the relative expression of genes that were significantly dysregulated in APP variant hippocampal neurons and modulated by ND1 overexpression (Table S5). n = 3 independent differentiations per genotype. Statistical analysis by two-tailed t test.

(F) Schematic representation of synaptic vesicle cycle pathway in hippocampal neurons. Genes whose expression was induced by ND1 overexpression are highlighted.

possibility that early pharmacological intervention in AD (and other neurodegenerative disorders) might also be valuable. Toward this aim, we evaluated the effect of ND1 on human HSs-derived neuronal progenitors. This strategy was previously used with *in vitro* and *in vivo* systems, and it proved to be beneficial in rodent models of AD by reversing memory impairment (Richetin et al., 2015, 2017a). We found that ND1 positively modulated the expression of genes involved in synaptic transmission (Figures 6D–6F). This is important since synaptic transmission impairment is a hallmark of AD pathology (de Wilde et al., 2016), and ND1 overexpression is sufficient to stimulate synaptic connectivity and excitability of new adult hippocampal neurons (Richetin et al., 2015, 2017a). This proof-of-concept study further showed that HSs could serve as a platform for developing therapeutic strategies and evaluate their mechanism of action at the cellular level.

In conclusion, iPSC-derived HSs provide a complementary technology to 2D human hippocampal monolayers developed recently (Sarkar et al., 2018; Yu et al., 2014), and offer the possibility to examine early pathological changes in a minimalist human AD hippocampal parenchyma-like model, although devoid of oligodendrocytes, microglia, and vasculature. HSs can further be grafted into immunodeficient mice, either as single cells, as we did, or as a whole (Mansour et al., 2018), which can further offer an invaluable tool to examine how cellular pathology develops, as well as the effects of potential drugs on human diseased cells, *in vivo*, over time.

EXPERIMENTAL PROCEDURES

All reagents and procedures details can be found in Supplemental Experimental Procedures.

Generation of iPSCs

Primary human dermal fibroblasts were harvested by punch skin biopsy from AD patients and healthy donors after written informed consent. Reprogramming factors (OCT-3/4, KLF-4, SOX-2, and c-MYC) were delivered using a non-integrating Sendai virus vectors kit (Thermo Fisher Scientific). A month later, several colonies were collected and expanded as single clones for 7 days. Two to three iPSC clones per individuals were selected for further expansion and characterization (Figure S1). The reprogramming of patient cells into iPSC was approved by the Swedish work environment authority. An additional female control line (TALSCTRL15.12) was received from Target ALS (http://www.targetals.org/) and NHCDR Repositories hosted by RUCDR. Work was carried out according to European and Swedish national rules, with the highest level of ethics.

Differentiation of iPSCs to HSs

Human iPSC colonies were dissociated using dispase and transferred into ultra-low adherent flasks (Corning) in WiCell medium supplemented with 20 ng/mL fibroblast growth factor 2 and 20 μM ROCK inhibitor Y-27632 (Selleck Chemicals, Munich, Germany). Next day, WiCell was replaced with neural induction medium composed of advanced DMEM/F12, 2% B27 Supplement without vitamin A (v/v), 1% N2 Supplement (v/v), 1% NEAA (v/v), 2 mM L-glutamine and 1% penicillin-streptomycin (v/v). For dorsomedial telencephalic neural specification, LDN-193189 (Stemgent, 0.1 μM), Cyclopamine (Selleck Chemicals, 1 μM), SB431542 (Sigma-Aldrich, 10 μM) and XAV-939 (Tocris, 5 μM) were added to the medium for the first 10 days with medium change every other day. On the 10th day, the free-floating spheres were transferred to neuronal differentiation medium (NDM) containing Neurobasal medium, 1% N2 (v/v), 1% NEAA (v/v), L-glutamine, and 1% penicillin-streptomycin (v/v). To promote hippocampal differentiation, NDM was supplemented with CHIR-99021 (Stemgent, 0.5 mM) and brain-derived neurotrophic factor (PeproTech, 20 ng/mL) for 90 days with medium changes every second day.

Data and Code Availability

The mass spectrometry proteomics data have been deposited to the ProteomeXchange Consortium via the PRIDE partner repository with the dataset identifier PXD012524. Affymetrix GeneChip microarray data are available in Table S4 and contain main information on the probes; the full dataset has been deposited to the Gene Expression Omnibus repository with the dataset identifier GSE149599. Omics data are also available at the Roybon laboratory website (https://www.ipsc-cns-disease.lu.se/ipsc-laboratory-for-cns-disease-modeling/resources), or via simple email request.

SUPPLEMENTAL INFORMATION

Supplemental Information can be found online at https://doi.org/10.1016/j.stemcr.2020.06.001.

AUTHOR CONTRIBUTIONS

Y.P. and L.R. conceived the experiments and wrote the manuscript; all authors performed or assisted with the experiments; and provided reagents, expertise, and conducted a critical analysis and review of the manuscript. All authors provided input during the editing of the manuscript and approved its content.

DECLARATION OF INTERESTS

The authors declare no competing financial interests.

ACKNOWLEDGMENTS

We are particularly thankful to Marianne Juhlin, Anna-Karin Olden, Janneke Lassche, and Evgenija Serafimova for their outstanding technical assistance. We are also thankful to the Galliera Genetic Bank (Galliera Hospital), member of the Network Telethon of Genetic Biobanks (project no. GTB12001), funded by Telethon Italy and the Eurobiobank Network, which provided us with the PS1 and control fibroblast specimens, and Target ALS (http://www.targetals.org/) and NHCDR Repositories hosted by RUCDR for providing the control iPSC cell line TALSCTRL15.12.

In addition, we are especially thankful to Thermo Fischer Scientific for instrumental support. We thank Professor Per Persson for the access to FTIR equipment. We acknowledge funding support from the strategic research areas MultiPark and Stem Therapy at Lund University, The Linnaeus environment Bagadilico, as well as the Joint Programme for Neurodegenerative Disease (JPND grant acronym MADGIC) research co-funded by the EU Research and Innovation Program Horizon 2020 through the ERA-NET co-fund scheme to L.R (VR no. 2015-07798), to D.B. (FCT: JPco-fuND/0003/2015) and to G.K.G. (VR no. 2015-06797). We are also thankful to the FCT grant UID/DTP/04138/2013 to iMed.ULisboa, and private donation grants to L.R. from the Swedish Alzheimer foundation (Alzheimerfonden), The Crafoord Foundation, The Åhlens Foundation, The Dementia Foundation Sweden (Demensfonden), and The Olle Engkvist Byggmästare Foundation. Y.P. was supported partly by the Finnish Cultural Foundation and Royal Physiographic Society in Lund, A.R.V was supported by the Fundação para a Ciência e a Tecnologia (FCT), Portugal (postdoctoral research fellowship [SFRH/BPD/76590/2011]), S.S. was supported by Fondi per la ricerca 2017, and Z.K. was supported by the Swedish Research Council and the Swedish Brain Foundation.

Received: August 13, 2019
Revised: June 1, 2020
Accepted: June 1, 2020
Published: June 25, 2020

REFERENCES

Abellan, A., Desfilis, E., and Medina, L. (2014). Combinatorial expression of Lef1, Lhx2, Lhx5, Lhx9, Lmo3, Lmo4, and Prox1 helps to identify comparable subdivisions in the developing hippocampal formation of mouse and chicken. Front. Neuroanat. *8*, 59.

Almeida, C.G., Tampellini, D., Takahashi, R.H., Greengard, P., Lin, M.T., Snyder, E.M., and Gouras, G.K. (2005). beta-Amyloid accumulation in APP mutant neurons reduces PSD-95 and GluR1 in synapses. Neurobiol. Dis. *20*, 187–198.

An, F., Gong, G., Wang, Y., Bian, M., Yu, L., and Wei, C. (2017). MiR-124 acts as a target for Alzheimer's disease by regulating BACE1. Oncotarget *8*, 114065–114071.

Arber, C., Lovejoy, C., and Wray, S. (2017). Stem cell models of Alzheimer's disease: progress and challenges. Alzheimers Res. Ther. *9*, 42.

Assini, A., Terreni, L., Borghi, R., Giliberto, L., Piccini, A., Loqui, D., Fogliarino, S., Forloni, G., and Tabaton, M. (2003). Pure spastic paraparesis associated with a novel presenilin 1 R278K mutation. Neurology *60*, 150.

Baker, M.J., Trevisan, J., Bassan, P., Bhargava, R., Butler, H.J., Dorling, K.M., Fielden, P.R., Fogarty, S.W., Fullwood, N.J., Heys, K.A., et al. (2014). Using Fourier transform IR spectroscopy to analyze biological materials. Nat. Protoc. *9*, 1771–1791.

Banzhaf-Strathmann, J., Benito, E., May, S., Arzberger, T., Tahirovic, S., Kretzschmar, H., Fischer, A., and Edbauer, D. (2014). MicroRNA-125b induces tau hyperphosphorylation and cognitive deficits in Alzheimer's disease. EMBO J. *33*, 1667–1680.

Barth, A., Kreutz, W., and Mantele, W. (1994). Changes of protein structure, nucleotide microenvironment, and Ca(2+)-binding states in the catalytic cycle of sarcoplasmic reticulum Ca(2+)-ATPase: investigation of nucleotide binding, phosphorylation and phosphoenzyme conversion by FTIR difference spectroscopy. Biochim. Biophys. Acta *1194*, 75–91.

Baum, L., Chen, L., Masliah, E., Chan, Y.S., Ng, H.K., and Pang, C.P. (1999). Lipoprotein lipase mutations and Alzheimer's disease. Am. J. Med. Genet. *88*, 136–139.

Boutet, C., Chupin, M., Lehericy, S., Marrakchi-Kacem, L., Epelbaum, S., Poupon, C., Wiggins, C., Vignaud, A., Hasboun, D., Defontaines, B., et al. (2014). Detection of volume loss in hippocampal layers in Alzheimer's disease using 7 T MRI: a feasibility study. Neuroimage Clin. *5*, 341–348.

Bull, N.D., and Bartlett, P.F. (2005). The adult mouse hippocampal progenitor is neurogenic but not a stem cell. J. Neurosci. *25*, 10815–10821.

Butz, S., Okamoto, M., and Sudhof, T.C. (1998). A tripartite protein complex with the potential to couple synaptic vesicle exocytosis to cell adhesion in brain. Cell *94*, 773–782.

Chambers, S.M., Fasano, C.A., Papapetrou, E.P., Tomishima, M., Sadelain, M., and Studer, L. (2009). Highly efficient neural conversion of human ES and iPS cells by dual inhibition of SMAD signaling. Nat. Biotechnol. *27*, 275–280.

Chen, J.K., Taipale, J., Cooper, M.K., and Beachy, P.A. (2002). Inhibition of Hedgehog signaling by direct binding of cyclopamine to Smoothened. Genes Dev. *16*, 2743–2748.

Clevers, H. (2016). Modeling development and disease with organoids. Cell *165*, 1586–1597.

Cogswell, J.P., Ward, J., Taylor, I.A., Waters, M., Shi, Y., Cannon, B., Kelnar, K., Kemppainen, J., Brown, D., Chen, C., et al. (2008). Identification of miRNA changes in Alzheimer's disease brain and CSF yields putative biomarkers and insights into disease pathways. J. Alzheimers Dis. *14*, 27–41.

Consortium, H.D.i. (2017). Developmental alterations in Huntington's disease neural cells and pharmacological rescue in cells and mice. Nat. Neurosci. *20*, 648–660.

Dangla-Valls, A., Molinuevo, J.L., Altirriba, J., Sanchez-Valle, R., Alcolea, D., Fortea, J., Rami, L., Balasa, M., Munoz-Garcia, C., Ezquerra, M., et al. (2017). CSF microRNA profiling in Alzheimer's disease: a screening and validation study. Mol. Neurobiol. *54*, 6647–6654.

de Aragao, B.J.G., and Messaddeq, Y. (2008). Peak separation by derivative spectroscopy applied to FTIR analysis of hydrolized silica. J. Brazil Chem. Soc. *19*, 1582–1594.

de Wilde, M.C., Overk, C.R., Sijben, J.W., and Masliah, E. (2016). Meta-analysis of synaptic pathology in Alzheimer's disease reveals selective molecular vesicular machinery vulnerability. Alzheimers Dement. *12*, 633–644.

Deshpande, A., Yadav, S., Dao, D.Q., Wu, Z.Y., Hokanson, K.C., Cahill, M.K., Wiita, A.P., Jan, Y.N., Ullian, E.M., and Weiss, L.A. (2017). Cellular phenotypes in human iPSC-derived neurons from a genetic model of autism spectrum disorder. Cell Rep. *21*, 2678–2687.

Dimos, J.T., Rodolfa, K.T., Niakan, K.K., Weisenthal, L.M., Mitsumoto, H., Chung, W., Croft, G.F., Saphier, G., Leibel, R., Goland, R., et al. (2008). Induced pluripotent stem cells generated from patients with ALS can be differentiated into motor neurons. Science *321*, 1218–1221.

Djelloul, M., Holmqvist, S., Boza-Serrano, A., Azevedo, C., Yeung, M.S., Goldwurm, S., Frisen, J., Deierborg, T., and Roybon, L. (2015). Alpha-synuclein expression in the oligodendrocyte lineage: an in vitro and in vivo study using rodent and human models. Stem Cell Reports *5*, 174–184.

Duering, M., Grimm, M.O., Grimm, H.S., Schroder, J., and Hartmann, T. (2005). Mean age of onset in familial Alzheimer's disease is determined by amyloid beta 42. Neurobiol. Aging *26*, 785–788.

Forner, S., Baglietto-Vargas, D., Martini, A.C., Trujillo-Estrada, L., and LaFerla, F.M. (2017). Synaptic impairment in Alzheimer's disease: a dysregulated symphony. Trends Neurosci. *40*, 347–357.

Fujimori, K., Ishikawa, M., Otomo, A., Atsuta, N., Nakamura, R., Akiyama, T., Hadano, S., Aoki, M., Saya, H., Sobue, G., et al. (2018). Modeling sporadic ALS in iPSC-derived motor neurons identifies a potential therapeutic agent. Nat. Med. *24*, 1579–1589.

Goedert, M., Jakes, R., and Vanmechelen, E. (1995). Monoclonal antibody AT8 recognises tau protein phosphorylated at both serine 202 and threonine 205. Neurosci. Lett. *189*, 167–169.

Guo, W., Naujock, M., Fumagalli, L., Vandoorne, T., Baatsen, P., Boon, R., Ordovas, L., Patel, A., Welters, M., Vanwelden, T., et al. (2017). HDAC6 inhibition reverses axonal transport defects in motor neurons derived from FUS-ALS patients. Nat. Commun. *8*, 861.

He, X.Y., Isaacs, C., and Yang, S.Y. (2018). Roles of mitochondrial 17beta-hydroxysteroid dehydrogenase type 10 in Alzheimer's disease. J. Alzheimers Dis. *62*, 665–673.

Hebert, S.S., Horre, K., Nicolai, L., Bergmans, B., Papadopoulou, A.S., Delacourte, A., and De Strooper, B. (2009). MicroRNA regulation of Alzheimer's amyloid precursor protein expression. Neurobiol. Dis. *33*, 422–428.

Hebert, S.S., Horre, K., Nicolai, L., Papadopoulou, A.S., Mandemakers, W., Silahtaroglu, A.N., Kauppinen, S., Delacourte, A., and De Strooper, B. (2008). Loss of microRNA cluster miR-29a/b-1 in sporadic Alzheimer's disease correlates with increased BACE1/beta-secretase expression. Proc. Natl. Acad. Sci. U S A *105*, 6415–6420.

Ho, R., Sances, S., Gowing, G., Amoroso, M.W., O'Rourke, J.G., Sahabian, A., Wichterle, H., Baloh, R.H., Sareen, D., and Svendsen, C.N. (2016). ALS disrupts spinal motor neuron maturation and aging pathways within gene co-expression networks. Nat. Neurosci. *19*, 1256–1267.

Holmqvist, S., Lehtonen, S., Chumarina, M., Puttonen, K.A., Azevedo, C., Lebedeva, O., Ruponen, M., Oksanen, M., Djelloul, M., Collin, A., et al. (2016). Creation of a library of induced pluripotent stem cells from Parkinsonian patients. NPJ Parkinsons Dis. *2*, 16009.

Israel, M.A., Yuan, S.H., Bardy, C., Reyna, S.M., Mu, Y., Herrera, C., Hefferan, M.P., Van Gorp, S., Nazor, K.L., Boscolo, F.S., et al. (2012). Probing sporadic and familial Alzheimer's disease using induced pluripotent stem cells. Nature *482*, 216–220.

Jin, M., Shepardson, N., Yang, T., Chen, G., Walsh, D., and Selkoe, D.J. (2011). Soluble amyloid beta-protein dimers isolated from Alzheimer cortex directly induce Tau hyperphosphorylation and neuritic degeneration. Proc. Natl. Acad. Sci. U S A *108*, 5819–5824.

Jones, V.C., Atkinson-Dell, R., Verkhratsky, A., and Mohamet, L. (2017). Aberrant iPSC-derived human astrocytes in Alzheimer's disease. Cell Death Dis. *8*, e2696.

Kim, J., Basak, J.M., and Holtzman, D.M. (2009). The role of apolipoprotein E in Alzheimer's disease. Neuron *63*, 287–303.

Klementieva, O., Willen, K., Martinsson, I., Israelsson, B., Engdahl, A., Cladera, J., Uvdal, P., and Gouras, G.K. (2017). Pre-plaque conformational changes in Alzheimer's disease-linked Abeta and APP. Nat. Commun. *8*, 14726.

Kondo, T., Asai, M., Tsukita, K., Kutoku, Y., Ohsawa, Y., Sunada, Y., Imamura, K., Egawa, N., Yahata, N., Okita, K., et al. (2013). Modeling Alzheimer's disease with iPSCs reveals stress phenotypes associated with intracellular Abeta and differential drug responsiveness. Cell Stem Cell *12*, 487–496.

Konttinen, H., Cabral-da-Silva, M.E.C., Ohtonen, S., Wojciechowski, S., Shakirzyanova, A., Caligola, S., Giugno, R., Ishchenko, Y., Hernandez, D., Fazaludeen, M.F., et al. (2019a). PSEN1DeltaE9, APPswe, and APOE4 confer disparate phenotypes in human iPSC-derived microglia. Stem Cell Reports *13*, 669–683.

Konttinen, H., Gureviciene, I., Oksanen, M., Grubman, A., Loppi, S., Huuskonen, M.T., Korhonen, P., Lampinen, R., Keuters, M., Belaya, I., et al. (2019b). PPARbeta/delta-agonist GW0742 ameliorates dysfunction in fatty acid oxidation in PSEN1DeltaE9 astrocytes. Glia *67*, 146–159.

Kuperstein, I., Broersen, K., Benilova, I., Rozenski, J., Jonckheere, W., Debulpaep, M., Vandersteen, A., Segers-Nolten, I., Van Der Werf, K., Subramaniam, V., et al. (2010). Neurotoxicity of Alzheimer's disease Abeta peptides is induced by small changes in the Abeta42 to Abeta40 ratio. EMBO J. *29*, 3408–3420.

Lavado, A., and Oliver, G. (2007). Prox1 expression patterns in the developing and adult murine brain. Dev. Dyn. *236*, 518–524.

Lee, S.M., Tole, S., Grove, E., and McMahon, A.P. (2000). A local Wnt-3a signal is required for development of the mammalian hippocampus. Development *127*, 457–467.

Leidinger, P., Backes, C., Deutscher, S., Schmitt, K., Mueller, S.C., Frese, K., Haas, J., Ruprecht, K., Paul, F., Stahler, C., et al. (2013). A blood based 12-miRNA signature of Alzheimer disease patients. Genome Biol. *14*, R78.

Lukiw, W.J. (2007). Micro-RNA speciation in fetal, adult and Alzheimer's disease hippocampus. Neuroreport *18*, 297–300.

Madhavan, M., Nevin, Z.S., Shick, H.E., Garrison, E., Clarkson-Paredes, C., Karl, M., Clayton, B.L.L., Factor, D.C., Allan, K.C., Barbar, L., et al. (2018). Induction of myelinating oligodendrocytes in human cortical spheroids. Nat. Methods *15*, 700–706.

Mandelkow, E.M., and Mandelkow, E. (2012). Biochemistry and cell biology of tau protein in neurofibrillary degeneration. Cold Spring Harb. Perspect. Med. *2*, a006247.

Mansour, A.A., Goncalves, J.T., Bloyd, C.W., Li, H., Fernandes, S., Quang, D., Johnston, S., Parylak, S.L., Jin, X., and Gage, F.H. (2018). An in vivo model of functional and vascularized human brain organoids. Nat. Biotechnol. *36*, 432–441.

Mitew, S., Kirkcaldie, M.T., Halliday, G.M., Shepherd, C.E., Vickers, J.C., and Dickson, T.C. (2010). Focal demyelination in Alzheimer's disease and transgenic mouse models. Acta Neuropathol. *119*, 567–577.

Miya Shaik, M., Tamargo, I.A., Abubakar, M.B., Kamal, M.A., Greig, N.H., and Gan, S.H. (2018). The role of microRNAs in Alzheimer's disease and their therapeutic potentials. Genes (Basel) *9*. https://doi.org/10.3390/genes9040174.

Morris, G.P., Clark, I.A., and Vissel, B. (2014). Inconsistencies and controversies surrounding the amyloid hypothesis of Alzheimer's disease. Acta Neuropathol. Commun. *2*, 135.

Mueller, S.G., Schuff, N., Yaffe, K., Madison, C., Miller, B., and Weiner, M.W. (2010). Hippocampal atrophy patterns in mild cognitive impairment and Alzheimer's disease. Hum. Brain Mapp. *31*, 1339–1347.

Muratore, C.R., Rice, H.C., Srikanth, P., Callahan, D.G., Shin, T., Benjamin, L.N., Walsh, D.M., Selkoe, D.J., and Young-Pearse, T.L. (2014). The familial Alzheimer's disease APPV717I mutation alters APP processing and Tau expression in iPSC-derived neurons. Hum. Mol. Genet. *23*, 3523–3536.

Murphy, M.P., and LeVine, H., 3rd. (2010). Alzheimer's disease and the amyloid-beta peptide. J. Alzheimers Dis. *19*, 311–323.

Nagai, M., Re, D.B., Nagata, T., Chalazonitis, A., Jessell, T.M., Wichterle, H., and Przedborski, S. (2007). Astrocytes expressing ALS-linked mutated SOD1 release factors selectively toxic to motor neurons. Nat. Neurosci. *10*, 615–622.

Nasrabady, S.E., Rizvi, B., Goldman, J.E., and Brickman, A.M. (2018). White matter changes in Alzheimer's disease: a focus on myelin and oligodendrocytes. Acta Neuropathol. Commun. *6*, 22.

Nicoleau, C., Varela, C., Bonnefond, C., Maury, Y., Bugi, A., Aubry, L., Viegas, P., Bourgois-Rocha, F., Peschanski, M., and Perrier, A.L. (2013). Embryonic stem cells neural differentiation qualifies the role of Wnt/beta-Catenin signals in human telencephalic specification and regionalization. Stem Cells *31*, 1763–1774.

Nielsen, J.V., Thomassen, M., Mollgard, K., Noraberg, J., and Jensen, N.A. (2014). Zbtb20 defines a hippocampal neuronal identity through direct repression of genes that control projection neuron development in the isocortex. Cereb. Cortex *24*, 1216–1229.

Oksanen, M., Petersen, A.J., Naumenko, N., Puttonen, K., Lehtonen, S., Gubert Olive, M., Shakirzyanova, A., Leskela, S., Sarajarvi, T., Viitanen, M., et al. (2017). PSEN1 mutant iPSC-derived model reveals severe astrocyte pathology in Alzheimer's disease. Stem Cell Reports *9*, 1885–1897.

Ovchinnikov, D.A., Korn, O., Virshup, I., Wells, C.A., and Wolvetang, E.J. (2018). The impact of APP on Alzheimer-like pathogenesis and gene expression in Down syndrome iPSC-derived neurons. Stem Cell Reports *11*, 32–42.

Pasca, A.M., Sloan, S.A., Clarke, L.E., Tian, Y., Makinson, C.D., Huber, N., Kim, C.H., Park, J.Y., O'Rourke, N.A., Nguyen, K.D., et al. (2015). Functional cortical neurons and astrocytes from human pluripotent stem cells in 3D culture. Nat. Methods *12*, 671–678.

Richetin, K., Leclerc, C., Toni, N., Gallopin, T., Pech, S., Roybon, L., and Rampon, C. (2015). Genetic manipulation of adult-born hippocampal neurons rescues memory in a mouse model of Alzheimer's disease. Brain *138*, 440–455.

Richetin, K., Moulis, M., Millet, A., Arrazola, M.S., Andraini, T., Hua, J., Davezac, N., Roybon, L., Belenguer, P., Miquel, M.C., et al. (2017a). Amplifying mitochondrial function rescues adult neurogenesis in a mouse model of Alzheimer's disease. Neurobiol. Dis. *102*, 113–124.

Richetin, K., Petsophonsakul, P., Roybon, L., Guiard, B.P., and Rampon, C. (2017b). Differential alteration of hippocampal function and plasticity in females and males of the APPxPS1 mouse model of Alzheimer's disease. Neurobiol. Aging *57*, 220–231.

Roybon, L., Deierborg, T., Brundin, P., and Li, J.Y. (2009a). Involvement of Ngn2, Tbr and NeuroD proteins during postnatal olfactory bulb neurogenesis. Eur. J. Neurosci. *29*, 232–243.

Roybon, L., Hjalt, T., Stott, S., Guillemot, F., Li, J.Y., and Brundin, P. (2009b). Neurogenin2 directs granule neuroblast production and amplification while NeuroD1 specifies neuronal fate during hippocampal neurogenesis. PLoS One *4*, e4779.

Roybon, L., Lamas, N.J., Garcia, A.D., Yang, E.J., Sattler, R., Lewis, V.J., Kim, Y.A., Kachel, C.A., Rothstein, J.D., Przedborski, S., et al. (2013). Human stem cell-derived spinal cord astrocytes with defined mature or reactive phenotypes. Cell Rep. *4*, 1035–1048.

Roybon, L., Mastracci, T.L., Li, J., Stott, S.R., Leiter, A.B., Sussel, L., Brundin, P., and Li, J.Y. (2015). The origin, development and molecular diversity of rodent olfactory bulb glutamatergic neurons distinguished by expression of transcription factor NeuroD1. PLoS One *10*, e0128035.

Sakaguchi, H., Kadoshima, T., Soen, M., Narii, N., Ishida, Y., Ohgushi, M., Takahashi, J., Eiraku, M., and Sasai, Y. (2015). Generation of functional hippocampal neurons from self-organizing human embryonic stem cell-derived dorsomedial telencephalic tissue. Nat. Commun. *6*, 8896.

Sarkar, A., Mei, A., Paquola, A.C.M., Stern, S., Bardy, C., Klug, J.R., Kim, S., Neshat, N., Kim, H.J., Ku, M., et al. (2018). Efficient generation of CA3 neurons from human pluripotent stem cells enables modeling of hippocampal connectivity in vitro. Cell Stem Cell *22*, 684–697.e9.

Saura, C.A., Parra-Damas, A., and Enriquez-Barreto, L. (2015). Gene expression parallels synaptic excitability and plasticity changes in Alzheimer's disease. Front. Cell Neurosci *9*, 318.

Scheff, S.W., Neltner, J.H., and Nelson, P.T. (2014). Is synaptic loss a unique hallmark of Alzheimer's disease? Biochem. Pharmacol. *88*, 517–528.

Schuff, N., Woerner, N., Boreta, L., Kornfield, T., Shaw, L.M., Trojanowski, J.Q., Thompson, P.M., Jack, C.R., Jr., Weiner, M.W., and Alzheimer's Disease Neuroimaging, I. (2009). MRI of hippocampal volume loss in early Alzheimer's disease in relation to ApoE genotype and biomarkers. Brain *132*, 1067–1077.

Shioya, M., Obayashi, S., Tabunoki, H., Arima, K., Saito, Y., Ishida, T., and Satoh, J. (2010). Aberrant microRNA expression in the brains of neurodegenerative diseases: miR-29a decreased in Alzheimer disease brains targets neurone navigator 3. Neuropathol. Appl. Neurobiol. *36*, 320–330.

Siebzehnrubl, F.A., Raber, K.A., Urbach, Y.K., Schulze-Krebs, A., Canneva, F., Moceri, S., Habermeyer, J., Achoui, D., Gupta, B.,

Steindler, D.A., et al. (2018). Early postnatal behavioral, cellular, and molecular changes in models of Huntington disease are reversible by HDAC inhibition. Proc. Natl. Acad. Sci. U S A. *115*, E8765–E8774.

Sloan, S.A., Darmanis, S., Huber, N., Khan, T.A., Birey, F., Caneda, C., Reimer, R., Quake, S.R., Barres, B.A., and Pasca, S.P. (2017). Human astrocyte maturation captured in 3D cerebral cortical spheroids derived from pluripotent stem cells. Neuron *95*, 779–790.e6.

Sorbi, S., Nacmias, B., Forleo, P., Piacentini, S., Amaducci, L., and Provinciali, L. (1993). APP717 and Alzheimer's disease in Italy. Nat. Genet. *4*, 10.

Tampellini, D., Capetillo-Zarate, E., Dumont, M., Huang, Z., Yu, F., Lin, M.T., and Gouras, G.K. (2010). Effects of synaptic modulation on beta-amyloid, synaptophysin, and memory performance in Alzheimer's disease transgenic mice. J. Neurosci. *30*, 14299–14304.

Venegas, C., Kumar, S., Franklin, B.S., Dierkes, T., Brinkschulte, R., Tejera, D., Vieira-Saecker, A., Schwartz, S., Santarelli, F., Kummer, M.P., et al. (2017). Microglia-derived ASC specks cross-seed amyloid-beta in Alzheimer's disease. Nature *552*, 355–361.

Watanabe, K., Kamiya, D., Nishiyama, A., Katayama, T., Nozaki, S., Kawasaki, H., Watanabe, Y., Mizuseki, K., and Sasai, Y. (2005). Directed differentiation of telencephalic precursors from embryonic stem cells. Nat. Neurosci. *8*, 288–296.

Woodard, C.M., Campos, B.A., Kuo, S.H., Nirenberg, M.J., Nestor, M.W., Zimmer, M., Mosharov, E.V., Sulzer, D., Zhou, H., Paull, D., et al. (2014). iPSC-derived dopamine neurons reveal differences between monozygotic twins discordant for Parkinson's disease. Cell Rep. *9*, 1173–1182.

Woodruff, G., Reyna, S.M., Dunlap, M., Van Der Kant, R., Callender, J.A., Young, J.E., Roberts, E.A., and Goldstein, L.S. (2016). Defective transcytosis of APP and lipoproteins in human iPSC-derived neurons with familial Alzheimer's disease mutations. Cell Rep *17*, 759–773.

Wyss-Coray, T. (2016). Ageing, neurodegeneration and brain rejuvenation. Nature *539*, 180–186.

Yamakawa, H., Cheng, J., Penney, J., Gao, F., Rueda, R., Wang, J., Yamakawa, S., Kritskiy, O., Gjoneska, E., and Tsai, L.H. (2017). The transcription factor Sp3 cooperates with HDAC2 to regulate synaptic function and plasticity in neurons. Cell Rep. *20*, 1319–1334.

Yu, D.X., Di Giorgio, F.P., Yao, J., Marchetto, M.C., Brennand, K., Wright, R., Mei, A., McHenry, L., Lisuk, D., Grasmick, J.M., et al. (2014). Modeling hippocampal neurogenesis using human pluripotent stem cells. Stem Cell Reports *2*, 295–310.

Zhao, T., Fu, Y., Zhu, J., Liu, Y., Zhang, Q., Yi, Z., Chen, S., Jiao, Z., Xu, X., Xu, J., et al. (2018). Single-cell RNA-seq reveals dynamic early embryonic-like programs during chemical reprogramming. Cell Stem Cell *23*, 31–45 e37.

Stem Cell Reports, Volume *15*

Supplemental Information

Human iPSC-Derived Hippocampal Spheroids: An Innovative Tool for Stratifying Alzheimer Disease Patient-Specific Cellular Phenotypes and Developing Therapies

Yuriy Pomeshchik, Oxana Klementieva, Jeovanis Gil, Isak Martinsson, Marita Grønning Hansen, Tessa de Vries, Anna Sancho-Balsells, Kaspar Russ, Ekaterina Savchenko, Anna Collin, Ana Rita Vaz, Silvia Bagnoli, Benedetta Nacmias, Claire Rampon, Sandro Sorbi, Dora Brites, György Marko-Varga, Zaal Kokaia, Melinda Rezeli, Gunnar K. Gouras, and Laurent Roybon

SUPPLEMENTAL INFORMATION DIRECTORY

Human iPSC-derived hippocampal spheroids: an innovative tool for modeling typical and atypical familial Alzheimer's disease and developing therapies

Yuriy Pomeshchik, Oxana Klementieva, Jeovanis Gil, Isak Martinsson, Marita Grønning Hansen, Tessa de Vries, Anna Sancho Balsells, Kaspar Russ, Ekaterina Savchenko, Anna Collin, Ana Rita Vaz, Silvia Bagnoli, Benedetta Nacmias, Claire Rampon, Sandro Sorbi, Dora Brites, György Marko-Varga, Zaal Kokaia, Melinda Rezeli, Gunnar K. Gouras, Laurent Roybon

Pomeshchik et al. Supplementary Fig.1: **Generation and characterization of iPSCs from AD patients and healthy individuals** - related to Fig. 1
a) Summary of the iPSC lines used in this study.
b) Bright-field images showing representative colonies of APP variant, PS1 variant and gender-matched control iPSCs. Scale bars: 100 µm.
c) Immunostaining for Sendai virus, pluripotent markers OCT4, NANOG, TRA1-81, markers of the three germ layers alpha-fetoprotein (AFP) (endoderm), smooth muscle antibody (SMA) (mesoderm), and beta III-tubulin (B-III-TUB) (ectoderm) in APP variant, PS1 variant and gender-matched control iPSC colonies. Nuclei are counterstained with 4',6-diamidino-2-2phenylindole (DAPI). iPSC colonies stain positive for alkaline phosphatase activity and form EBs. Scale bars: 100 µm.
d) Karyogram of APP variant, PS1 variant and gender-matched control iPSC lines stained using Giemsa (G-banding).
e) Sequencing confirming the heterozygeous p.R278K PS1 and homozygous p.V717I APP variation in patient-derived iPSCs.
f) STR allele analysis for PS1 and APP variant iPSC lines showing identity to parental fibroblasts.

Pomeshchik et al. Supplementary Fig. 2: **Bright-field images of iPSC cultures and GABA expression in neuronal cultures aged 56 DIV** – related to Fig. 1
a) Bright-field images showing the morphology of APP variant, PS1 variant and gender-matched control cultures aged 56 DIV. Scale bars: 100 µm.
b) Percentage of GABA-positive neurons in APP variant, PS1 variant and gender-matched control cultures aged 56 DIV. Results are presented as mean ± S.E.M. n = 1-3 independent differentiations per clone for N = 3 iPSC clones per genotype. Statistical analysis by two-tailed t-test.

Pomeshchik et al. Supplementary Fig. 3: **Accumulation and secretion of amyloid-β (Aβ) peptides in HSs** – related to Fig. 2
a) Characterization of Aβ accumulation (intracellular Aβ) in APP variant, PS1 variant and gender-matched control HSs at DIV 100. Aβ38, Aβ40 and Aβ42 in HS lysates were measured at day 4 after the last medium change. For quantitation, data were normalized to the total protein. Results are presented as mean ± S.E.M. n = 3 independent differentiations per genotype. Statistical analysis by two-tailed t-test.
b) Characterization of Aβ secretion (extracellular Aβ) in APP variant, PS1 variant and gender-matched control HSs at DIV 100. Aβ38, Aβ40 and Aβ42 secreted from HSs into the medium were measured at day 4 after the last medium change. For quantitation, data were normalized to the total protein. Results are presented as mean ± S.E.M. n = 3 independent differentiations

per genotype. P values: * = P<0.05; ** = P<0.01; *** = P<0.001. Statistical analysis by two-tailed t-test.

Pomeshchik et al. Supplementary Fig. 4: **APP variant HSs exhibit increased protein aggregation.** – related to Fig. 4
Bar diagrams reflecting the β-sheet structure content, as shown by the absorbance ratios 1628 cm^{-1} to 1656 cm^{-1} for APP variant, PS1 variant and gender-matched control hippocampal neurons at DIV56. Results are presented as mean ± S.E.M. n = 2-3 iPSC clones per genotype. P values: * = P<0.05. Statistical analysis by two-tailed t-test.

Pomeshchik et al. Supplementary table 1. **Electrophysiological characteristics of human iPSC-derived hippocampal neurons**

Pomeshchik et al. Supplementary table 2. **Summary of the quantitative proteomic data**

Pomeshchik et al. Supplementary table 3. **Summary of the quantitative proteomic data – a subset of synaptic related proteins**

Pomeshchik et al. Supplementary table 4. **Relative expression of genes in control and APP variant hippocampal neurons after GFP or ND1 gene transfer.**

Pomeshchik et al. Supplementary table 5. **Relative expression of annotated genes dysregulated in control and APP variant hippocampal neurons upon ND1 gene delivery.**

Pomeshchik et al. Supplementary table 6. **Primary and secondary antibodies used for immunocyto- and immunohistochemistry**

Pomeshchik et al. Supplementary table 7. **List of primer sequences used in qRT-PCR for microRNA expression**

Pomeshchik et al. Supplementary table 8. **Primary and secondary antibodies used for Western Blotting**

SUPPLEMENTAL EXPERIMENTAL PROCEDURES

All materials were purchased from ThermoFisher Scientific, unless specified.

Generation of iPSCs
Primary human dermal fibroblasts were harvested by punch skin biopsy from AD patients and healthy donors after written informed consent. Fibroblasts harboring PS1 mutation were obtained from the Galliera Genetic Bank; the fibroblasts harboring APP mutation were provided by Prof. Sorbi. Fibroblasts were cultured and expanded in Dulbecco's modified eagle's medium (DMEM) supplemented with 10% fetal bovine serum and 1% Penicillin-Streptomycin (P/S; v/v). Cells were passaged with 0.05% trypsin, cryopreserved in liquid nitrogen and stored at -150 °C. For reprogramming, fibroblasts were seeded on 0.1% gelatin-coated 12-well plate (75.000 cells/well) and two days later transduced using the three vector preparations (MOI=5, 5, 3) included in the CytoTune™-iPS 2.0 Sendai Reprogramming Kit. Reprogramming factors (OCT-3/4, KLF-4, SOX-2 and c-MYC) were delivered by using non-integrating Sendai virus vectors from the kit. The medium was replaced daily for 7 days, after which the cells were re-

seeded onto irradiated mouse embryonic fibroblast feeder cells (CF-1 MEF, GlobalStem). From the day 8 and until day 28, the cells were cultured in WiCell medium composed of advanced DMEM/F12, 20% Knock-Out Serum Replacement (v/v), 1% nonessential amino acids (NEAA; v/v), 2 mM L-glutamine, and 0.1mM β-mercaptoethanol (Sigma-Aldrich) supplemented with 20 ng/ml FGF2. At day 28, several colonies were collected and expanded as single clones for 7 days. Two to three iPSC clones per individuals were selected for further expansion and characterization (Supplementary Fig. 1a). The reprogramming of patient cells into iPSC was approved by the Swedish work environment authority. All individuals carried the common neutral isoform ApoE3/E3 of the *APOE* gene. An additional female control line (TALSCTRL15.12) was received from Target ALS (http://www.targetals.org/) and NHCDR Repositories hosted by RUCDR.

Alkaline phosphatase activity
Alkaline phosphatase activity was detected using Alkaline Phosphatase Staining Kit (Stemgent, MA) according to the manufacturer's protocol.

Karyotype analysis
The G-banding analysis was performed at 300–400 band resolution in a clinical diagnostic setting.

Variation sequencing
Genomic DNA was extracted from fibroblasts and iPSCs with the use of conventional lysis buffer composed of 100 mM Tris (pH 8.0), 200 mM NaCl, 5 mM EDTA, 1.5 mg/ml Proteinase K, and 0.2% SDS in distilled autoclaved water. The presence of APP and PS1 variations was confirmed by direct DNA sequencing (Macrogen Europe, Amsterdam, The Netherlands).

DNA fingerprinting
DNA fingerprinting analysis was performed by the IdentiCell STR profiling service (Department of Molecular Medicine, Aarhus University Hospital, Skejby, Denmark). In vitro differentiation by embryoid body (EB) formation iPSCs were transferred onto ultra-low adherent 24-well plates (Corning) and for 14 days were grown as free-floating embryoid bodies (EBs) in WiCell medium supplemented with 20ng/ml FGF2. For subsequent spontaneous differentiation, EBs were seeded on a 0.1% gelatin-coated 96-well plate in DMEM media containing 10% fetal bovine serum (v/v) and 1% Penicillin-Streptomycin (v/v). Two weeks later, the cells were fixed and immunostained for germ layer markers.

Differentiation of iPSCs to hippocampal spheroids
For differentiation to HSs, one to three clones per individuals were thawed and expanded over several passages. To form EBs, intact iPSC colonies were dissociated using dispase (1 mg/ml) for ~20 minutes and transferred into ultra-low adherent flasks (Corning) in WiCell medium supplemented with 20 ng/ml FGF2 and 20µM ROCK-Inhibitor Y-27632 (Selleck Chemicals, Munich, Germany). Next day, WiCell was replaced with neural induction medium (NIM) composed of advanced DMEM/F12, 2% B27 Supplement without vitamin A (v/v), 1% N2 Supplement (v/v), 1% NEAA (v/v), 2 mM L-glutamine and 1% Penicillin-Streptomycin (v/v). For dorsomedial telencephalic neural specification, LDN-193189 (Stemgent, 0.1 µM), Cyclopamine (Selleck Chemicals, 1 µM), SB431542 (Sigma-Aldrich, 10 µM) and XAV-939 (Tocris, 5 µM) were added to the medium for the first ten days with medium change every other day. On the tenth day, the free-floating spheres were transferred to neuronal differentiation medium (NDM) containing Neurobasal® medium, 1% N2 (v/v), 1% NEAA (v/v), L-glutamine and 1% Penicillin-Streptomycin (v/v). To promote hippocampal differentiation, NDM was

supplemented with CHIR-99021 (Stemgent, 0.5 mM) and brain derived neurotrophic factor (BDNF, PeproTech, 20 ng/ml) for 90 days with medium changes every second day. On the day in vitro 50, spheroids were dissociated into single cells with Trypsin 1X and plated in NDM on polyornithine/laminin coated surfaces. Adherent cells were grown for 6 days in NDM supplemented with CHIR-99021 (0.5 mM) and BDNF (20 ng/ml) with media change every second day.

Differentiation of iPSCs to astrocytes
On the day in vitro 50, spheroids were dissociated and transferred into new ultra-low adherent flask in neural expansion medium (NEM) containing DMEM/F12, 2% B27 without vitamin A (v/v), 1% NEAA (v/v), 2 mM L-glutamine, 1% Penicillin-Streptomycin (v/v), and 0.2 µg/ml heparin (Sigma-Aldrich). NEM was supplemented with 20 ng/ml FGF2 and 20ng/ml EGF (Peprotech) for 30 days with media change every 2-3 days. On the day in vitro 80, free-floating spheroids were dissociated into single cells and seeded to polyornithine/laminin coated culture flasks in NDM. To promote astroglial differentiation, NDM was supplemented with ciliary neurotrophic factor (CNTF, R&D Systems, 20 ng/ml) for 40 days with media change every 2-3 days. Cells were passaged at confluency.

In vivo transplantation
For in vivo transplantation, male 10–12 weeks old RAG-1-deficient mice (n=3) generated by Lund University breeding facility were used. All experimental procedures were conducted in accordance with the European Union Directive (2010/63/EU) on the subject of animal rights, and were approved by the committees for the use of laboratory animals at Lund University and the Swedish Board of Agriculture. On the day in vitro 50, spheroids were dissociated into single cells and resuspended in PBS to a final concentration of 100 000 cells/µl. The mice were anesthetized with isoflurane (Baxter, Deerfield, IL) in oxygen (initial dose of 5% which was reduced to 1–1.5% for maintenance of surgical depth anesthesia) delivered through a nose mask during the surgery. The body temperature was maintained at 37.0°C during the surgery using a thermostatically controlled rectal probe connected to a homeothermic blanket. The skull was exposed by the skin incision after subcutaneous analgesia with Marcaine (50 µl of 2.5 mg/ml stock solution, Astra Zeneca). Transplantation was stereotaxically performed for each mouse through drilled hole in the skull using a 5-µl Hamilton syringe with 32-gauge needle and an injecting minipump ((Nanomite Injector
Syringe Pump; Harvard Apparatus, Holliston, MA, USA). A volume of 2 µl of cell suspension was injected at a rate of 0.5 µl/min at the following coordinates (from bregma and brain surface): anterior/posterior (AP): -2.54 mm; medial/lateral (M/L): -1.5 mm; dorsal/ventral (D/V): -1-75 mm. The needle was left in situ for 7 min after injection before being slowly raised, and the wound was sutured.

Rodent brain tissue collection and processing
Five weeks after transplantation, the mice were transcardially perfused with PBS followed by perfusion with 50 ml of 4 % paraformaldehyde (PFA). The dissected brains were post-fixed in the same fixative overnight at 4°C. After fixation, brains were cryoprotected in 30% sucrose (Sigma- Aldrich) for 48 hours at 4°C before being cut into 40 µm thick coronal serial sections on a sliding microtome (Leica Microsystems GmH, Wetzlar Germany). The free-floating brain sections were then stored in antifreeze solution at -20°C until immunohistochemical staining.

Immunostainings, microscopy and image analyses
For immunocytochemistry, the adherent cells aged 56 days were fixed with 2% PFA for 10 min followed by fixation with 4% PFA for 10 min at RT, blocked for 1hr at RT with 10% normal

donkey (NDS, VWR) or normal goat serum (NGS) in PBS with 0.1% Triton-X (Sigma-Aldrich) and incubated overnight with target primary antibodies (Supplementary Table 6) prepared in 10% NDS/NGS in PBS at 4ºC. On the next day, the cells were incubated with appropriate Alexa-fluor 488, 555- or 647- conjugated secondary antibodies (Supplementary Table 6) in PBS for 1hr at RT in the dark. Cell nuclei were counterstained with DAPI (1:10,000). Spheroids aged 100 days were fixed for 45 minutes with 4% PFA at 4ºC followed by incubation in PBS overnight. Next day, spheroids were cryoprotected with 30% sucrose for 4-6 hours, embedded in Tissue-Tek O.C.T. Compound (Sakura Finetek, Zouterwoude, the Netherlands), snap frozen on liquid nitrogen and cryostat (Leica Microsystems GmH, Wetzlar Germany) at 30 µm and then stored at -80°C until immunohistochemical staining. For immunocytochemistry, frozen sections were air-dried for 1 hour, washed 3 times in PBS with 0.5% Tween (PBST) pH 7.4 and permeabilized with 0.3% Triton X-100 (for spheroid sections) or 0.4% Triton X-100 (for mouse brain sections) for 30 min followed by blocking with 10% NDS in PBST and incubation with target primary antibodies (Supplementary Table 6) in 10% NDS in PBST at RT with slow agitation overnight. For staining detecting human nuclei (HuNu), M.O.M.TM Mouse Ig Blocking Reagent (Vector Laboratories) was used to block endogenous mouse antibody in the brain sections. On the next day, sections were incubated with appropriate Alexa-fluor 488-, 555- or 647-conjugated secondary antibody (Supplementary Table 6) in 10% NDS in PBST at RT for 1 hour. Image acquisition was performed using inverted epifluorescence microscope LRI-Olympus IX- 73 or confocal microscope (Carl Zeiss, Germany). Automated quantitative image analysis of stained cell cultures was performed with the MetaMorph Software V7.6 (Molecular Devices) using the Multi-Wavelength Cell Scoring application. For a specific marker, positive cells were identified as having signal intensity above the selected intensity threshold. Intensity thresholds were set blinded to sample identity. Three-dimensional surface reconstructions of confocal z-stacks were created using the surface object and filament functions in Imaris software 8.4.1 (Bitplane).

Morphometric analysis

All morphometric analyses were performed using ImageJ software on the adherent cells aged 56 days stained with the MAP2 antibody. For soma area calculation, the Map2-positive cell bodies were manually outlined and the area within was measured. The lengths of all individual dendrites were counted and summed to estimate total dendrite length. To assess arborization, dendrites were subjected to Sholl analysis using the Simple Neurite Tracer plugin (Ferreira et al., 2014). Briefly, concentric circles were drawn around the cell center with increasing radii and the algorithm then counts the number of intersections of the binarized neurite skeletons with the concentric circles.

Electrophysiology

Electrophysiology recordings were performed on the adherent cells aged 56-58 days. Coverslips were placed in the recording chamber during recordings and constantly perfused with carbogenated artificial cerebral spinal fluid (ACSF, in mM: 119 NaCl, 2.5 KCl, 1.3 $MgSO_4$, 2.5 $CaCl_2$, 26 $NaHCO_3$, 1.25 NaH_2PO_4, and 11 glucose, pH ~7.4) at 34°C. Whole-cell patch-clamp recordings were performed using a potassium gluconate-based intracellular solution (in mM: 122.5 potassium gluconate, 12.5 KCl, 10 HEPES, 2.0 Na_2ATP, 0.3 Na_2-GTP, and 8.0 NaCl). Biocytin (1-3mg/ml, Biotium) was dissolved in the pipette solution for post-hoc identification of recorded cells. Whole-cell patch-clamp recordings were performed with a HEKA double patch clamp EPC10 amplifier using PatchMaster for data acquisition. Voltage and current clamp recordings were used for the electrophysiological characterization. Sodium and potassium currents were evoked by a series of 200ms long voltage steps ranging from -70mV to +40mV in 10mV steps and their sensitivity to, respectively, 1µM TTx (Latoxan) and

10mM TEA (Abcam) were determined. A series of current steps, going from 0pA to 190pA in 10pA steps and lasting 500ms, were performed from a membrane potential of approximately -70mV to determine the cells ability to generate APs. To study the AP characteristics a current ramp protocol going from 0-300pA in 1s was used. Current was injected, when needed, to keep the membrane potential around -70mV. Data were analyzed offline with FitMaster (HEKA Elektronik, Lambrecht, Germany) and IgorPro (Wavemet— rics, Lake Oswego, OR, USA).

Quantitative RT-PCR
Expression of miRNAs was assessed by qRT-PCR in the adherent cells aged 56 days. Total RNA was extracted from cells using TRIzol® (LifeTechnologies, Carlsbad, CA, USA), according to manufacturer's instructions and as previously described (Gomes et al., 2019). Total RNA was quantified using Nanodrop ND-100 Spectrophotometer (NanoDrop Technologies, Wilmington, DE, USA) and conversion of cDNA was achieved with the miRCURY LNA RT Kit (Qiagen, Hilden, Germany), using 5 ng total RNA. The Power SYBR® Green PCR Master Mix (Applied Biosystems) were used in combination with predesigned primers (Qiagen), summarized in Supplementary Table 7, using as reference genes U6, RNU1A1 and a RNA-spike provided with the kit (all from Qiagen). The reaction conditions consisted of polymerase activation/denaturation and well-factor determination at 95ºC for 10 min, followed by 50 amplification cycles at 95ºC for 10 s and 60ºC for 1 min (ramprate 1.6º/s). Each sample was performed in triplicate, and a non-template control was included for miRNA analysis. The expression fold change vs. respective controls was determined by the 2- ΔΔCT equation.

MSD MULTI-ARRAY Assay
Culture media (CM) were collected from HSs aged 100 days at four days after the last medium change. The pellets of HSs were lysed with M-PER Mammalian Protein Extraction Reagent complemented with Halt Protease Inhibitor Cocktail (100X). Total protein was determined using the PierceTM BCA Protein Assay Kit according to the manufacturer instructions. Aβ levels were quantified in CM and HS lysates using the multiplex Aβ Peptide Panel 1 (6E10, MesoScale Discovery, USA) according to the manufacturer's protocol. Samples below or above the sensitivity of the assay were set to zero or the max value, respectively. Concentrations were normalized to total protein (pg/µg protein) and averaged between three wells per differentiation.

Western blot analysis
Spheroids aged 100 days were lysed with RIPA buffer or M-PER Mammalian Protein Extraction Reagent complemented with Halt Protease Inhibitor and Halt Phosphatase Inhibitor Cocktails. Protein concentrations were determined using the PierceTM BCA Protein Assay Kit according to the manufacturer instructions. Samples were mixed with loading buffer, heated at 70 °C for 10 min and loaded into 10–20% Tricine gels. After electrophoresis the samples were transferred to polyvinylidine difluoride (PVDF) membranes. Membranes were blocked in PBS containing 0.1% Tween-20 (PBST) and 5% milk or 5 % BSA, and incubated with target primary antibodies (Supplementary Table 8) overnight. Next day, membranes were incubated with HRP-conjugated secondary antibodies (Supplementary Table 8) for 1 h diluted in PBST and 5% milk or 5% BSA. The blots were then visualized using Pierce Enhanced Chemo-Luminescence solution and imaged in a Biorad Chemi-Doc chemo-luminescence system (BioRad). Bands were quantified using Image Lab (Bio-Rad Laboratories).

Fourier transform infrared micro-spectroscopy
FTIR is a spectroscopic tool that can be used to detect molecular vibrations of main-chain carbonyl groups, that occur in the wave number range of 1500 to 1700 cm^{-1} (Amide I and II

regions), allowing the detection of β-sheet structures (Baker et al., 2014; Miller et al., 2013). FTIR further allows to determine protein aggregation measured as average of β-sheet structures (1628 cm^{-1}) to total protein (1656 cm^{-1}) absorption. FTIR micro-spectroscopy analyses were performed on the parental fibroblasts, neurons aged 56 days, astrocytes aged 120 days, and HSs aged 100 days, and using two different platforms. Cell pellets were homogenized, spread (1 µl) on the 1 mm thick CaF_2 spectrophotometric window and dried under nitrogen flow. For reproducibility, infrared spectra were taken from different areas of the cell pellet deposited on CaF_2. The HSs spectra were recorded on a Hyperion 3000 IR microscope (BrukerScientific Instruments, Billerica, MA, USA) coupled to a Tensor 27, which was used as the IR light source with 15Å~ IR objective and MCT (mercury cadmium telluride) detector, located at Lund University (Sweden). The measuring range was 900–4000 cm^{-1}, the spectra collection was done in transmission mode at 4 cm^{-1} resolution from 250-500 co-added scans. For the parental fibroblasts, neurons and astrocytes FTIR spectro-microscopy was performed at the SMIS beamline of the SOLEIL synchrotron (France) using a Thermo Fisher Scientific Continuum XL FTIR microscope through a 32× magnification, 0.65 NA Schwarzschild objective. For the collection parameters were: spectral range 1000–4000 cm^{-1}, in transmission mode at 4 cm^{-1} spectral resolution, with 8 µm × 8 µm aperture dimensions, using 256 coadded scans. Background spectra were collected from a clean area of the same CaF_2 window. All measurements were made at room temperature. For analysis of FTIR spectra OPUS software (Bruker) and Orange (University of Ljubljana) were used and included atmospheric compensation. Derivation of the spectra to the second order using Savitsky-Golay of 3rd polynomial order 3 with 9 smoothing points, was used to unmask the number of discriminative features and to eliminate the baseline contribution.

Viral vectors, gene transfer and FACS-based purification of GFP-positive cells
Spheroids aged 30 DIV were dissociated into single cells and plated in NDM on poly-ornithine/laminin coated surfaces. Two hours later the cells were transduced with either the eGFP or ND1-expressing Moloney murine leukemia-derived retroviral vectors (pCMMP-IRES2eGFP-WPRE or pCMMP-NeuroD1-IRES2eGFP-WPRE) at a multiplicity of infection (MOI) of 5, in proliferation medium supplemented by protamine-sulfate (4 mg/ ml, Sigma-Aldrich). The viruses were produced and titrated as previously described (Roybon et al., 2009). Adherent cells were then cultured for 20 days in NDM supplemented with CHIR-99021 and BDNF. Media was changed every other day. Twenty days later, the cells were detached with Trypsin 1X, resuspended in Neurobasal medium without red-phenol and transferred to a FACS tube. After centrifugation, the pellet was resuspended in Neurobasal medium without red-phenol containing 10% normal serum. For exclusion of dead cells, fluorescent live-cell impermeant DNA dye (DRAQ7, BioStatus, 1:1000) was added, and the cells were incubated on ice for 5 min before sorting. Samples were analyzed using a BD FACSAria III (BD Biosciences) with FACSDiva v8.0 software (BD Biosciences) at the MultiPark Cellomics and Flow Cytometry Core technical platform, at Lund University. The GFP-positive cells were collected in Eppendorf tubes, snap-frozen in liquid nitrogen and stored at -80°C until RNA extraction.

Affymetrix GeneChip microarray
Total RNA was extracted from the FACS-sorted GFP+ transduced cells using RNeasy Micro Kit (QIAGEN, Hilden, Germany), according to the manufacturer instructions. After adding 350µl buffer RLT containing 1% beta-mercaptoethanol, the thawed samples were mixed using a vortex and homogenized with QIAshredder spin columns. Next, 1 volume of 70% ethanol was added and the samples were applied to RNeasy MinElute spin columns followed by an on-column DNase digestion and several wash steps. Finally, total RNA was eluted in 14 µl of

nuclease free water. Purity and integrity of the RNA was assessed on the Agilent 2100 Bioanalyzer with the RNA 6000 Nano LabChip reagent set (Agilent, Palo Alto, CA, USA). Sample preparation for microarray hybridization was carried out as described in the Affymetrix GeneChip WT PLUS Reagent Kit User Manual (Affymetrix, Inc., Santa Clara, CA, USA). In brief, 200 ng of total RNA was used to generate double-stranded cDNA. 15 µg of subsequently synthesized cRNA was purified and reverse transcribed into sense-strand (ss) cDNA, whereat unnatural dUTP residues were incorporated. Purified ss cDNA was fragmented using a combination of uracil DNA glycosylase (UDG) and apurinic/apyrimidinic endonuclease 1 (APE 1) followed by a terminal labeling with biotin. 5.5 µg fragmented and labeled ss cDNA were hybridized to Affymetrix Clariom D human arrays for 16 h at 45° C and 60 rpm in a GeneChip hybridization oven 640. Hybridized arrays were washed and stained in an Affymetrix Fluidics Station FS450, and the fluorescent signals were measured with an Affymetrix GeneChip Scanner 3000 7G. Fluidics and scan functions were controlled by the Affymetrix GeneChip Command Console v4.3.3 software. RNA extraction and sample processing were performed at an Affymetrix Service Provider and Core Facility, "KFB - Center of Excellence for Fluorescent Bioanalytics" (Regensburg, Germany; www.kfbregensburg.de).
Summarized probe set signals in log2 scale were calculated by using the GCCN-SST-RMA algorithm with the Affymetrix GeneChip Expression Console v1.4 Software. The output matrix was processed in Perseus framework. To simplify the analysis, we examined transcriptomic changes for annotated genes having a gene name and a protein accession number (representing a total of 21,467 genes out of the 135,750 probes examined). The 2D annotation enrichment analysis was performed using the biological pathways information available in KEGG repository and the procedure was provided in Perseus software. Briefly, the average intensity was calculated for each transcript in the four groups (Control:GFP, Control:ND1, APP:GFP, and APP:ND1). Later, the GFP values were subtracted from their corresponding ND1 values in both control and APP variant. The resulting values correspond to the difference between ND1 and GFP in both control and APP variant. For the enrichment analysis, pathways significantly enriched were filtered through a FDR (Benjamini-Hochberg) with a q-value threshold of 0.01.

Label-free liquid chromatography-tandem mass spectrometry
100 µl of lysis buffer (50 mM dithiothreitol (DTT) and 2 w/v% sodium dodecyl sulfate (SDS) in 100 mM Tris-Cl buffer, pH 8.6) was added to the spheroid sample. The sample was boiled at 95 °C for 5 minutes, then proteins were extracted by sonication executing 30 cycles of 15s on and 15 s off at 4 °C using a Bioruptor plus (model UCD-300) (Diagenode). Iodoacetamide (IAA) was added to the lysate at the final concentration of 20 mM and was incubated at RT for 30 minutes in dark. The proteins were precipitated using 9 volumes of cold ethanol O/N at -20 °C and the protein pellet was dissolved in 50 mM ammonium-bicarbonate (AmBic) with 0.5% SDC (sodium deoxycholate). The protein concentration was measured using the Micro BCA Protein Assay Kit (Thermo Scientific) prior the digestion. Sequencing Grade Modified Trypsin (Promega, Madison, WI) was added to the sample in 1:25 enzyme-substrate ratio and was incubated for 18 hours at 37 °C. SDC was removed by EtOAc (ethyl acetate) extraction under acidic conditions (Gil et al., 2017). Peptide concentration of the sample was measured using the Pierce Quantitative Colorimetric Peptide Assay. The peptide samples were analyzed on a Q Exactive HF-X mass spectrometer coupled to an Ultimate 3000 RSCLnano pump (Thermo Scientific). Acclaim PepMap100 C18 (5 µm, 100 Å, 75 µm i.d. x 2 cm, nanoViper) was used as trap column and EASY-spray RSLC C18 (2 µm, 100 Å, 75 µm i.d. x 25 cm) as analytical column. Solvent A was 0.1% formic acid (FA), solvent B was 80% acetonitrile (ACN) with 0.08% FA. The flow-rate was set to 0.3 µl/min and the column temperature was 45 °C. The peptides were separated using a 120 min non-linear gradient and analyzed with a top 20 DDA (data dependent acquisition) method. MS1 scans were acquired using a mass range of 375-1500

m/z, with a resolution of 120,000 (@ 200 m/z), a target AGC value of 3e06 and a maximum injection time (IT) of 100 ms. The 20 most intense peaks were fragmented with a normalized collision energy (NCE) of 28. MS2 scans were acquired with a resolution of 15,000, a target AGC value of 1e05 and a maximum IT of 50 ms. The ion selection threshold was set to 8.00e03 and the dynamic exclusion to 40 s while single charged ions were excluded from the analysis. The precursor isolation window was set to 1.2 Th. The raw files were analyzed using Proteome Discoverer (PD) v2.2 (Thermo Scientific). Peptide and protein identification were performed against UniProt human database (released 20180207, 42213 sequences including isoforms) with SEQUEST HT as search engine. The following parameters were set during the search: max. 2 missed cleavages, cysteine carbamidomethylation as static modification, methionine oxidation, phosphorylation at serine, threonine and tyrosine as dynamic modifications, acetylation as a dynamic N-terminal protein modification, 10 ppm precursor mass tolerance and 0.02 Da fragment mass tolerance. 1% false discovery rate (FDR) at both peptide and protein levels was applied. For label-free quantitative analysis, protein abundance data was processed with the aid of Perseus v1.6.1.2. In order to normalize and standardize the data, raw intensities were log2 transformed and subtracted from the median of all proteins in each sample. To determine proteins with significant differences in abundance between samples under study, t-tests followed by FDR adjustment were performed. In all cases the q-value was set at 5%. The quantitative data was also used for principal component analysis and hierarchical clustering by means of Perseus. Groups of interesting proteins were submitted to biological processes and pathways enrichment analysis, performed through the online tool DAVID Bioinformatics Resources v6.8 (Huang da et al., 2009a, b).

Statistical analyses
All data were analyzed using GraphPad Prism 7 software and presented as mean ± S.E.M. with 2-5 independent differentiations per cell line (indicated in the figure legend). Unpaired two-tailed t-test or Mann-Whitney U test were used to compare two groups, repeated measures ANOVA was employed for Scholl analysis. A P-value of < 0.05 was considered significant.

References to supplemental experimental procedures

Baker, M.J., Trevisan, J., Bassan, P., Bhargava, R., Butler, H.J., Dorling, K.M., Fielden, P.R., Fogarty, S.W., Fullwood, N.J., Heys, K.A., *et al.* (2014). Using Fourier transform IR spectroscopy to analyze biological materials. Nat Protoc *9*, 1771-1791.

Gil, J., Ramirez-Torres, A., Chiappe, D., Luna-Penaloza, J., Fernandez-Reyes, F.C., Arcos-Encarnacion, B., Contreras, S., and Encarnacion-Guevara, S. (2017). Lysine acetylation stoichiometry and proteomics analyses reveal pathways regulated by sirtuin 1 in human cells. J Biol Chem *292*, 18129-18144.

Gomes, C., Cunha, C., Nascimento, F., Ribeiro, J.A., Vaz, A.R., and Brites, D. (2019). Cortical Neurotoxic Astrocytes with Early ALS Pathology and miR-146a Deficit Replicate Gliosis Markers of Symptomatic SOD1G93A Mouse Model. Mol Neurobiol *56*, 2137-2158.

Huang da, W., Sherman, B.T., and Lempicki, R.A. (2009a). Bioinformatics enrichment tools: paths toward the comprehensive functional analysis of large gene lists. Nucleic Acids Res *37*, 1-13.

Huang da, W., Sherman, B.T., and Lempicki, R.A. (2009b). Systematic and integrative analysis of large gene lists using DAVID bioinformatics resources. Nat Protoc *4*, 44-57.

Miller, L.M., Bourassa, M.W., and Smith, R.J. (2013). FTIR spectroscopic imaging of protein aggregation in living cells. Biochim Biophys Acta *1828*, 2339-2346.

Roybon, L., Hjalt, T., Stott, S., Guillemot, F., Li, J.Y., and Brundin, P. (2009). Neurogenin2 directs granule neuroblast production and amplification while NeuroD1 specifies neuronal fate during hippocampal neurogenesis. PLoS One *4*, e4779.

Supplementary Table 1. Electrophysiological characteristics of human iPSC-derived hippocampal neurons

	Control 1	PS1	Control 2	APP
**n**	23	23	26	23
**V_{rest} (mV)**	-38.28 ± 2,201	-36.76 ± 2.20	-39.94 ± 1.93	-38.41 ± 2.00
**R_{input} (MΩ)**	3116 ± 802	2229 ± 312	2981 ± 485	3365 ± 504
**C (pF)**	1.060 ± 0.139	1.488 ± 0.206	1.666 ± 0.349	1.215 ± 0.250
**AP threshold (mV)**	-20.42 ± 1.12	-18.54 ± 0.95	-24.24 ± 0.73	-20.83 ± 1.24*
**AP amplitude (mV)**	37.19 ± 1.93	35.96 ± 2.15	46.81 ± 3.25	34.67 ± 2.96*
**AP rise time (ms)**	1.71 ± 0.14	1.70 ± 0.12	2.03 ± 0.34	2.25 ± 0.30
**½ AP amp. width (ms)**	2.04 ± 0.17	1.99 ± 0.17	2.47 ± 0.30	2.75 ± 0.37
**AHP peak (mV)**	19.47 ± 4.62	22.63 ± 1.73	20.89 ± 1.56	20.71 ± 2.21

Abbreviations: V_{rest}, resting membrane potential, R_{input}, input resistance, C, membrane capacitance. n=23-26. P values: * = P<0.05.

Statistical analysis of V_{rest} (p=07251), Ri_{nput} (p=0.4481) and C (p=0.4763) by Kruskal-Wallis test show no significant differences between any of the four lines.

Control 1 vs PS1:
AP threshold, Unpaired t-test: p=0.2076
AP amplitude, Mann-Whitney test: p=0.5276
AP rise time, Mann-Whitney test: p=0.8448
½ AP amp. width, Mann-Whitney test: p=0.7482
AHP peak, Mann-Whitney test: p=0.9826.

Control 2 vs APP:
AP threshold, Unpaired t-test: p=0.0186
AP amplitude, unpaired t-test: p=0.0088
AP rise time, Mann-Whitney test: p=0.4440
½ AP amp. width, Mann-Whitney test: p=0.8504
AHP peak, unpaired t-test: p=0.9461

Supplementary Table 6. Primary and secondary antibodies used for immunocyto- and immunohistochemistry.

Antibody	Species	Type	Dilution	Manufacturer	Reference
**Primary antibodies**					
AFP	Mouse	Monoclonal	1:500	Sigma-Aldrich	A8452
AT8	Mouse	Monoclonal	1:500	Thermo Fisher	MN1020
Beta-III-tubulin	Mouse	Monoclonal	1:500	Sigma-Aldrich	T8660
Calbindin	Mouse	Monoclonal	1:500	Sigma Aldrich	C9848
Calretinin	Rabbit	Polyclonal	1:500	Swant	CR7697
Doublecortin	Rabbit	Monoclonal	1:400	Cell Signalling	4604
Drebrin	Rabbit	Polyclonal	1:1000	Abcam	ab11068
GABA	Rabbit	Polyclonal	1:500	Thermo Fisher	PA5-32241
GFAP	Rabbit	Polyclonal	1:2000	DAKO	Z0334
NCAM	Mouse	Monoclonal	1:400	Santa Cruz	SC-106
Human nuclear antigen (hNuclei)	Mouse	Monoclonal	1:250	Merck Millipore	MAB1281
LEF1	Rabbit	Monoclonal	1:200	Cell Signalling	2230

MAP2	Chicken	Polyclonal	1:1000	Abcam	AB92434
NANOG, PE-conjugated	Mouse	Monoclonal	1:200	BD Biosciences	560483
O4	Mouse	Monoclonal	1:200	Gift from J. Goldman, Columbia University, NY	
OCT4	Mouse	Monoclonal	1:200	Merck Millipore	MAB4401
PAX6	Mouse	Monoclonal	1:400	Thermo Fisher	MA1-109
PROX1	Rabbit	Monoclonal	1:400	Abcam	ab199359
Sendai virus	Chicken	Polyclonal	1:1000	Abcam	ab33988
SMA	Mouse	Monoclonal	1:200	Sigma Aldrich	A2547
Synaptophysin	Mouse	Monoclonal	1:1000	Merck Millipore	MAB5258
TBR1	Rabbit	Polyclonal	1:500	Merck Millipore	AB10554
TRA-1-81	Mouse	Monoclonal	1:200	Thermo Fisher	41-1100
ZBTB20	Rabbit	Polyclonal	1:200	Sigma Aldrich	HPA016815
**Secondary antibodies**					
Anti-chicken Alexa Fluor® 488	Donkey	Polyclonal	1:400	Jackson Immuno Research	703-545-155
Anti-mouse Alexa Fluor® 488	Donkey	Polyclonal	1:400	Thermo Fisher	A21202
Anti-rabbit Alexa Fluor® 488	Donkey	Polyclonal	1:400	Thermo Fisher	A21206
Anti-mouse Alexa Fluor® 555	Donkey	Polyclonal	1:400	Thermo Fisher	A31570
Anti-rabbit Alexa Fluor® 555	Donkey	Polyclonal	1:400	Thermo Fisher	A31572
Anti-chicken Alexa Fluor® 647	Donkey	Polyclonal	1:400	Merck Millipore	AP194SA6
Anti-mouse Alexa Fluor® 647	Donkey	Polyclonal	1:400	Thermo Fisher	A31571
Anti-chicken Alexa Fluor® 647	Goat	Polyclonal	1:500	Jackson Immuno Research	103-605-155
Anti-mouse Alexa Fluor® 647	Goat	Polyclonal	1:500	Jackson Immuno Research	115-605-146
Anti-mouse Alexa Fluor® 488	Goat	Polyclonal	1:500	Jackson Immuno Research	115-545-146
Anti-rabbit Alexa Fluor® Cy3	Goat	Polyclonal	1:500	Jackson Immuno Research	111-165-144
Streptavidin, Alexa Fluor™ 647 conjugate			1:400	Thermo Fisher	S32357

Supplementary Table 7. List of primer sequences used in qRT-PCR for microRNA expression

microRNA	Target Sequence
hsa-miR-21-5p	5’- UAGCUUAUCAGACUGAUGUUGA-3’
hsa-miR-29a-5p	5'ACUGAUUUCUUUUGGUGUUCAG-3’
hsa-miR-124-3p	5'UAAGGCACGCGGUGAAUGCC-3’
hsa-miR-125b-5p	5’-UCCCUGAGACCCUAACUUGUGA-3’
U6 snRNA	Reference gene
RNU1A1	Reference gene

Supplementary Table 8. Primary and secondary antibodies used for Western Blotting

Antibody	Species	Type	Dilution	Manufacturer	Reference
**Primary antibodies**					
Actin	Mouse	Monoclonal	1:1000	Sigma	A5316
AT8	Mouse	Monoclonal	1:1000	Thermo Fisher	MN1020
Drebrin	Rabbit	Polyclonal	1:1000	Abcam	ab11068
MAP2	Mouse	Monoclonal	1:1000	Sigma	M4403
PSD-95	Mouse	Monoclonal	1:1000	Merck Millipore	MAB1596
Synaptophysin	Mouse	Monoclonal	1:1000	Merck Millipore	MAB5258
**Secondary antibodies**					
Anti-mouse IGG HRP-conjugated	Goat	Polyclonal	1:1000	R&D Systems	HAF007
Anti-rabbit IGG HRP-conjugated	Goat	Polyclonal	1:1000	R&D Systems	HAF008

a

Diagnosis	Molecular analysis	Age at biopsy	Gender	iPSC line ID
Ctrl [Non demented healthy individual (NDHI)]	NA	65	Male	CSC-36C, D and E
Ctrl (NDHI)	NA	56	Female	CSC-37N and 37R
Ctrl (NDHI; Target ALS, cat# ND50004)	NA	49	Female	TALSCTRL15.12
See Assini et al., Neurology, 2003; 60;150	PS1, p.R278K	53	Male	CSC-12D, E and F
See Sorbi et al., Nature Genetics, 1993, v4	APP, p.V717I	50	Female	CSC-17F, F.1 and F.2

b

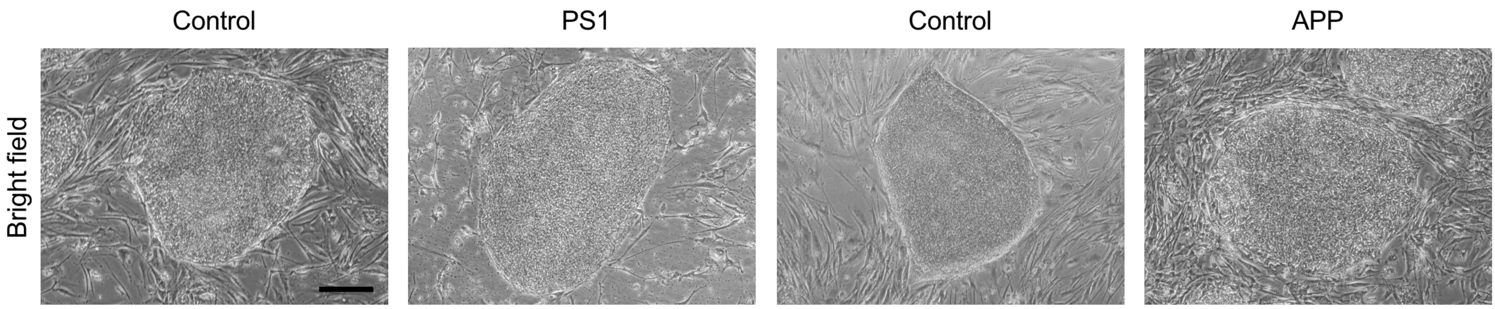

c

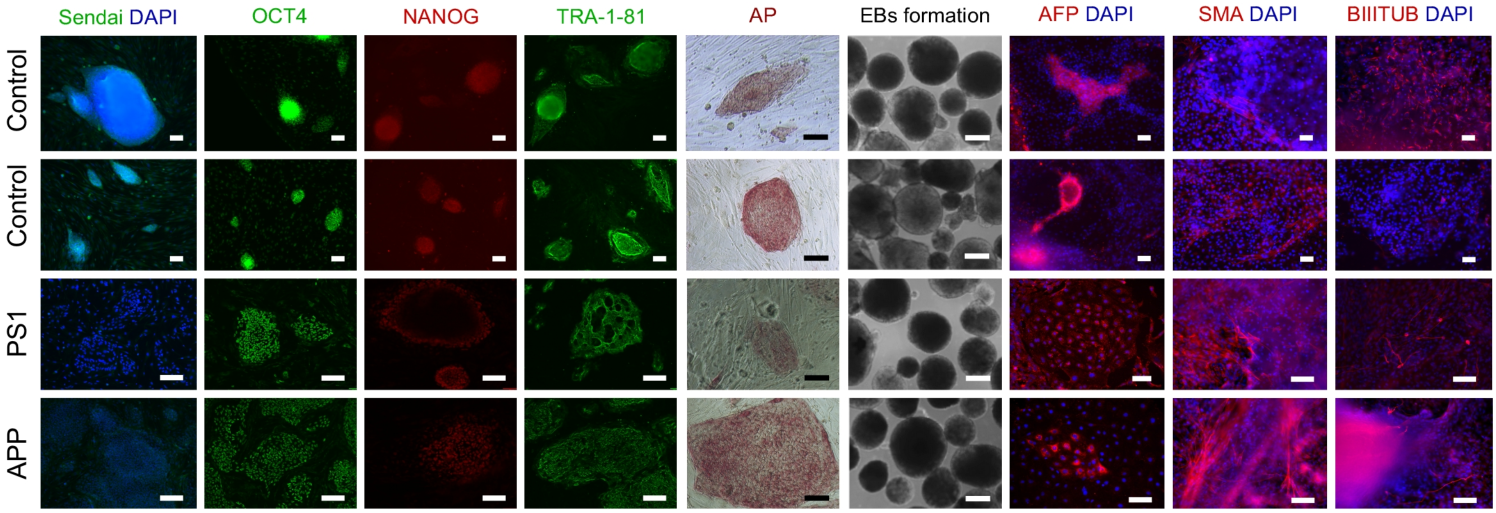

d

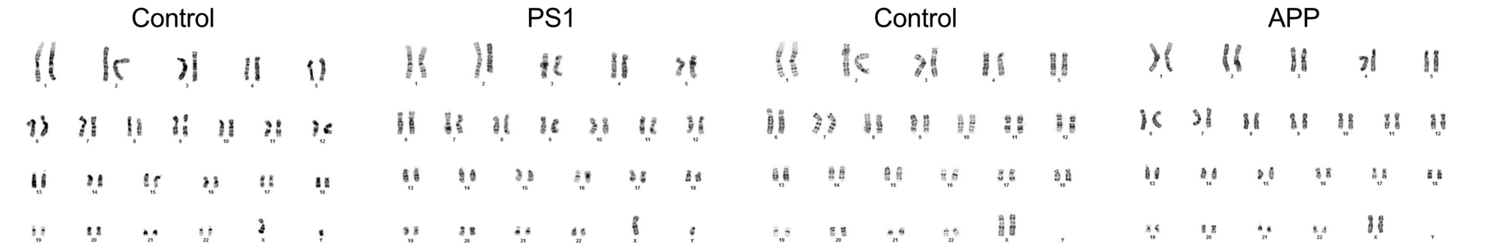

e

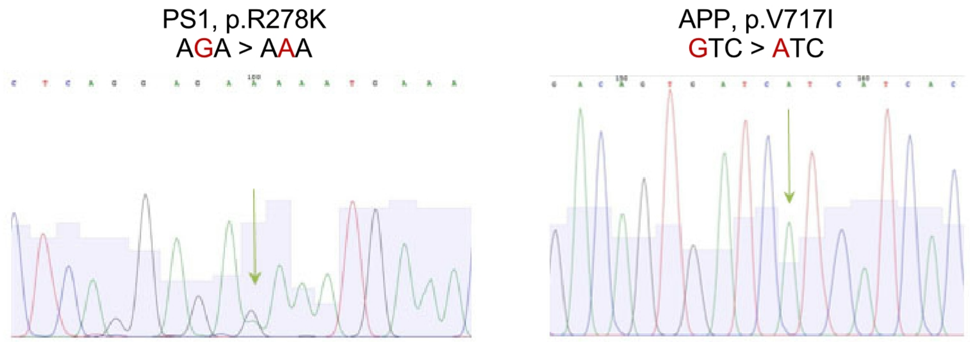

f

CSC-12 and parental fibroblasts

Marker	Allele(s)	
AMEL	X	Y
CSF1PO	10	11
D13S317	8	11
D16S539	10	12
D21S11	28	31.2

CSC-17 and parental fibroblasts

Marker	Allele(s)	
AMEL	X	
CSF1PO	12	
D13S317	10	12
D16S539	9	13
D21S11	29	30

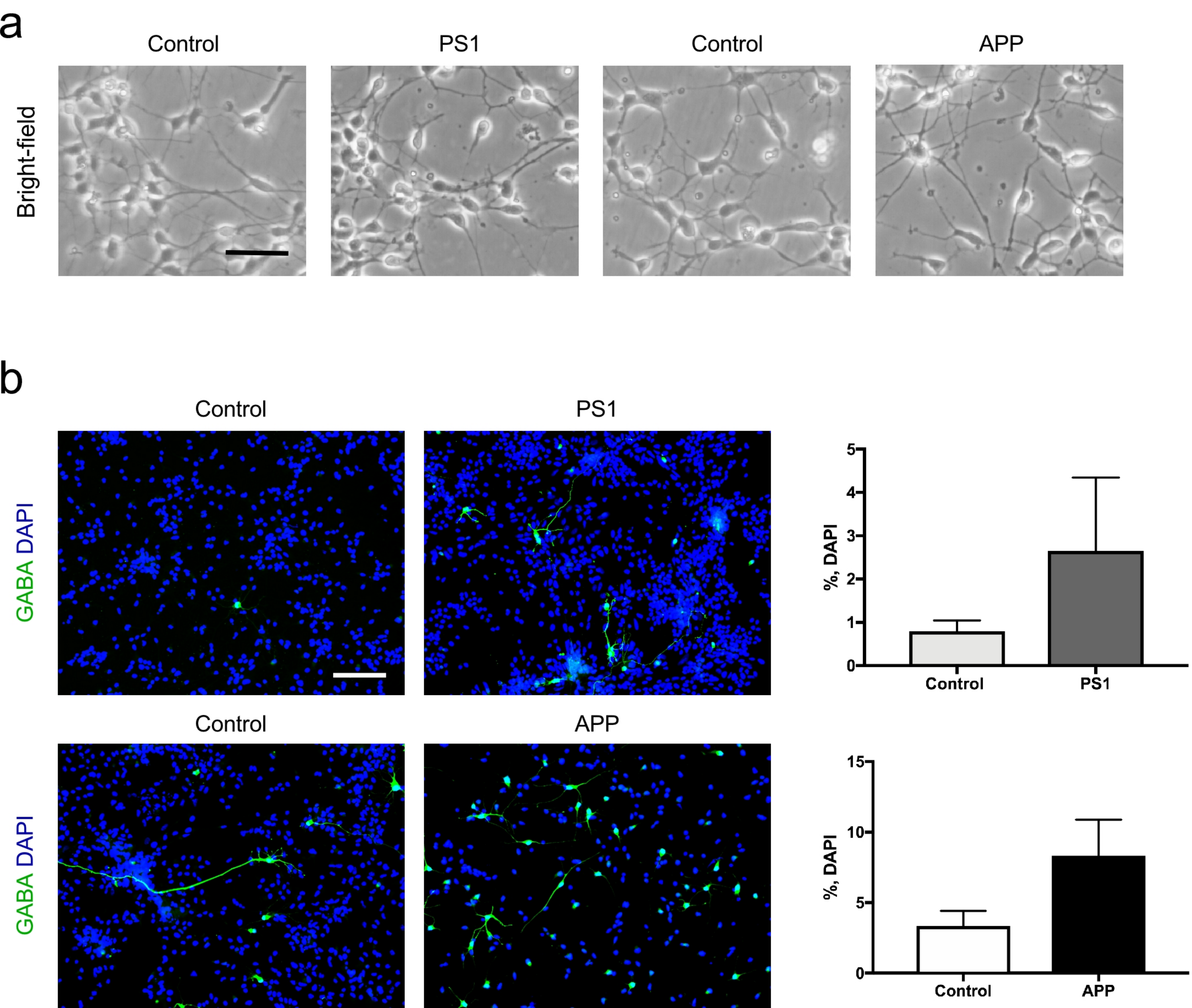
a
Control
PS1
Control
APP
Bright-field
b
Control
PS1
GABA DAPI
5
4
3
2
1
0
%, DAPI
Control
PS1
Control
APP
GABA DAPI
15
10
5
0
%, DAPI
Control
APP

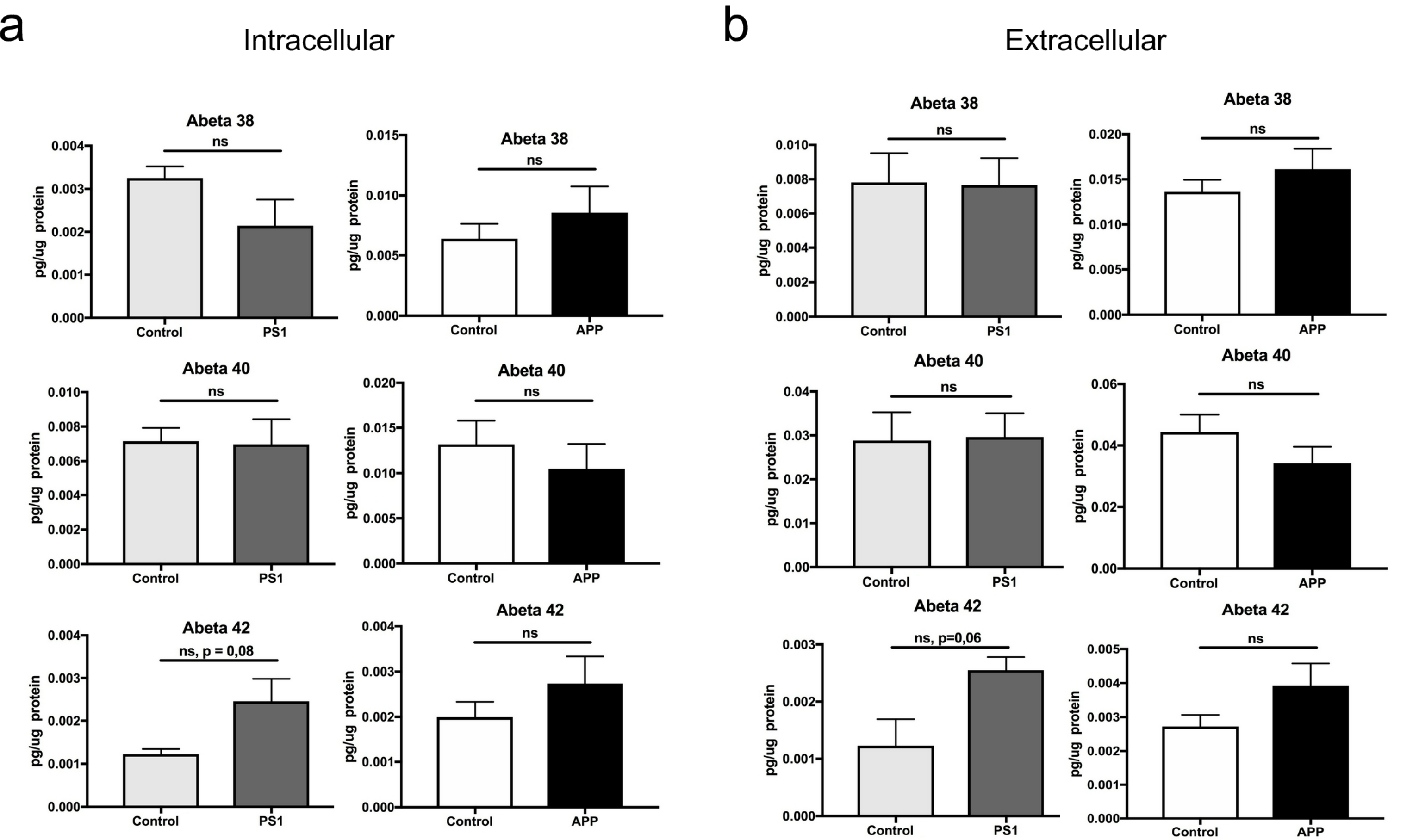
a
Intracellular
b
Extracellular
Abeta 38
Abeta 40
Abeta 42
ns
ns, p = 0,08
ns, p=0,06
pg/ug protein
Control
PS1
APP

Neuronal cells

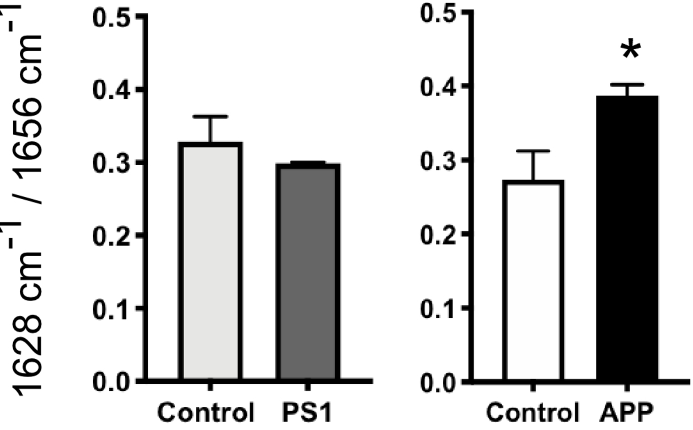

Supplement: Document S2. Article plus Supplemental Information [file mmc6.pdf]
